# 1-Oxo-3,4-dihydroisoquinoline-4-carboxamides as novel druglike inhibitors of poly(ADP-ribose) polymerase (PARP) with favourable ADME characteristics

**DOI:** 10.1080/14756366.2021.1972993

**Published:** 2021-09-06

**Authors:** Alexander Safrygin, Petr Zhmurov, Dmitry Dar’in, Sergey Silonov, Mariia Kasatkina, Yulia Zonis, Maxim Gureev, Mikhail Krasavin

**Affiliations:** a Saint Petersburg State University, Saint Petersburg, Russian Federation; b JSC BIOCAD, Saint Petersburg, Russian Federation; c Digital Biodesign and Personalized Healthcare Research Center, Sechenov First Moscow State Medical University, Moscow, Russian Federation; d Immanuel Kant Baltic Federal University, Kaliningrad, Russian Federation

**Keywords:** Poly(ADP-ribose) polymerase, PARP1/2 selectivity, NAD^+^ mimetics, 3,4-dihydroisoquinolone-4-carboxamides, castagnoli-cushman reaction, druglikeness

## Abstract

A novel 3,4-dihydroisoquinol-1-one-4-carboxamide scaffold was designed as the basis for the development of novel inhibitors of poly(ADP-ribose) polymerase (PARP). Synthesis of 3,4-dihydroisoquinol-1-one-4-carboxylic acids was achieved using the previously developed protocol based on the modified Castagnoli-Cushman reaction of homophthalic anhydrides and 1,3,5-triazinanes as formaldimine synthetic equivalents. Employment of 2,4-dimethoxy groups on the nitrogen atom of the latter allowed preparation of 2,3-unsubatituted 3,4-dihydroquinolone core building blocks. Iterative synthesis and in vitro biological testing of the amides resulting from the amidation of these carboxylic acids allowed not only drawing important structure-activity generalisations (corroborated by *in silico* docking simulation) but also the identification of the lead compound, 4-([1,4'-bipiperidine]-1'-carbonyl)-7-fluoro-3,4-dihydroisoquinolin-1(2*H*)-one, as the candidate for further preclinical development. The lead compound as well as its des-fluoro analog were compared to the approved PARP1 inhibitor, anticancer drug Olaparib, in terms of their molecular characteristics defining druglikeness as well as experimentally determined ADME parameters. The newly developed series demonstrated clear advantages over Olaparib in terms of molecular weight, hydrophilicity, human liver microsomal and plasma stability as well as plasma protein binding. Further preclinical investigation of the lead compound is highly warranted.

## 1. Introduction

Poly(ADP-ribose) polymerase (PARP) enzymes (numbering seventeen in total) have drawn considerable attention as drug targets in the last decade owing to the clinical success of non-selective PARP1/2 inhibitors *Olaparib*, *Talazoparib*, *Niraparib* and *Rucaparib* approved for cancer treatment^1^. PARP1 and PARP2 are two key enzymes that are critical for repairing single-strand breaks (‘nicks’) in the DNA – a mechanism which is critical for the survival of both normal and cancer cells^2^. During the cell division cycle, single-strand DNA breaks lead to double-strand DNA breaks which can, again, be repaired by the cell’s DNA repair machinery, including PARP enzymes. It is generally accepted that, despite the non-selectivity of the approved PARP inhibitors, it is the inhibition of PARP1 that is responsible for the manifestation of their clinical efficacy^3^. Therefore, attempts have been made towards the development of ‘clean’, selective PARP1 inhibitors such as compound NMS-P118 developed by Nerviano Medical Sciences jointly with Chemical Diversity Research Institute^4^. The inhibition of PARP1 alone can raise the effectiveness of chemotherapy and radiation therapy^5^. Normal cells do not divide as frequently as cancer cells which allows them to survive PARP1 inhibition. However, tumour cells with BRCA1, BRCA2 or PALB2 mutations that are synthetically lethal with PARP1 inhibition^6^ are efficiently killed by the drugs of this class.

The pharmacological consequences of PARP2 targeting remain to be unravelled which is somewhat hampered by the scarcity of selective PARP2 inhibitors as pharmacological tools^7^^,^^8^. PARP2 inhibition has been shown to inhibit androgen receptor signalling which may be useful in the prostate cancer therapy^9^.

**Figure 1.**
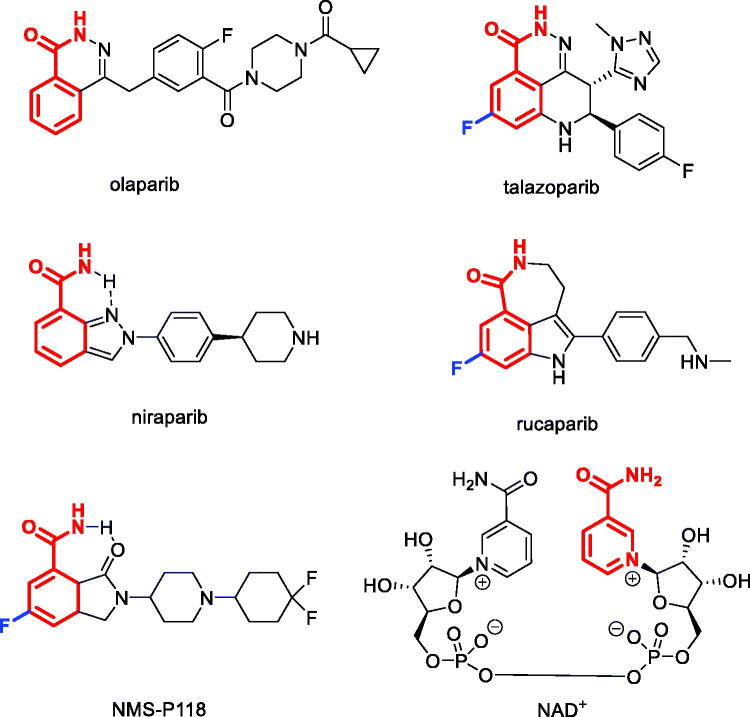
Clinically used PARP1 inhibitors, advanced clinical candidate NMS-P118 (NAD+-mimicking motif is highlighted in red, NAD^+^ structure shown).

The majority of PARP1 inhibitors, including those shown in Figure 1, were designed to mimic the nicotinamide moiety of NAD^+^ (from which the adenine ribose unit of poly(ADP-ribose) originates) with which the inhibitors compete for the NAD^+^-binding site of PARP1. This mimicry is achieved *via* the use of either a rotationally constrained primary benzamide (as in Niraparib and NMS-P118) or a benzamide motif embedded in a ring (as in Olaparib, Talazoparib and Rucaparib). Another characteristic feature noticeable in some of the advanced PARP1 inhibitors is the presence of a fluorine atom (highlighted in blue) in the *meta*-position of the NAD^+^-mimicking benzamide moiety (Figure 1). This ‘magic fluorine’ has been shown to enhance binding to the target^4^.

**Figure 2.**
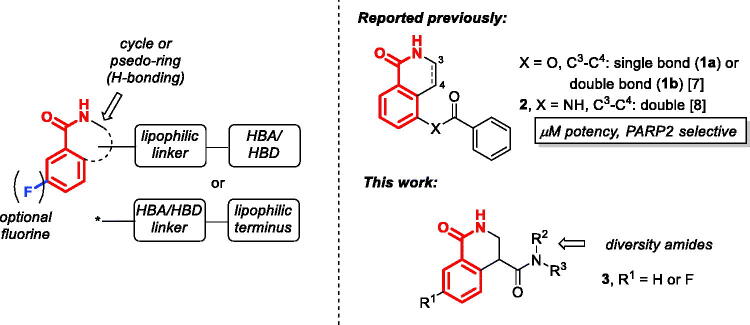
The general PARP1 pharmacophore model; micromolar, PARP2-selective inhibitors **1**–**2** reported earlier and 1-oxo-3,4-dihydroisoquinoline-4-carboxamides derivatives **3** investigated in this work.

Analysis of the structures of PARP1 inhibitors shown in Figure 1 as well as those reported in the literature^10^ allows one to develop the following intuitive pharmacophore model. The cyclic or conformationally constrained *cis*-benzamide optionally substituted with fluorine in the *meta*-position can be substituted with a periphery substituent in the ‘western’ portion of the molecule broadly defined either as a combination of a lipophilic linked and a terminal polar hydrogen bond accepting (HBA) or donating (HBD) group or a polar (HBA/HBA) linker capped with a lipophilic terminal moiety. Considering the significant variation in the nature of the ‘western’ motif in the advanced PARP1 inhibitors, for each new chemotype, the preferred substituent ought to be identified *via* iterative experimental optimisation supported by the computer-aided drug design^4^. Some of the PARP inhibitors reported in the literature do not conform to this general pharmacophore model and, unsurprisingly, are not potent (IC_50_ in the micromolar range *vs.* nanomolar values for clinically used PARP1 inhibitors) and display selectivity towards PARP2. For instance, fairly simple benzoate (**1**) and benzamide derivatives of (dihydro)isoquinolone are illustrative examples. Indeed, dihydroisoquinolone **1a** was reported to have PARP1/PARP2 IC_50_ of 13/0.8 µM and selectivity index (SI) 16.3. Isoquinolone **1b** displayed higher PARP2 selectivity: PARP1/PARP2 IC_50_ 9.0/0.15 µM (SI 60.0)^7^. Benzamido isoquinolone **2** reported 3 years later displayed a similar profile (PARP1/PARP2 IC_50_ 13.9/1.5 µM, SI 9.3)^8^. We reasoned that the simple, NAD^+^-mimicking 3,4-dihydroisoquinolone core could be employed in the design of PARP1 inhibitors if the inhibitor molecular topology is adjusted to conform to the general PARP1 inhibitor pharmacophore model (*vide supra*). To this end we set off to explore 1-oxo-3,4-dihydroisoquinoline-4-carboxamide derivatives **3** – without or with the ‘magic fluorine’ substitution, respectively (Figure 2). In this Article, we report our findings obtained in the course of realising this idea.

## 2. Results and discussion

### 2.1. Chemistry

**Scheme 1.**
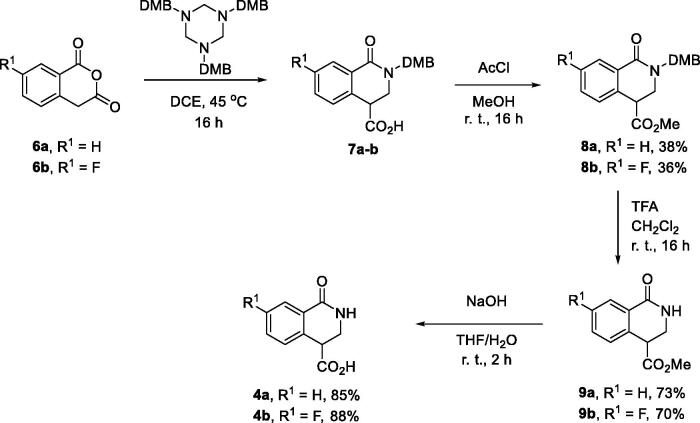
Synthesis of 1-oxo-3,4-dihydroisoquinoline-4-carboxylic acids **4a-b**.

The synthesis of compounds belonging to series **3** required access to gram quantities of carboxylic acids **4**. Preparation of carboxylic acid **4a** (R^1^ = H) had been reported in the patent literature and involved autoclave hydrogenation of isoquinolone-4-carboxylic acid methyl ester, with subsequent hydrolysis^11^. For this work, we devised a practically convenient method which does not mandate the use of a specialised equipment. The method is reliant on the earlier reported synthesis of 3-unsubstituted isoquinolone-4-carboxylic acid derivatives *via* the Castagnoli-Cushman reaction of 1,3,5-triazinanes as formaldimine synthetic equivalents^12^. Reaction of 1,3,5-tris(2,4-dimethoxybenzyl)-1,3,5-triazinane (**5**) with homophthalic (**6a**) or commercially available 7-fluorohomopthalic anhydride (**6b**) furnished carboxylic acids **7a** and **7 b**, respectively; the latter were esterified for easier chromatographic purification and the corresponding esters **8a** and **8b** were obtained in 38% and 36% yield over two steps. Removal of the DMB (2,4-dimethoxybenzyl) group by TFA in dichloromethane at room temperature was clean and high-yielding. Esters **9a-b** thus obtained were subjected to basic hydrolysis to furnish the corresponding carboxylic acids **4a-b** in excellent yield (Scheme 1).

**Scheme 2.**
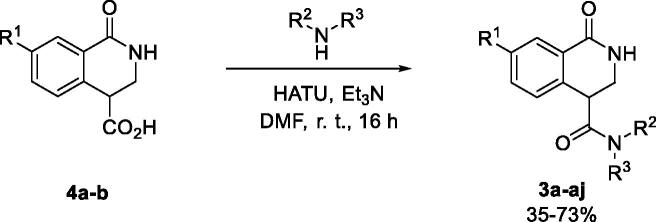
Synthesis of 1-oxo-3,4-dihydroisoquinoline-4-carboxamides **3a-aj**.

**Scheme 3.**
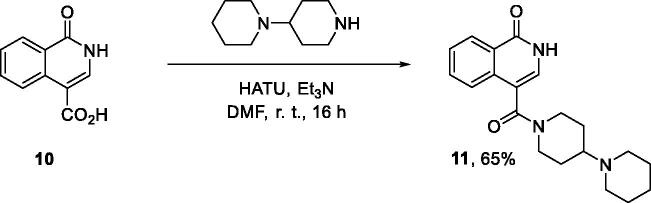
Synthesis of 1-oxoisoquinoline-4-carboxamide **11**.

Carboxylic acids **4a-b** were converted to a set of 36 amides **3a-ak** in moderate to good yields (see Table 1 in Section 2.2 for exact chemical yields) using 1-[bis(dimethylamino)methylene]-1*H*-1,2,3-triazolo[4,5-*b*]pyridinium 3-oxide hexafluorophosphate (HATU) as an activating agent for the carboxylic acid function (Scheme 1 and 2). These amides were submitted for the *in vitro* evaluation of their inhibitory potency towards PARP1 and PARP2 enzymes.

**Table 1.** Inhibitory activity of compounds **3a-aj** *vs.* PARP1 and PARP2 enzymes.
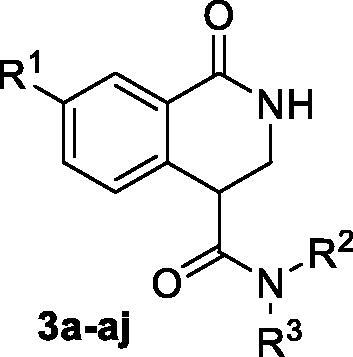

Compound	R^1^	R^2^NR^3^	PARP1		PARP2 IC_50_, nM^*a*^ (r^2^)	SI^*b*^
			% inhibition at 1 µM	IC_50_, nM^*a*^ (r^2^)		
**3a**	H	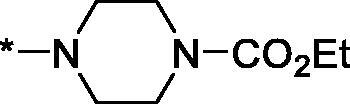	26.3 ± 7.7	–	–	–
**3b**	H	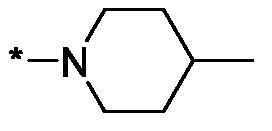	42.7 ± 0.9	–	–	–
**3c**	H	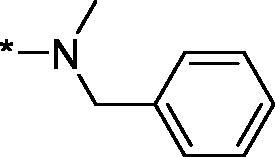	18.6 ± 13.2	–	–	–
**3d**	H	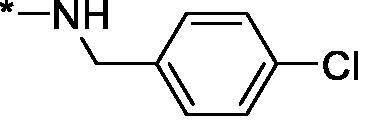	7.4 ± 8.7	–	–	–
**3e**	H	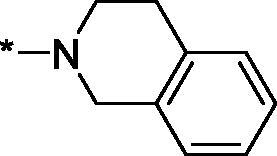	56.0 ± 7.5	–	–	–
**3f**	H	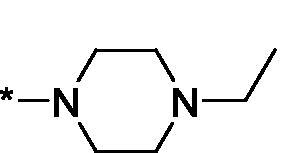	26.2 ± 9.7	–	–	–
**3g**	H	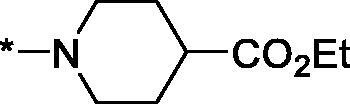	47.6 ± 4.1	–	–	–
**3h**	H	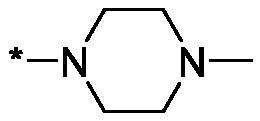	27.3 ± 6.0	–	–	–
**3i**	H	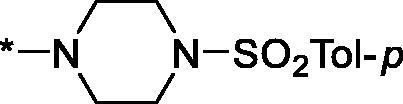	28.9 ± 5.5	–	–	–
**3j**	H	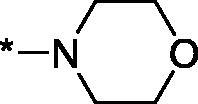	57.6 ± 5.8	–	–	–
**3k**	H	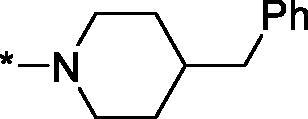	45.1 ± 1.7	–	–	–
**3l**	H	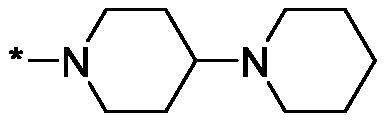	85.2 ± 2.6	156 ± 5.8 (0.9982)	70.1 ± 7.6 (0.9912)	2.23
**3m**	H	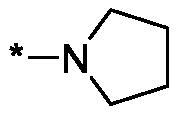	31.2 ± 3.4	–	–	–
**3n**	H	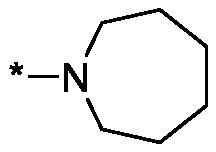	35.4 ± 5.1	–	–	–
**3o**	H	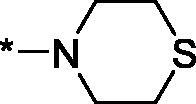	47.4 ± 3.7	–	–	–
**3p**	H	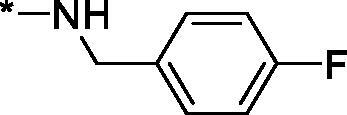	27.2 ± 4.3	–	–	–
**3q**	H	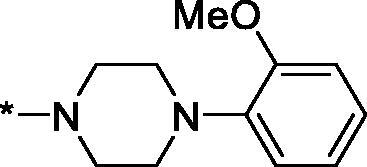	32.9 ± 2.4	–	–	–
**3r**	H	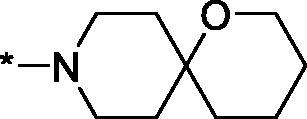	28.5 ± 7.1	–	–	–
**3s**	H	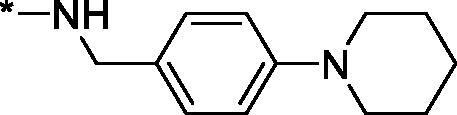	12.5 ± 8.5	–	–	–
**3t** ^*c*^	H	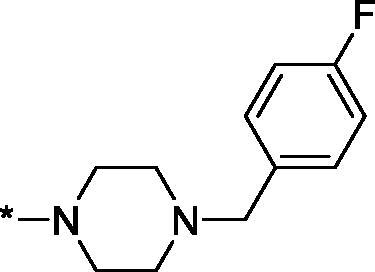	43.3 ± 5.9	–	–	–
**3u** ^*c*^	H	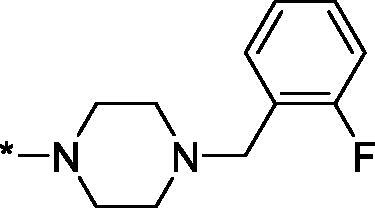	31.9 ± 2.0	–	–	–
**3v**	H	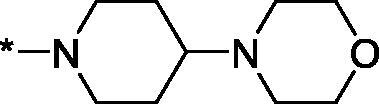	33.2 ± 4.8	–	–	–
**3w** ^*c*^	H	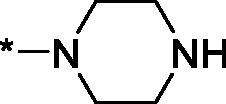	66.9 ± 1.5	–	–	–
**3x**	H	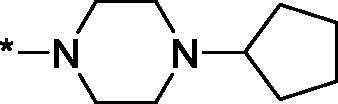	44.6 ± 2.9	–	–	–
**3y**	H	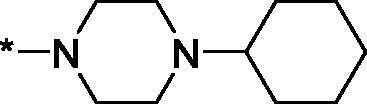	54.5 ± 4.6	–	–	–
**3z**	H	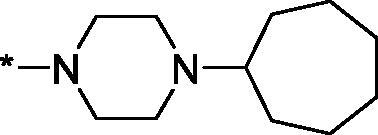	45.2 ± 7.4	–	–	–
**3aa** ^*c*^	F	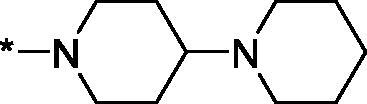	91.8 ± 0.7	63.1 ± 5.3 (0.9981)	29.4 ± 4.5 (0.9958)	2.17
**3ab**	F	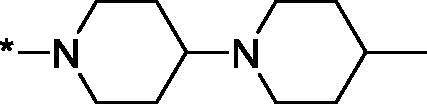	93.9 ± 0.2	82 ± 6.9 (0.9961)	28.8 ± 4.2 (0.9950)	2.82
**3ac**	F	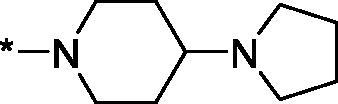	95.8 ± 0.7	69.5 ± 8.8 (0.9972)	29.6 ± 6.1 (0.9917)	2.3
**3ad**	F	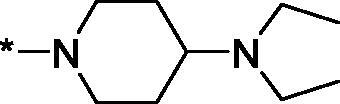	92.6 ± 1.1	128 ± 7.1 (0.9957)	40.4 ± 5.8 (0.9985)	3.23
**3ae**	F	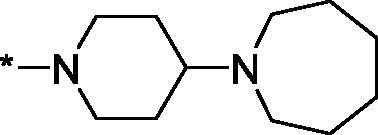	96.6 ± 0.3	55.0 ± 9.9 (0.9894)	20.6 ± 2.5 (0.9998)	2.61
**3af**	F	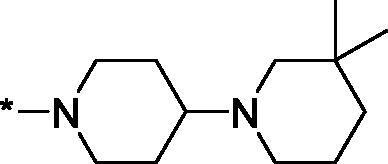	96.9 ± 0.5	95.6 ± 11.5 (0.9947)	19.4 ± 3.7 (0.9992)	5.05
**3ag**	F	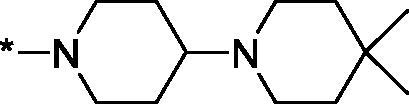	95.0 ± 0.9	102 ± 9.4 (0.9956)	22.0 ± 5.6 (0.9805)	4.63
**3ah**	F	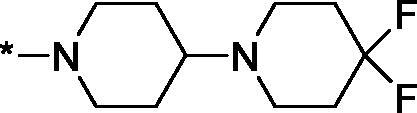	81.9 ± 1.8	371 ± 42 (0.9719)	83.1 ± 6.9 (0.9841)	4.46
**3ai**	F	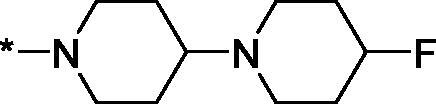	93.7 ± 0.8	148 ± 17 (0.9873)	42.0 ± 2.3 (0.9901)	3.52
**3aj**	F	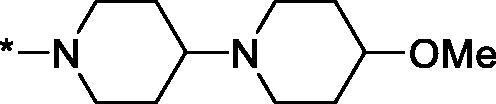	90.5 ± 0.1	266 ± 25 (0.9833)	44.1 ± 3.9 (0.9989)	6.05
**11** ^*d*^	H	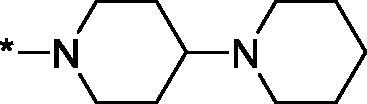	49.1 ± 2.6	–	–	–
Olaparib			98.4 ± 2.1	2.8 ± 0.3 (0.9991)	0.7 ± 0.2 (0.9923)	3.98

^a^Mean (*n* = 3) ± SD.

^b^Selectivity index (PARP2 over PARP1).

^c^Isolated as a trifluoroacetate salt after HPLC.

^d^Synthesised by amidation of 1-oxoisoquinoline-4-carboxylic acid **10**.

### 2.2. Inhibitory activity against PARP1 and PARP2 in vitro

The thirty-six 1-oxo-3,4-dihydroisoquinoline-4-carboxamides **3a-aj** and 1-oxoisoquinoline-4-carboxamide **11** were tested for inhibitory activity towards PARP1 and PARP2 using the commercially available colorimetric activity assay kit from BPS Bioscience (San Diego, CA) in full accordance of the supplier’s method description^13^^,^^14^. The initial screening was performed against PARP1 at 1 µM concentration of each compound, in triplicate (*n* = 3) measurements. Compounds which displayed over 80% inhibition of the enzyme activity were tested in dose-response mode (*n* = 3) against PARP1 and PARP2 using Olaparib as the reference inhibitor in order to determine the compounds’ half-maximal inhibitory concentration (IC_50_) and assess the isoform selectivity.

As one can see from the data summarised in Table 1, the unsubstituted (R^1^ = H) 1-oxo-3,4-dihydroisoquinoline-4-carboxamides **3a-z** displayed fairly ‘tight’ structure-activity relationships with respect to the amide portion of the molecule. In fact, the majority of the carboxamides did not pass the “80% inhibition at 1 µM” threshold in order to be progressed to the IC_50_ determination stage. We estimate that these compounds are approximately of the same potency as (3,4-dihydro)isoquinolones **1a-b** and **2** (*vide supra*, Figure 2). However, our goal was to reach into the nanomolar range of half-maximal inhibitory concentrations. To our delight, compound **3l** displayed a markedly potent inhibition of PARP1 (85.2% at 1 µM) which translated into the IC_50_ of 156 nM *vs.* PARP1 and about two-fold selectivity towards PARP2 (IC_50_ = 70.1 nM). Two striking observations further speak for the ‘tightness’ of the SAR displayed by this series. Specifically, compounds **3y** (an isostere of **3l**) and **3v** (an oxa-analog of **3l**) are significantly less potent.

Having defined the type of the preferred carboxamide appendage (generally speaking, piperidine bearing a dialkylamino substituent, preferably cyclic, in position 4), we focussed on screening various versions of the appropriately substituted piperidines using the 7-fuoro-substituted core (i.e. *via* the amidation of carboxylic acid **4b**). The effect of the ‘magic fluorine’ substitution is clearly evident by comparing compounds **3aa** and **3l**. The former displayed a ∼2.5-fold better potency compared to the latter although the PARP2/PAPR1 selectivity remained roughly the same.

Modifications of the peripheral cyclic amine did not lead to a significant improvement of the PARP1 potency. Diethylamino compound **3ad** was not significantly different in potency and selectivity from compound **3 l**. Ring-contracted and ring-expanded analogs **3ac** and **3ae**, respectively, displayed similar profiles to that of compound **3aa**. Although compound **3ae** possesses the best activity profile among the compounds investigated in this study, we attribute the marginal improvement of potency compared to compound **3aa** to the lipophilic efficiency (LE)^17^ resulting from the introduction of an additional methylene unit. Thus, compound **3aa** can be regarded as the lead structure identified in this study. Although its potency is an order of magnitude lower than that of the clinically used Olaparib, compound **3aa** possesses lower molecular weight and excellent ADME characteristics (*vide infra*) and thus can be considered a frontrunner candidate for further preclinical development.

Interestingly, various substitutions around the distal piperidine ring of **3aa** (i.e. those present compounds **3ab** and **3af-aj**) – although had some negative bearing on the PARP1 potency – did not affect significantly the PARP2 activity. Indeed, compound **3af** can be regarded as the most potent and selective PARP2 inhibitor whose use can be envisioned as a tool compound for further investigation of the fundamental role of PARP2 and its inhibition in various cellular processes. The highest PARP2 selectivity displayed by compound **3aj** is also noteworthy.

It is evident that the flat, isoquinolone scaffold of similar topology is not suitable for a potent PARP inhibitor design. Indeed, compound **11** (the 3,4-dehydro analog of compound **3 l** or its 7-fluoro version, **3aa**) displayed an approximately 10-fold lower potency.

### 2.3. Structure-activity relationships rationalised through docking simulation

We attempted to rationalise several notable SAR observations made in the previous section of this Article by performing docking simulation as well as binding energy calculations for pairwise comparison of certain analogues. These studies were conducted for both enantiomers of each compound and yielded similar results. For brevity, results for only one enantiomer are shown.

#### 2.3.1. Comparison of frontrunner compound 3aa and its des-fluoro analogue 3l: the ‘magic fluorine effect’

**Figure 3.**
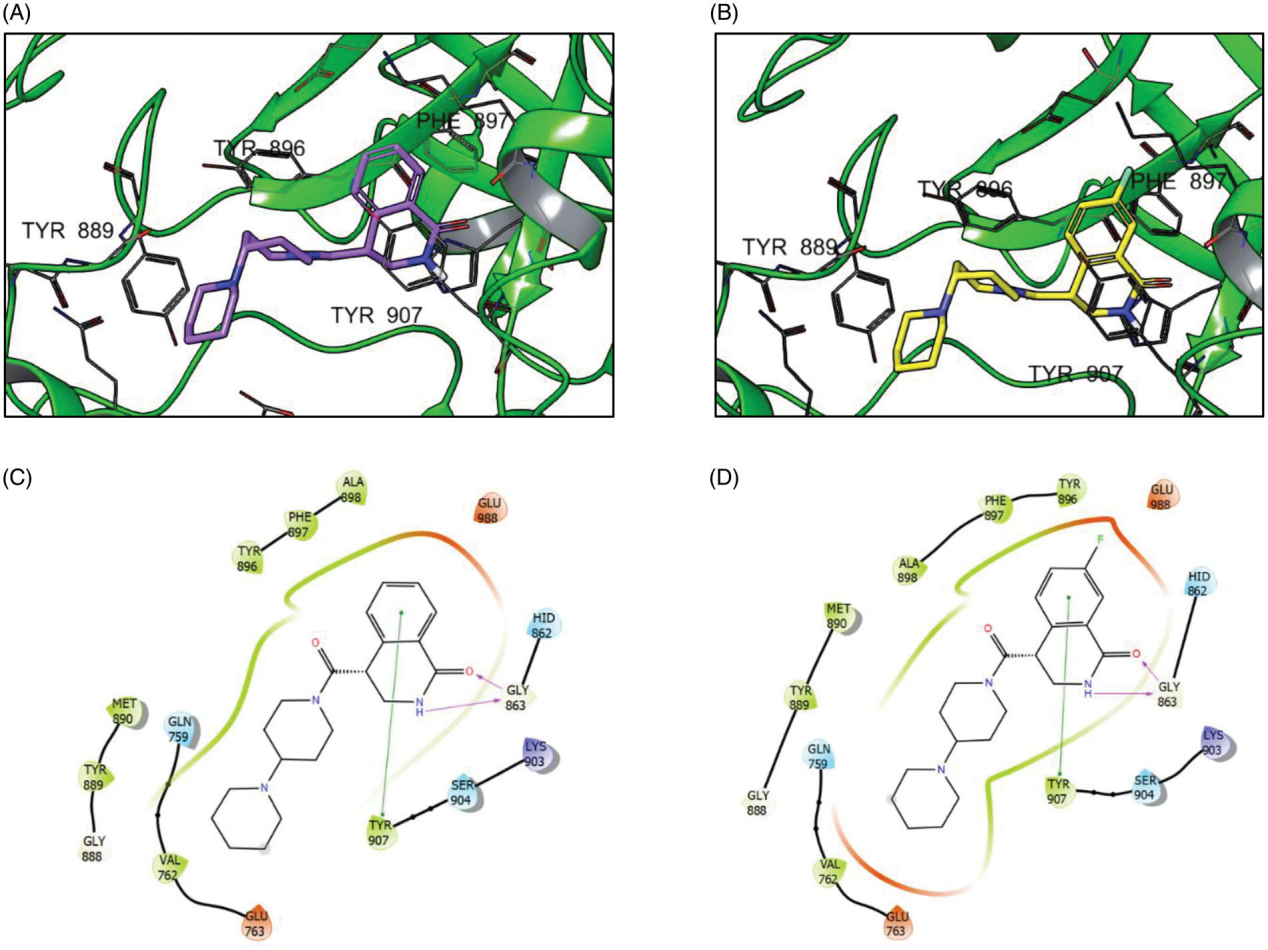
Binding poses of compounds **3 l** (A) and **3aa** (B) with PARP1 and the respective interaction diagrams (C and D).

Docking simulation of both compounds revealed that their docking poses are, not unexpectedly, very similar (Figure 3). However, the important contacts with Tyr896, Ala898 and Phe897 residues are significantly enhanced in the 7-fluoro analogue (**3aa**). Moreover, the contacts with charged Glu988 residue (Coulomb interactions) appear to be enhanced. Analysis of the scoring function components (Table 2) clearly supports these *in silico* observations regarding the ‘magic fluorine’ effect.

**Table 2.** Docking score values for compounds **3 l** and **3aa** and its components.

Compound	GlideScore (Kcal/mol)	Lipophilic interactions (Kcal/mol)	Coulomb interactions (Kcal/mol)
**3aa**	−9.91	−3.31	−9.75
**3l**	−8.57	−2.82	−5.55

#### 2.3.2. Comparison of compounds 3 l and 11: the importance of saturated, 3,4-dihydroquinolone scaffold

**Figure 4.**
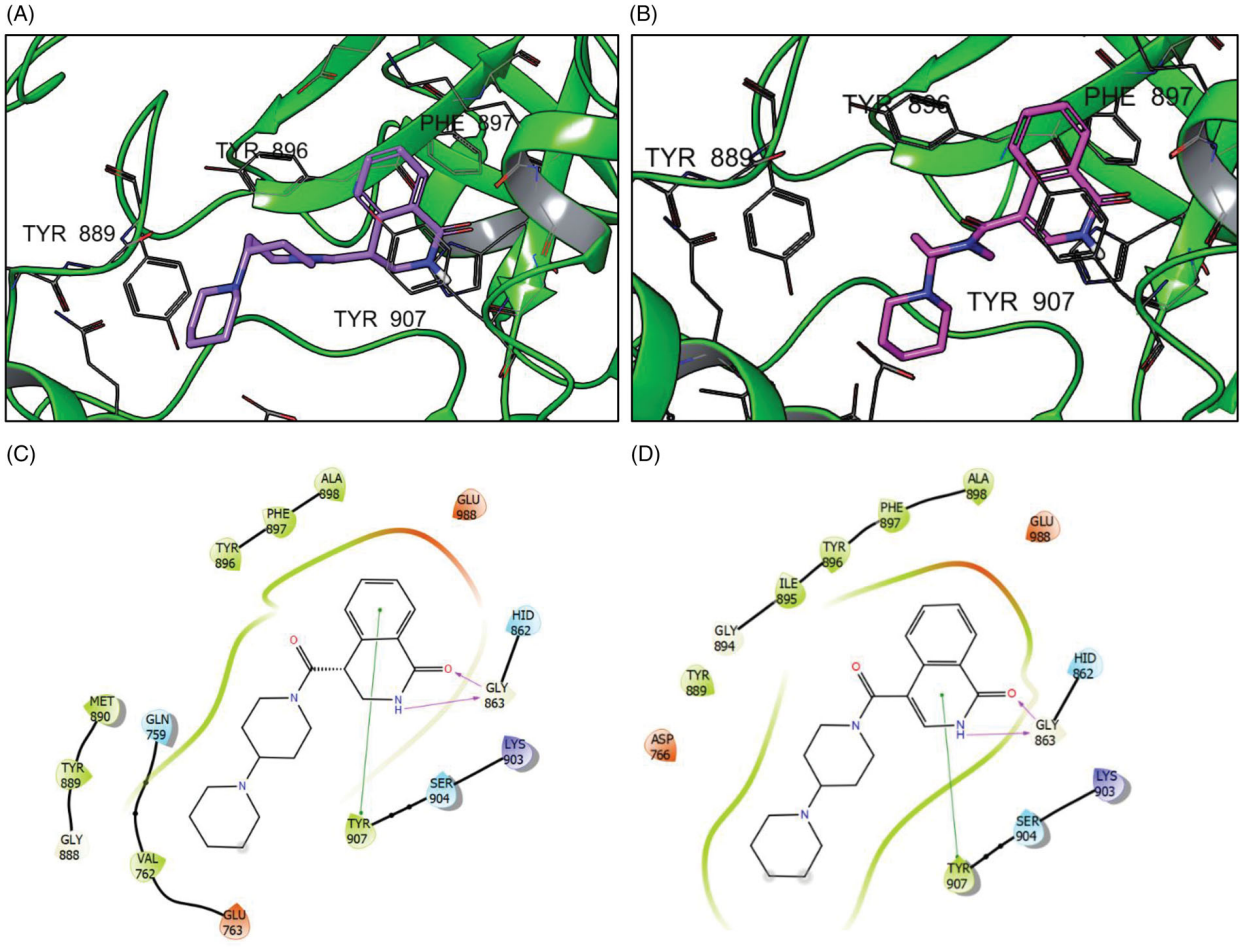
Binding poses of compounds **3 l** (A) and **11** (B) with PARP1 and the respective interaction diagrams (C and D).

Aromatisation of the scaffold in compound **11** leading to its increase planarity also induces significant conformational changes in the inhibitors structure and alters its binding to the target. The binding pose of compound **11** in the binding cleft of PARP1 analysed by docking simulation demonstrated the loss of hydrophobic contacts with Met890, Tyr889 and Val762 amino acid residues by compound **11** *vs.* compound **3l** (Figure 4). This observation is highlighted by the scoring function component analysis showing the loss of the binding affinity to be associated mostly with the hydrophobic and coulombic energy terms (Table 3).

**Table 3.** Docking score values for compounds **3 l** and **11** and its components.

Compound	GlideScore (Kcal/mol)	Lipophilic interactions (Kcal/mol)	Coulomb interactions (Kcal/mol)
**11**	−7.76	−2.62	−2.33
**3l**	−8.57	−2.82	−5.55

#### 2.3.3. Comparison of compounds 3 l and 3v: the detrimental effect of the oxygen atom on potency

**Figure 5.**
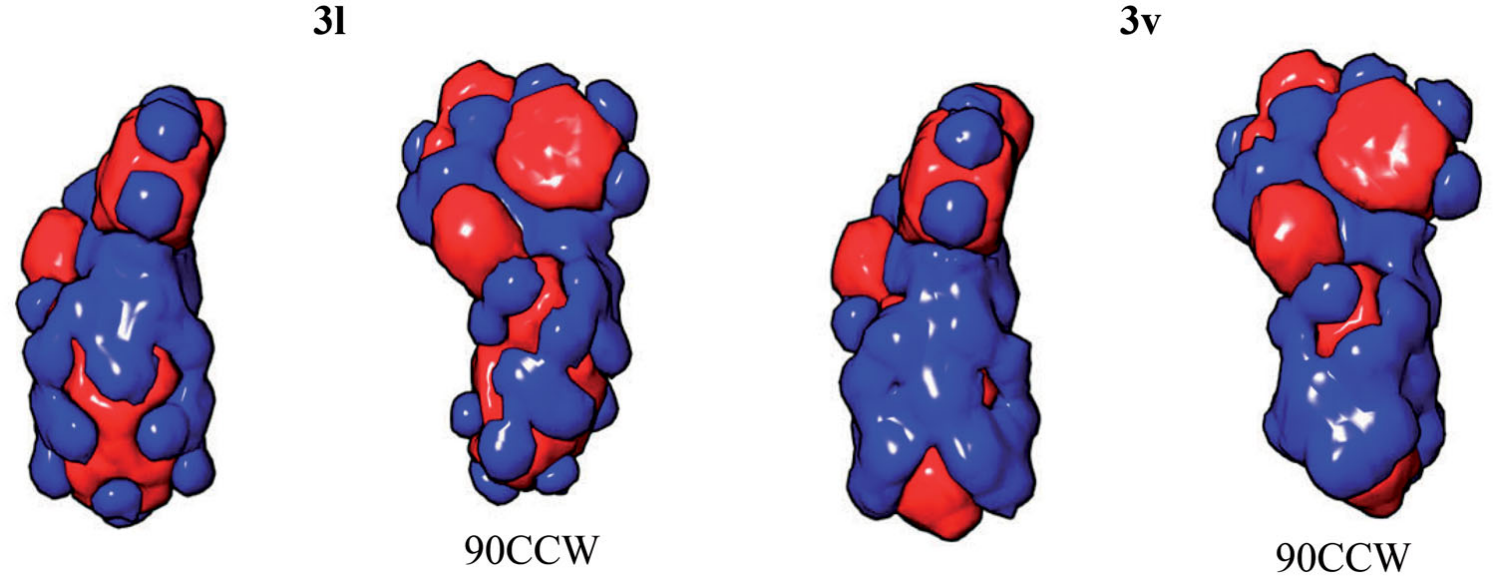
Electrostatic potential isosurfaces for compounds **3 l** (active) and **3v** (inactive); 90CCW – view of the ligand rotated 90° counter-clockwise.

In this case, we used MM-GBSA method for the analysis of the binding energy (ΔG) component. The per-atom ΔG value appeared to be the most informative parameter which provided a clear indication of the favorability of the ligand-protein complex formation and took into account the presence of the solvent as well as the ligand-induced effects on the protein conformation. As can be seen from the total ΔG values as well as per-atom energy components calculated for compounds **3l** and **3v** (Table 4) the morpholine oxygen atom appears to increase the ligand strain. Introduction of the oxygen atom into the distal six-membered ring (**3 l** → **3v**) induces a conformational rearrangement of the ligand in the enzyme’s binding site, likely due to the changes in the electrostatic profile (Figure 5).

**Table 4.** Per-atom free energy (ΔG) distribution on PARP1 ligands **3 l** and **3v** (**red**, positive values – unfavourable for binding; **green**, negative values – favourable for binding).

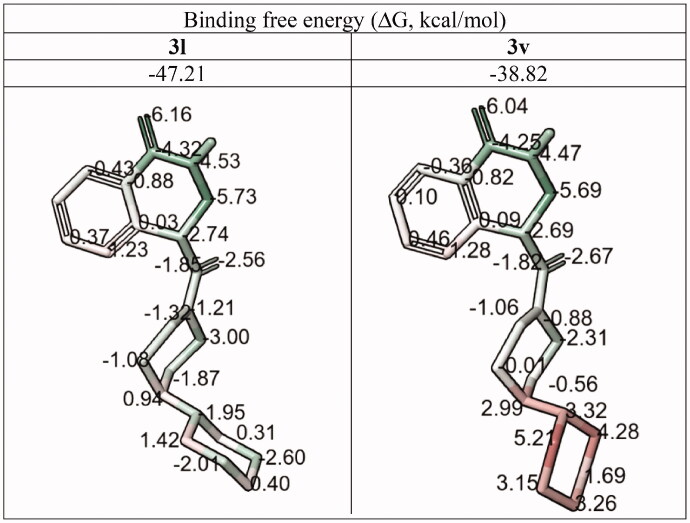	

#### 2.3.4. Comparison of compounds 3 l and 3 y: active vs. inactive isostere

**Figure 6.**
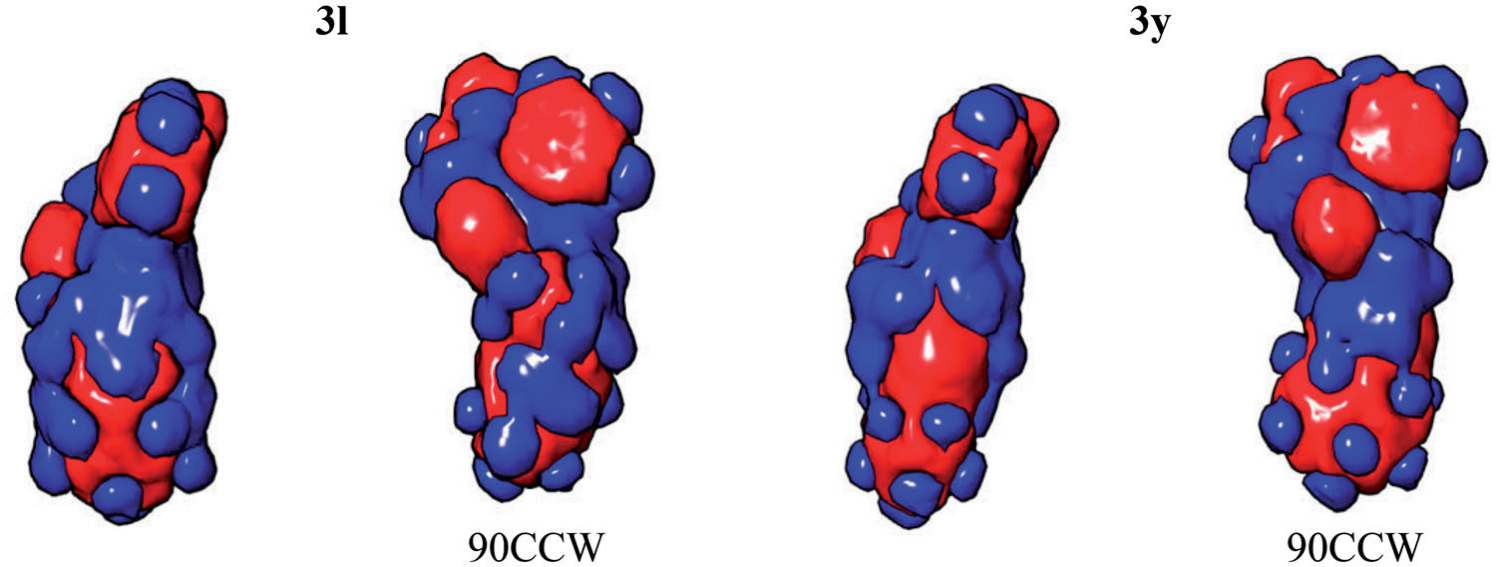
Electrostatic potential isosurfaces for compounds **3 l** (active) and **3 y** (inactive); 90CCW – view of the ligand rotated 90° counter-clockwise.

As in the previous case, changing the position of the nitrogen atom in the amide residue (**3l** → **3y**, 1,4′-bipiperidine → 4-(cyclohexyl)piperazine) alters the configuration on the molecular surface of the ligand and induces the increase in the strain energy and leads to the loss of the ligand-protein affinity. This is clearly demonstrated by the comparison of the per-atom free energy distribution (Table 5) and electrostatic potential isosurfaces (Figure 6) for the two ligands.

**Table 5.** Per-atom free energy (ΔG) distribution on PARP1 ligands **3l** and **3y** (**red**, positive values – unfavourable for binding; **green**, negative values – favourable for binding).

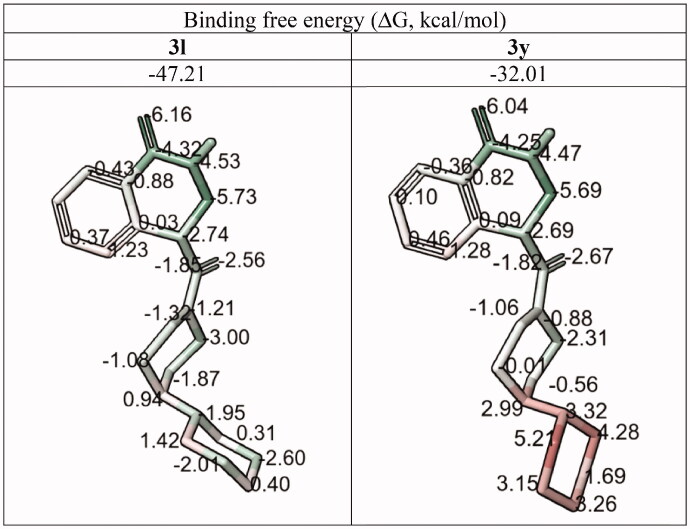	

### 2.4. Druglikeness and ADME parameters

Frontrunner compound **3aa** as well as its non-fluorinated analog **3 l** were assay for aqueous solubility in pH 7.4 phosphate buffer solution, stability in the presence of hepatic S9 fraction, liver microsomes and plasma, as well as for plasma protein binding in comparison with the approved PARP1 inhibitor Olaparib. Additionally, the molecular characteristics such as molecular weight, calculated octanol-water partitioning coefficient (cLogP) as well as hydrogen bond donor (HBD) and acceptor (HBA) count were compared for the two 1-oxo-3,4-dihydroisoquinoline-4-carboxamides and Olaparib (Table 6).

**Table 6.** Experimentally determined ADME parameters and calculated molecular characteristics for frontrunner compounds **3aa** and **3 l** as well as clinically used drug Olaparib.

Compound	Solubility^*a*^	Stability			PPB*^e^*	Calculated parameters^16^			
		S9*^b^*	HLM*^c^*	Plasma*^d^*		MW	cLogP	HBD	HBA
Olaparib	>100	1.1	15	100	90.2	434.5	2.52	1	7
**3aa**	>100	0.1	3.4	100	71.0	359.4	1.73	1	5
**3l**	>100	0.3	2.6	92.6	66.8	341.5	1.59	1	5

*^a^*Solubility (µM) in pH 7.4 0.01 M phosphate buffer solution; *^b^*Stability in the presence of hepatic S9 fraction (µL/min/mg); *^c^*Stability in the presence of human liver microsomes (µL/min/mg); *^d^*% compound remaining after incubation (4 h) with human plasma; *^e^*plasma protein binding (% bound).

While, of course, the approved PARP1 inhibitor Olaparib is well within the limits of druglikeness as defined by Lipinsky^17^ (and so are compounds **3aa** and **3 l**), the molecular weight advantage of about 90 Da carried by the 1-oxo-3,4-dihydroisoquinoline-4-carboxamides is evident. Also, the two compounds presented in this work are distinctly more hydrophilic which manifests itself^18^ in the markedly lower (by about 20% unbound) plasma protein binding. Quite reassuringly, the frontrunner PARP1 inhibitors developed in this study displayed similar stability in plasma to that of Olaparib. However, their metabolic stability, particularly in the presence of human liver microsomes is significantly higher.

## 3. Conclusion

We have developed a novel series of inhibitors of poly(ADP-ribose) polymerase (PARP) isoforms 1 and 2. Similarly to the marketed PARP1 inhibitor – anticancer drug Olaparib – the best compounds in the series displayed no pronounced selectivity between the two isoforms and some level of selectivity towards PARP2. The analysis of the structure-activity relationships and iterative inhibitors design allowed developing a lead compound whose potency is in the nanomolar range (PARP1/2 IC_50_ 63.1/29/4 *vs.* 2.8/0/7 for Olaparib). However, the frontrunner 1-oxo-3,4-dihydroisoquinoline-4-carboxamides are characterised by significantly lower molecular weight and lipophilicity as well as higher metabolic stability and free plasma concentration, compared to Olaparib. These findings clearly warrant further preclinical development of the lead inhibitor, 4-([1,4′-bipiperidine]-1′-carbonyl)-7-fluoro-3,4-dihydroisoquinolin-1(2*H*)-one, as the novel PARP1/2 inhibitor for the treatment of cancer.

## 4. Experimental section

### 4.1. General experimental

NMR spectra were recorded on a Bruker Avance III 400 spectrometer (^1^H: 400.13 MHz; ^13^С: 100.61 MHz; chemical shifts are reported as parts per million (δ, ppm); the residual solvent peaks were used as internal standards: 7.28 ^1^H in CDCl_3_, 77.02 ppm for ^13 ^C in CDCl_3_; multiplicities are abbreviated as follows: s = singlet, d = doublet, t = triplet, q = quartette, m = multiplet, br = broad; coupling constants, *J*, are reported in Hz. Mass spectra were recorded on a Bruker microTOF spectrometer (ESI ionization). Melting points were determined in open capillary tubes on Stuart SMP30 Melting Point Apparatus.

### 4.2. Synthetic organic chemistry

#### 4.2.1. General procedure for the preparation of amides 3a-aj, 11

A mixture of corresponding acid (0.50 mmol) and HATU (0.55 mmol, 209 mg) in dry DMF (1 ml) was stirred at room temperature for 15 min. After that amine (0.55 mmol) and triethylamine (0.55 mmol, 56 mg) were added. If amine was in the form of a hydrochloride salt, an additional equivalent of triethylamine was added. The mixture was stirred at room temperature for 16 h. Solvent was evaporated under reduced pressure. Chloroform (6 ml) and saturated sodium bicarbonate solution (3 ml) were added to residue. A mixture was thoroughly shaken, organic layer was separated. Aqueous phase was additionally extracted with chloroform (3 ml). Organic phases were combined, dried over Na_2_SO_4_ and the solvent was evaporated. The crude amide product was purified by column chromatography on silica gel using ethyl acetate-methanol-triethylamine 25:1:1 as eluent.

##### 4.2.1.1. Ethyl 4–(1-oxo-1,2,3,4-tetrahydroisoquinoline-4-carbonyl)piperazine-1-carboxylate (3a)

Yield 58 mg (35%); Yellow viscous oil; ^1^H NMR (400 MHz, DMSO-*d*_6_) *δ* 7.93–7.87 (m, 1H), 7.88 (dd, *J* = 7.7, 1.4 Hz, 1H), 7.49 (td, *J* = 7.5, 1.5 Hz, 1H), 7.40 (td, *J* = 7.5, 0.8 Hz, 1H), 7.15 (d, *J* = 7.5 Hz, 1H), 4.49–4.43 (m, 1H), 4.08 (q, *J* = 7.1 Hz, 2H), 3.68–3.60 (m, 2H), 3.60–3.53 (m, 2H), 3.53–3.42 (m, 5H), 3.42–3.35 (m, 1H), 1.21 (t, *J* = 7.1 Hz, 3H); ^13^C NMR (101 MHz, DMSO-*d*_6_) *δ* 169.5, 164.5, 155.1, 138.5, 132.2, 130.5, 127.72, 127.66, 126.8, 61.4, 45.5, 44.1, 43.7, 42.7, 41.6, 15.0; HRMS (ESI+), *m/z* calcd for C_17_H_21_N_3_NaO_4_ [M + Na]^+^ 354.1424, found 354.1409.

##### 4.2.1.2. 4–(4-Methylpiperidine-1-carbonyl)-3,4-dihydroisoquinolin-1(2H)-one (3b)

Yield 89 mg (65%); Light yellow viscous oil; ^1^H NMR (400 MHz, CDCl_3_) *δ* 8.10 (d, *J* = 7.5 Hz, 1H), 7.53–7.44 (m, 1H), 7.44–7.37 (m, 1H), 7.18–7.01 (m, 2H), 4.73 (br.d, *J* = 12.5 Hz, 1H), 4.39–4.31 (m, 1H), 3.99–3.84 (m, 2H), 3.59–3.49 (m, 1H), 3.16 (t, *J* = 12.6 Hz, 1H), 2.83–2.65 (m, 1H), 1.85–1.63 (m, 3H), 1.30–1.11 (m, 2H), 1.07–0.96 (m, 3H); ^13^C NMR (101 MHz, CDCl_3_) *δ* 168.7, 165.9, 137.3, 132.5, 129.3, 128.4, 127.8, 125.2, 46.8, 46.1, 43.1, 34.9, 34.0, 31.3, 30.9, 21.7; HRMS (ESI+), *m/z* calcd for C_16_H_20_N_2_NaO_2_ [M + Na]^+^ 295.1417, found 295.1419.

##### 4.2.1.3. N-Benzyl-N-methyl-1-oxo-1,2,3,4-tetrahydroisoquinoline-4-carboxamide (3c)

Yield 84 mg (57%); Beige solid; m. p. = 148–149 °C; ^1^H NMR (400 MHz, CDCl_3_) mixture of rotamers **A** and **B** 3:2, *δ* 8.14–8.08 (m, 1H **A**, 1H **B**), 7.57–7.30 (m, 8H **A**, 5H **B**), 7.20 (d, *J* = 7.3 Hz, 2H **B**), 7.11 (d, *J* = 7.5 Hz, 1H **B**), 7.04 (d, *J* = 6.6 Hz, 1H **A**, 1H **B**), 4.86–4.77 (m, 1H **A**, 1H **B**), 4.67 (d, *J* = 14.3 Hz, 1H **A**), 4.54 (d, *J* = 17.1 Hz, 1H **B**), 4.38 (dd, *J* = 11.4, 4.9 Hz, 1H **A**), 4.31 (dd, *J* = 11.0, 4.9 Hz, 1H **B**), 4.04–3.92 (m, 1H **A**, 1H **B**), 3.58 (dt, *J* = 12.2, 4.8 Hz, 1H **A**), 3.48 (dt, *J* = 12.0, 4.8 Hz, 1H **B**), 3.17 (s, 3H), 3.08 (s, 3H); ^13^C NMR (101 MHz, CDCl_3_) mixture of rotamers **A** and **B** *δ* 171.0, 170.5, 166.0, 165.9, 137.2, 137.1, 137.0, 136.1, 132.7, 129.2, 129.1, 128.8, 128.6, 128.5, 128.4, 128.22, 128.13, 128.04, 127.97, 127.92, 127.82, 126.0, 125.2, 125.0, 53.7, 51.3, 43.2, 43.0, 41.4, 35.1, 34.7; HRMS (ESI+), *m/z* calcd for C_18_H_19_N_2_O_2_ [M + H]^+^ 295.1441, found 295.1448.

##### 4.2.1.4. N-(4-Chlorobenzyl)-1-oxo-1,2,3,4-tetrahydroisoquinoline-4-carboxamide (3d)

Yield 83 mg (53%); Light yellow viscous oil; ^1^H NMR (400 MHz, DMSO-*d*_6_) *δ* 8.51 (t, *J* = 5.9 Hz, 1H), 7.91 (br.s, 1H), 7.88 (dd, *J* = 7.7, 1.3 Hz, 1H), 7.51 (td, *J* = 7.5, 1.5 Hz, 1H), 7.42 (dd, *J* = 7.6, 1.0 Hz, 1H), 7.40–7.37 (m, 2H), 7.31–7.25 (m, 3H), 4.31 (dd, *J* = 5.8, 1.6 Hz, 2H), 3.89 (t, *J* = 5.6 Hz, 1H), 3.67–3.52 (m, 2H); ^13^C NMR (126 MHz, DMSO-*d*_6_) *δ* 171.6, 165.2, 138.4, 137.7, 132.9, 131.9, 129.3, 129.2, 128.7, 128.3, 127.9, 127.7, 44.2, 42.6, 42.2; HRMS (ESI+), *m/z* calcd for C_17_H_15_ClN_2_NaO_2_ [M + Na]^+^ 337.0714, found 337.0715.

##### 4.2.1.5. 4–(1,2,3,4-Tetrahydroisoquinoline-2-carbonyl)-3,4-dihydroisoquinolin-1(2H)-one (3e)

Yield 110 mg (72%); Beige solid; m. p. = 222–223 °C; ^1^H NMR (400 MHz, DMSO-*d*_6_) *δ* 7.94–7.86 (m, 2H), 7.52–7.36 (m, 2H), 7.25–7.17 (m, 4H), 7.18–7.03 (m, 1H), 4.86 (br.s, 1H), 4.82–4.61 (m, 1H), 4.61–4.52 (m, 1H), 3.96–3.64 (m, 2H), 3.61–3.45 (m. 2H), 3.00–2.81 (m, 2H); ^13^C NMR (101 MHz, DMSO-*d*_6_) *δ* 169.8, 164.5, 138.6, 134.9, 133.9, 132.1, 130.6, 129.1, 128.9, 127.7, 127.7, 127.0, 126.9, 126.7, 47.2, 44.5, 43.5, 42.7, 29.6; HRMS (ESI+): *m/z* calcd for C_19_H_19_N_2_O_2_ [M + H]^+^ 307.1441, found 307.1435.

##### 4.2.1.6. 4–(4-ethylpiperazine-1-carbonyl)-3,4-dihydroisoquinolin-1(2H)-one (3f)

Yield 88 mg (61%); Dark orange viscous oil; ^1^H NMR (400 MHz, CDCl_3_) *δ* 8.11 (dd, *J* = 7.6, 1.3 Hz, 1H), 7.50 (td, *J* = 7.5, 1.4 Hz, 1H), 7.42 (t, *J* = 7.5 Hz, 1H), 7.11 (d, *J* = 7.6 Hz, 1H), 6.87 (br.s, 1H), 4.34 (dd, *J* = 11.2, 5.0 Hz, 1H), 3.93 (ddd, *J* = 12.5, 11.4, 1.1 Hz, 1H), 3.92–3.83 (m, 1H), 3.80–3.71 (m, 1H), 3.66–3.58 (m, 2H), 3.54 (dt, *J* = 12.5, 5.0 Hz, 1H), 2.60–2.48 (m, 4H), 2.50 (q, *J* = 7.2 Hz, 2H), 1.13 (t, *J* = 7.2 Hz, 3H); ^13^C NMR (101 MHz, CDCl_3_) *δ* 168.8, 165.7, 137.0, 132.5, 129.2, 128.5, 127.9, 125.1, 53.2, 52.5, 52.2, 46.0, 43.0, 42.0, 41.2, 11.9; HRMS (ESI+), *m/z* calcd for C_16_H_22_N_3_O_2_ [M + H]^+^ 288.1707, found 288.1709.

##### 4.2.1.7. Ethyl 1–(1-oxo-1,2,3,4-tetrahydroisoquinoline-4-carbonyl)piperidine-4-carboxylate (3 g)

Yield 71 mg (43%); White solid; m. p. = 120–121 °C; ^1^H NMR (400 MHz, CDCl_3_) *δ* 8.11 (dd, *J* = 7.6, 1.1 Hz, 1H), 7.55–7.46 (m, 1H), 7.42 (t, *J* = 7.5 Hz, 1H), 7.15–7.00 (m, 1H), 6.79 (br.s, 1H), 4.64–4.46 (m, 1H), 4.35 (dd, *J* = 11.2, 4.9 Hz, 1H), 4.19 (q, *J* = 6.7 Hz, 2H), 3.93 (t, *J* = 11.5 Hz, 2H), 3.54 (dt, *J* = 12.0, 4.6 Hz, 1H), 3.29 (br.q, *J* = 10.0 Hz, 1H), 3.04 (br.s, 1H), 2.63 (br.s, 1H), 2.11–1.97 (m, 2H), 1.86–1.67 (m, 2H), 1.29 (t, *J* = 7.1 Hz, 3H); ^13^C NMR (101 MHz, CDCl_3_) *δ* 173.9, 168.7, 165.7, 137.0, 132.5, 129.2, 128.6, 128.0, 125.0, 60.8, 45.6, 45.1, 43.1, 41.3, 40.6, 28.8, 28.1, 14.2; HRMS (ESI+), *m/z* calcd for C_18_H_23_N_2_O_4_ [M + H]^+^ 331.1652, found 331.1656.

##### 4.2.1.8. 4–(4-Methylpiperazine-1-carbonyl)-3,4-dihydroisoquinolin-1(2H)-one (3 h)

Yield 79 mg (58%); Beige solid; m. p. = 163–164 °C; ^1^H NMR (400 MHz, CDCl_3_) *δ* 8.12 (dd, *J* = 7.6, 1.2 Hz, 1H), 7.51 (td, *J* = 7.5, 1.4 Hz, 1H), 7.43 (t, *J* = 7.5 Hz, 1H), 7.12 (d, *J* = 7.6 Hz, 1H), 6.64 (br.d, *J* = 3.7 Hz, 1H), 4.34 (dd, *J* = 11.3, 5.0 Hz, 1H), 3.94 (ddd, *J* = 12.4, 11.3, 1.1 Hz, 1H), 3.93–3.70 (m, 2H), 3.61 (br.s, 2H), 3.54 (dt, *J* = 12.4, 4.9 Hz, 1H), 2.50 (br.s, 4H), 2.37 (s, 3H); ^13^C NMR (101 MHz, CDCl_3_) *δ* 168.8, 165.6, 136.9, 132.5, 129.2, 128.6, 128.0, 125.1, 55.4, 54.9, 46.0, 43.1, 41.9, 41.2; HRMS (ESI+), *m/z* calcd for C_15_H_20_N_3_O_2_ [M + H]^+^ 274.1550, found 274.1561.

##### 4.2.1.9. 4–(4-Tosylpiperazine-1-carbonyl)-3,4-dihydroisoquinolin-1(2H)-one (3i)

Yield 151 mg (73%); Beige solid; m. p. = 228–229 °C; ^1^H NMR (400 MHz, DMSO-*d*_6_) *δ* 7.86–7.82 (m, 2H), 7.66 (d, *J* = 8.3 Hz, 2H), 7.49 (d, *J* = 8.1 Hz, 2H), 7.42 (td, *J* = 7.4, 1.7 Hz, 1H), 7.38 (td, *J* = 7.5, 1.1 Hz, 1H), 7.05 (d, *J* = 7.3 Hz, 1H), 4.38 (t, *J* = 6.2 Hz, 1H), 3.77–3.63 (m, 3H), 3.44–3.39 (m, 2H), 3.21–3.07 (m, 1H), 3.07–2.97 (m, 2H), 2.97–2.80 (m, 2H), 2.43 (s, 3H); ^13^C NMR (101 MHz, DMSO-*d*_6_) *δ* 169.5, 164.5, 144.4, 138.3, 132.6, 132.0, 130.5, 130.5, 128.1, 127.7, 127.6, 126.8, 46.6, 46.3, 43.5, 43.3, 42.7, 42.6, 21.5; HRMS (ESI+), *m/z* calcd for C_21_H_24_N_3_O_4_S [M + H]^+^ 414.1482, found 414.1495.

##### 4.2.1.10. 4-(Morpholine-4-carbonyl)-3,4-dihydroisoquinolin-1(2H)-one (3j)

Yield 70 mg (54%); Yellow viscous oil; ^1^H NMR (400 MHz, CDCl_3_) *δ* 8.13 (d, *J* = 7.6 Hz, 1H), 7.53 (t, *J* = 7.0 Hz, 1H), 7.45 (t, *J* = 7.5 Hz, 1H), 7.13 (d, *J* = 7.5 Hz, 1H), 6.70 (br.s, 1H), 4.37–4.27 (m, 1H), 4.00–3.84 (m, 2H), 3.84–3.75 (m, 4H), 3.75–3.66 (m, 1H), 3.66–3.51 (m, 3H); ^13^C NMR (101 MHz, CDCl_3_) *δ* 169.0, 165.6, 136.8, 132.6, 129.2, 128.7, 128.1, 124.98, 67.0, 66.8, 46.4, 43.0, 42.3, 41.1; HRMS (ESI+), *m/z* calcd for C_14_H_17_N_2_O_3_ [M + H]^+^ 261.1234, found 261.1241.

##### 4.2.1.11. 4–(4-Benzylpiperidine-1-carbonyl)-3,4-dihydroisoquinolin-1(2H)-one (3k)

Yield 117 mg (67%); Beige solid; m. p. = 191–192 °C; ^1^H NMR (400 MHz, CDCl_3_) *δ* 8.12 (d, *J* = 7.1 Hz, 1H), 7.55–7.38 (m, 2H), 7.35–7.29 (m, 2H), 7.27–7.20 (m, 1H), 7.20–7.00 (m, 3H), 6.95 (d, *J* = 16.7 Hz, 1H), 4.77 (d, *J* = 12.5 Hz, 1H), 4.35 (dd, *J* = 11.3, 4.7 Hz, 1H), 4.00–3.84 (m, 2H), 3.59–3.49 (m, 1H), 3.13 (t, *J* = 12.7 Hz, 1H), 2.78–2.57 (m, 3H), 1.93–1.73 (m, 3H), 1.38–1.18 (m, 2H); ^13^C NMR (101 MHz, CDCl_3_) *δ* 168.7, 165.9, 139.7, 137.4, 137.2, 132.5, 129.3, 129.1, 128.5, 128.4, 127.8, 126.2, 125.2, 46.7, 46.1, 43.1, 42.9, 42.8, 42.6, 42.3, 41.4, 41.3, 38.4, 38.0, 32.9, 32.0; HRMS (ESI+), *m/z* calcd for C_22_H_25_N_2_O_2_ [M + H]^+^ 349.1911, found 349.1914.

##### 4.2.1.12. 4-([1,4'-Bipiperidine]-1'-carbonyl)-3,4-dihydroisoquinolin-1(2H)-one (3 l)

Yield 99 mg (58%); Dark red viscous oil; ^1^H NMR (400 MHz, CDCl_3_) *δ* 8.12 (d, *J* = 7.1 Hz, 1H), 7.54–7.46 (m, 1H), 7.43 (t, *J* = 7.4 Hz, 1H), 7.19–7.00 (m, 1H), 6.42 (br.s, 1H), 4.83 (br.d, *J* = 10.4 Hz, 1H), 4.39–4.31 (m, 1H), 4.04–3.88 (m, 2H), 3.53 (dt, *J* = 12.0, 4.7 Hz, 1H), 3.19 (t, *J* = 11.4 Hz, 1H), 2.82–2.48 (m, 6H), 2.09–1.96 (m, 2H), 1.74–1.63 (m, 4H), 1.59 (qd, *J* = 11.9, 3.5 Hz, 2H), 1.53–1.45 (m, 2H); ^13^C NMR (101 MHz, CDCl_3_) *δ* 168.5, 165.6, 137.0, 132.5, 129.2, 128.6, 127.9, 125.1, 62.6, 62.3, 50.5, 50.3, 45.9, 45.3, 43.2, 41.7, 41.3, 28.9, 25.9, 24.4; HRMS (ESI+), *m/z* calcd for C_20_H_28_N_3_O_2_ [M + H]^+^ 342.2176, found 342.2180.

##### 4.2.1.13. 4-(Pyrrolidine-1-carbonyl)-3,4-dihydroisoquinolin-1(2H)-one (3 m)

Yield 75 mg (61%); Yellow viscous oil; ^1^H NMR (400 MHz, CDCl_3_) *δ* 8.12 (dd, *J* = 7.6, 1.2 Hz, 1H), 7.49 (td, *J* = 7.5, 1.4 Hz, 1H), 7.42 (t, *J* = 7.5 Hz, 1H), 7.05 (d, *J* = 7.6 Hz, 1H), 6.62 (br.s, 1H), 4.23 (dd, *J* = 11.7, 5.1 Hz, 1H), 3.96 (ddd, *J* = 12.5, 11.7, 0.8 Hz, 1H), 3.72–3.62 (m, 2H), 3.62–3.53 (m, 3H), 2.11–1.92 (m, 4H); ^13^C NMR (101 MHz, CDCl_3_) *δ* 168.9, 165.7, 137.0, 132.6, 129.2, 128.6, 127.8, 125.0, 46.9, 46.0, 43.5, 42.7, 26.2, 24.4; HRMS (ESI+), *m/z* calcd for C_14_H_17_N_2_O_2_ [M + H]^+^ 245.1285, found 245.1291.

##### 4.2.1.14. 4-(Azepane-1-carbonyl)-3,4-dihydroisoquinolin-1(2H)-one (3n)

Yield 78 mg (57%); Yellow viscous oil; ^1^H NMR (400 MHz, CDCl_3_) *δ* 8.11 (dd, *J* = 7.6, 1.1 Hz, 1H), 7.49 (td, *J* = 7.5, 1.3 Hz, 1H), 7.41 (t, *J* = 7.5 Hz, 1H), 7.09 (d, *J* = 7.6 Hz, 1H), 6.93 (d, *J* = 3.7 Hz, 1H), 4.33 (dd, *J* = 11.8, 5.1 Hz, 1H), 4.02–3.90 (m, 2H), 3.67 (dt, *J* = 14.6, 4.9 Hz, 1H), 3.57–3.40 (m, 3H), 1.95–1.53 (m, 8H); ^13^C NMR (101 MHz, CDCl_3_) *δ* 170.1, 165.9, 137.5, 132.6, 129.2, 128.4, 127.8, 125.2, 48.3, 46.3, 43.3, 41.6, 29.7, 27.7, 27.0, 26.8; HRMS (ESI+), *m/z* calcd for C_16_H_21_N_2_O_2_ [M + H]^+^ 273.1598, found 273.1599.

##### 4.2.1.15. 4-(Thiomorpholine-4-carbonyl)-3,4-dihydroisoquinolin-1(2H)-one (3o)

Yield 68 mg (49%); Beige solid; m. p. = 171–172 °C; ^1^H NMR (400 MHz, CDCl_3_) *δ* 8.12 (d, *J* = 7.6 Hz, 1H), 7.51 (td, *J* = 7.5, 1.2 Hz, 1H), 7.44 (t, *J* = 7.5 Hz, 1H), 7.08 (d, *J* = 7.6 Hz, 1H), 6.93 (br.s, 1H), 4.32 (dd, *J* = 11.1, 4.9 Hz, 1H), 4.23 (br.s, 1H), 3.99–3.74 (m, 4H), 3.55 (dt, *J* = 12.4, 4.9 Hz, 1H), 2.83–2.60 (m, 4H); ^13^C NMR (101 MHz, CDCl_3_) *δ* 168.9, 165.7, 136.7, 132.6, 129.3, 128.6, 128.1, 125.0, 48.8, 44.7, 43.1, 41.3, 28.3, 27.7; HRMS (ESI+), *m/z* calcd for C_14_H_17_N_2_O_2_S [M + H]^+^ 277.1005, found 277.1003.

##### 4.2.1.16. N-(4-Fluorobenzyl)-1-oxo-1,2,3,4-tetrahydroisoquinoline-4-carboxamide (3p)

Yield 67 mg (45%); White solid; m. p. = 188–189 °C; ^1^H NMR (400 MHz, DMSO-*d*_6_) *δ* 8.50 (t, *J* = 5.7 Hz, 1H), 7.91 (br.s, 1H), 7.88 (d, *J* = 7.5 Hz, 1H), 7.51 (td, *J* = 7.4, 1.1 Hz, 1H), 7.41 (t, *J* = 7.3 Hz, 1H), 7.34–7.24 (m, 3H), 7.15 (t, *J* = 8.8 Hz, 2H), 4.31 (d, *J* = 5.7 Hz, 2H), 3.89 (t, *J* = 5.6 Hz, 1H), 3.67–3.51 (m, 2H); ^13^C NMR (101 MHz, DMSO-*d*_6_) *δ* 170.9, 164.3, 161.7 (d, *J* = 242.1 Hz), 138.2, 136.0 (d, *J* = 3.0 Hz), 132.2, 130.0, 129.7 (d, *J* = 7.9 Hz), 127.9, 127.7, 127.6, 115.5 (d, *J* = 21.4 Hz), 44.4, 42.7, 42.1; HRMS (ESI+), *m/z* calcd for C_17_H_16_FN_2_O_2_ [M + H]^+^ 299.1190, found 299.1183.

##### 4.2.1.17. 4–(4-(2-Methoxyphenyl)piperazine-1-carbonyl)-3,4-dihydroisoquinolin-1(2H)-one (3q)

Yield 104 mg (57%); Light yellow solid; m. p. = 155–156 °C; ^1^H NMR (400 MHz, CDCl_3_) *δ* 8.14 (dd, *J* = 7.6, 0.9 Hz, 1H), 7.52 (td, *J* = 7.5, 1.2 Hz, 1H), 7.45 (t, *J* = 7.5 Hz, 1H), 7.18 (d, *J* = 7.6 Hz, 1H), 7.13–7.06 (m, 1H), 7.00–6.95 (m, 2H), 6.93 (d, *J* = 8.1 Hz, 1H), 6.51 (d, *J* = 4.1 Hz, 1H), 4.41 (dd, *J* = 11.4, 5.0 Hz, 1H), 4.10–3.94 (m, 3H), 3.91 (s, 3H), 3.78 (br.s, 2H), 3.58 (dt, *J* = 12.3, 4.9 Hz, 1H), 3.21–3.12 (m, 4H); ^13^C NMR (101 MHz, CDCl_3_) *δ* 168.9, 165.6, 152.3, 140.4, 137.0, 132.6, 129.2, 128.7, 128.0, 125.1, 123.8, 121.1, 118.5, 111.5, 55.5, 51.3, 50.7, 46.4, 43.1, 42.3, 41.3; HRMS (ESI+), *m/z* calcd for C_21_H_24_N_3_O_3_ [M + H]^+^ 366.1812, found 366.1815.

##### 4.2.1.18. 4–(1-Oxa-9-azaspiro[5.5]undecane-9-carbonyl)-3,4-dihydroisoquinolin-1(2H)-one (3r)

Yield 80 mg (49%); Light yellow viscous oil; ^1^H NMR (400 MHz, CDCl_3_) *δ* 8.16–8.10 (m, 1H), 7.50 (t, *J* = 7.4 Hz, 1H), 7.43 (t, *J* = 7.5 Hz, 1H), 7.16–7.04 (m, 1H), 6.48–6.36 (m, 1H), 4.49–4.41 (m, 1H), 4.41–4.32 (m, 1H), 3.96 (q, *J* = 12.6 Hz, 1H), 3.75–3.48 (m, 5H), 3.27–3.10 (m, 1H), 2.10 (t, *J* = 13.3 Hz, 1H), 1.99 (d, *J* = 13.6 Hz, 1H), 1.74–1.41 (m, 8H); ^13^C NMR (101 MHz, CDCl_3_) *δ* 168.5, 165.6, 137.1, 132.4, 129.1, 128.5, 127.9, 125.0, 69.9, 61.1, 43.2, 42.0, 41.4, 37.7, 35.9, 35.8, 35.4, 33.5, 26.0; HRMS (ESI+), *m/z* calcd for C_19_H_24_N_2_NaO_3_ [M + Na]^+^ 351.1679, found 351.1692.

##### 4.2.1.19. 1-oxo-N-(4-(piperidin-1-yl)benzyl)-1,2,3,4-tetrahydroisoquinoline-4-carboxamide (3 s)

Yield 100 mg (55%); Beige solid; m. p. = 235–236 °C; ^1^H NMR (400 MHz, DMSO-*d*_6_) *δ* 8.41 (t, *J* = 5.6 Hz, 1H), 7.90 (br.s, 1H), 7.88 (dd, *J* = 7.7, 1.0 Hz, 1H), 7.50 (td, *J* = 7.5, 1.3 Hz, 1H), 7.40 (t, *J* = 7.2 Hz, 1H), 7.25 (d, *J* = 7.5 Hz, 1H), 7.11 (d, *J* = 8.6 Hz, 2H), 6.88 (d, *J* = 8.6 Hz, 2H), 4.22 (d, *J* = 5.7 Hz, 2H), 3.88 (t, *J* = 5.8 Hz, 1H), 3.66–3.48 (m, 2H), 3.13–3.06 (m, 4H), 1.65–1.57 (m, 4H), 1.57–1.49 (m, 2H); ^13^C NMR (101 MHz, DMSO-*d*_6_) *δ* 170.67, 164.33, 151.22, 138.39, 132.18, 130.01, 129.40, 128.61, 127.80, 127.62, 127.60, 116.27, 50.26, 44.43, 42.70, 42.39, 25.67, 24.39; HRMS (ESI+), *m/z* calcd for C_22_H_25_N_3_NaO_2_ [M + Na]^+^ 386.1839, found 386.1829.

##### 4.2.1.20. 4–(4–(4-Fluorobenzyl)piperazine-1-carbonyl)-3,4-dihydroisoquinolin-1(2H)-one (3t)

This compound was purified with HPLC using MeCN–H_2_O as eluent with addition of CF_3_CO_2_H and isolated as trifluoroacetate salt. Yield 91 mg (38%); Dark brown viscous oil; ^1^H NMR (400 MHz, DMSO-*d*_6_) *δ* 7.93 (br.s, 1H), 7.89 (dd, *J* = 7.6, 1.2 Hz, 1H), 7.60–7.53 (m, 2H), 7.51 (td, *J* = 7.5, 1.1 Hz, 1H), 7.42 (t, *J* = 7.4 Hz, 1H), 7.34 (t, *J* = 8.6 Hz, 2H), 7.16 (br.s, 1H), 4.49 (t, *J* = 6.3 Hz, 1H), 4.34 (br.s, 4H), 3.49 (br.s, 4H), 3.13 (br.s, 4H); ^13^C NMR (101 MHz, DMSO-*d*_6_) *δ* 169.6, 164.5, 163.22 (d, *J* = 251.0 Hz), 138.2, 134.0, 132.2, 130.5, 127.8 (d, *J* = 12.4 Hz), 126.7, 116.3 (d, *J* = 21.6 Hz), 79.7, 58.7, 51.6, 51.0, 42.6; HRMS (ESI+), *m/z* calcd for C_21_H_22_FN_3_NaO_2_ [M + Na]^+^ 390.1588, found 390.1596.

##### 4.2.1.21. 4–(4-(2-Fluorobenzyl)piperazine-1-carbonyl)-3,4-dihydroisoquinolin-1(2H)-one (3 u)

This compound was purified with HPLC using MeCN–H_2_O as eluent with addition of CF_3_CO_2_H and isolated as trifluoroacetate salt. Yield 84 mg (35%); Brown viscous oil; ^1^H NMR (400 MHz, DMSO-*d*_6_) *δ* 7.89 (br.s, 2H), 7.62–7.08 (m, 7H), 4.47 (br.s, 1H), 4.25 (br.s, 2H), 3.48 (br.s, 4H), 3.11 (br.s, 4H); ^13^C NMR (101 MHz, DMSO-*d*_6_) *δ* 169.5, 164.6, 161.6 (d, *J* = 245.3 Hz), 138.3, 134.5, 133.8, 132.2, 131.0, 130.6, 127.8, 127.7, 126.8, 125.3, 116.2 (d, *J* = 21.8 Hz), 79.8, 53.2, 51.9, 51.5, 42.6; HRMS (ESI+), *m/z* calcd for C_21_H_23_FN_3_O_2_ [M + H]^+^ 368.1769, found 368.1782.

##### 4.2.1.22. 4–(4-Morpholinopiperidine-1-carbonyl)-3,4-dihydroisoquinolin-1(2H)-one (3v)

Yield 93 mg (54%); Light brown viscous oil; ^1^H NMR (400 MHz, CDCl_3_) *δ* 8.14 (d, *J* = 7.6 Hz, 1H), 7.51 (br.s, 1H), 7.44 (t, *J* = 7.4 Hz, 1H), 7.10 (m, 1H), 6.07 (br.s, 1H), 4.77 (br.s, 1H), 4.40–4.32 (m, 1H), 3.96 (t, *J* = 11.3 Hz, 2H), 3.77 (br.s, 4H), 3.53 (dt, *J* = 12.3, 4.9 Hz, 1H), 3.29–3.16 (m, 1H), 2.92–2.75 (m, 1H), 2.60 (br.s, 4H), 2.54–2.40 (m, 1H), 2.08–1.95 (m, 2H), 1.57–1.48 (m, 2H); ^13^C NMR (101 MHz, CDCl_3_) *δ* 168.4, 165.4, 137.0, 132.6, 129.1, 128.7, 128.0, 125.0, 67.1, 61.6, 50.1, 49.9, 45.4, 44.9, 43.2, 41.3, 41.0, 29.7, 28.4; HRMS (ESI+), *m/z* calcd for C_19_H_26_N_3_O_3_ [M + H]^+^ 344.1969, found 344.1979.

##### 4.2.1.23. 4-(Piperazine-1-carbonyl)-3,4-dihydroisoquinolin-1(2H)-one (3w)

This compound was purified with HPLC using MeCN–H_2_O as eluent with addition of CF_3_CO_2_H and isolated as trifluoroacetate salt. Yield 77 mg (41%); Light brown viscous oil; ^1^H NMR (400 MHz, DMSO-*d*_6_) *δ* 7.91 (br.s, 1H), 7.88 (dd, *J* = 7.6, 1.1 Hz, 1H), 7.50 (td, *J* = 7.5, 1.3 Hz, 1H), 7.41 (t, *J* = 7.3 Hz, 1H), 7.18 (d, *J* = 7.5 Hz, 1H), 4.48 (t, *J* = 6.3 Hz, 1H), 4.34 (br.s, 4H), 3.84 (br.s, 2H), 3.75 (br.s, 2H), 3.50 (dd, *J* = 6.1, 2.8 Hz, 1H), 3.26–3.05 (m, 5H); ^13^C NMR (101 MHz, DMSO-*d*_6_) *δ* 169.7, 164.5, 138.2, 132.2, 130.5, 127.8, 127.7, 126.8, 43.5, 43.2, 42.9, 42.6; HRMS (ESI+), *m/z* calcd for C_14_H_18_N_3_O_2_ [M + H]^+^ 260.1394, found 260.1396.

##### 4.2.1.24. 4–(4-Cyclopentylpiperazine-1-carbonyl)-3,4-dihydroisoquinolin-1(2H)-one (3x)

Yield 100 mg (61%); White solid; m. p. = 177–178 °C; ^1^H NMR (400 MHz, CDCl_3_) *δ* 8.13 (dd, *J* = 7.5, 1.1 Hz, 1H), 7.51 (td, *J* = 7.5, 1.3 Hz, 1H), 7.44 (t, *J* = 7.5 Hz, 1H), 7.13 (d, *J* = 7.6 Hz, 1H), 6.26 (br.s, 1H), 4.35 (dd, *J* = 11.4, 5.0 Hz, 1H), 3.96 (t, *J* = 12.0 Hz, 1H), 3.95–3.69 (m, 2H), 3.62 (br.s, 2H), 3.54 (dt, *J* = 12.3, 4.9 Hz, 1H), 2.68–2.51 (m, 5H), 1.96–1.85 (m, 2H), 1.79–1.68 (m, 2H), 1.66–1.55 (m, 2H), 1.50–1.40 (m, 2H); ^13^C NMR (101 MHz, CDCl_3_) *δ* 168.6, 165.5, 136.9, 132.5, 129.2, 128.7, 128.0, 125.0, 67.2, 52.7, 52.1, 46.0, 43.1, 42.0, 41.2, 30.4, 24.1; HRMS (ESI+), *m/z* calcd for C_19_H_26_N_3_O_2_ [M + H]^+^ 328.2020, found 328.2033.

##### 4.2.1.25. 4–(4-Cyclohexylpiperazine-1-carbonyl)-3,4-dihydroisoquinolin-1(2H)-one (3 y)

Yield 109 mg (64%); Light brown solid; m. p. = 197–198 °C; ^1^H NMR (400 MHz, CDCl_3_) *δ* 8.12 (d, *J* = 7.5 Hz, 1H), 7.51 (t, *J* = 7.5 Hz, 1H), 7.43 (t, *J* = 7.5 Hz, 1H), 7.12 (d, *J* = 7.6 Hz, 1H), 6.48 (br.s, 1H), 4.35 (dd, *J* = 11.4, 4.9 Hz, 1H), 3.95 (t, *J* = 11.9 Hz, 1H), 3.89–3.70 (m, 2H), 3.62–3.56 (m, 2H), 3.54 (dt, *J* = 12.4, 5.0 Hz, 1H), 2.66 (m, 4H), 2.39–2.31 (m, 1H), 1.91–1.79 (m, 4H), 1.70–1.62 (m, 1H), 1.31–1.21 (m, 4H), 1.18–1.10 (m, 1H); ^13^C NMR (101 MHz, CDCl_3_) *δ* 168.7, 165.6, 137.0, 132.5, 129.2, 128.6, 127.9, 125.1, 63.5, 49.6, 48.9, 46.6, 43.1, 42.5, 41.2, 28.9, 26.2, 25.8; HRMS (ESI+), *m/z* calcd for C_20_H_28_N_3_O_2_ [M + H]^+^ 342.2176, found 342.2178.

##### 4.2.1.26. 4–(4-Cycloheptylpiperazine-1-carbonyl)-3,4-dihydroisoquinolin-1(2H)-one (3z)

Yield 105 mg (59%); Light brown solid; m. p. = 207–208 °C; ^1^H NMR (400 MHz, CDCl_3_) *δ* 8.13 (dd, *J* = 7.6, 1.3 Hz, 1H), 7.52 (td, *J* = 7.5, 1.5 Hz, 1H), 7.44 (t, *J* = 7.5 Hz, 1H), 7.13 (d, *J* = 7.6 Hz, 1H), 6.17 (d, *J* = 4.0 Hz, 1H), 4.35 (dd, *J* = 11.4, 5.0 Hz, 1H), 3.96 (ddd, *J* = 12.4, 11.3, 1.1 Hz, 1H), 3.88–3.71 (m, 2H), 3.62–3.55 (m, 2H), 3.53 (dt, *J* = 12.4, 5.0 Hz, 1H), 2.70–2.59 (br.s, 5H), 1.91–1.81 (m, 3H), 1.76–1.66 (m, 2H), 1.63–1.40 (m, 7H); ^13^C NMR (101 MHz, CDCl_3_) *δ* 168.7, 165.5, 137.0, 132.6, 129.1, 128.7, 127.9, 125.1, 65.2, 49.2, 48.4, 46.6, 43.2, 42.5, 41.2, 30.0, 28.0, 25.6; HRMS (ESI+), *m/z* calcd for C_21_H_30_N_3_O_2_ [M + H]^+^ 356.2333, found 356.2323.

##### 4.2.1.27. 4-([1,4'-Bipiperidine]-1'-carbonyl)-7-fluoro-3,4-dihydroisoquinolin-1(2H)-one (3aa)

This compound was purified with HPLC using MeCN–H_2_O as eluent with addition of CF_3_CO_2_H and isolated as trifluoroacetate salt. Yield 102 mg (43%); Light yellow viscous oil; ^1^H NMR (400 MHz, acetone-*d*_6_) *δ* 7.66 (d, *J* = 9.0 Hz, 1H), 7.44–7.17 (m, 3H), 5.28 (br.s, 4H), 4.78 (br.s, 1H), 4.55 (br.s, 1H), 4.40 (br.s, 1H), 3.75 (br.s, 1H), 3.71–3.56 (m, 4H), 3.37 (br.s, 1H), 3.10 (br.s, 2H), 2.33 (br.s, 2H), 2.00–1.75 (m, 5H); ^13^C NMR (101 MHz, acetone-*d*_6_) *δ* 168.6, 163.9, 162.0 (d, *J* = 244.3 Hz), 134.4, 132.5, 128.7, 118.6 (d, *J* = 21.9 Hz), 113.8 (d, *J* = 23.0 Hz), 63.5, 50.0, 49.9, 44.2, 42.8, 42.7, 40.4, 39.7, 27.0, 26.1, 23.0, 21.7; HRMS (ESI+), *m/z* calcd for C_20_H_27_FN_3_O_2_ [M + H]^+^ 360.2082, found 360.2094.

##### 4.2.1.28. 7-Fluoro-4–(4-methyl-[1,4'-bipiperidine]-1'-carbonyl)-3,4-dihydroisoquinolin-1(2H)-one (3ab)

Yield 120 mg (64%); White solid; m. p. = 171–172 °C; ^1^H NMR (400 MHz, CDCl_3_) *δ* 7.80 (dd, *J* = 8.8, 2.4 Hz, 1H), 7.23–6.98 (m, 2H), 6.80 (d, *J* = 2.4 Hz, 1H), 4.78 (d, *J* = 11.4 Hz, 1H), 4.36–4.25 (m, 1H), 4.02–3.82 (m, 2H), 3.54 (dt, *J* = 12.1, 4.6 Hz, 1H), 3.18 (br.s, 1H), 3.02–2.84 (m, 2H), 2.84–2.40 (m, 2H), 2.23–2.07 (m, 2H), 2.07–1.90 (m, 3H), 1.74–1.61 (m, 2H), 1.61–1.45 (m, 2H), 1.31–1.17 (m, 2H), 0.94 (d, *J* = 6.1 Hz, 3H); ^13^C NMR (101 MHz, CDCl_3_) *δ* 168.4, 164.6, 162.3 (d, *J* = 247.5 Hz), 132.9, 131.4 (d, *J* = 7.5 Hz), 127.3 (d, *J* = 7.5 Hz), 119.4 (d, *J* = 21.3 Hz), 115.4 (d, *J* = 23.4 Hz), 62.1, 46.2, 45.9, 43.2, 41.9, 40.6, 34.6, 31.0, 21.9; HRMS (ESI+), *m/z* calcd for C_21_H_29_FN_3_O_2_ [M + H]^+^ 374.2238, found 374.2239.

##### 4.2.1.29. 7-Fluoro-4–(4-(pyrrolidin-1-yl)piperidine-1-carbonyl)-3,4-dihydroisoquinolin-1(2H)-one (3ac)

Yield 116 mg (67%); Beige solid; m. p. = 141–142 °C; ^1^H NMR (400 MHz, CDCl_3_) *δ* 7.80 (d, *J* = 7.7 Hz, 1H), 7.22–7.01 (m, 2H), 6.62 (br.s, 1H), 4.63 (br.s, 1H), 4.30 (br.s, 1H), 3.90 (t, *J* = 10.9 Hz, 2H), 3.53 (br.s, 1H), 3.25 (br.s, 1H), 2.89 (br.s, 1H), 2.64 (br.s, 4H), 2.32 (br.s, 1H), 2.04 (br.s, 2H), 1.84 (br.s, 4H), 1.69–1.52 (m, 2H); ^13^C NMR (101 MHz, CDCl_3_) *δ* 168.3, 164.6, 162.3 (d, *J* = 248.2 Hz), 132.9, 127.3, 122.5, 119.5, 115.5, 115.3, 61.4, 51.7, 43.2, 40.6, 32.0, 31.3, 23.2; HRMS (ESI+), *m/z* calcd for C_19_H_25_FN_3_O_2_ [M + H]^+^ 346.1925, found 346.1923.

##### 4.2.1.30. 4–(4-(Diethylamino)piperidine-1-carbonyl)-7-fluoro-3,4-dihydroisoquinolin-1(2H)-one (3ad)

Yield 80 mg (46%); Beige solid; m. p. = 134–135 °C; ^1^H NMR (400 MHz, CDCl_3_) *δ* 7.80 (dd, *J* = 8.9, 2.7 Hz, 1H), 7.24–6.98 (m, 2H), 6.85 (br.s, 1H), 4.79 (d, *J* = 12.2 Hz, 1H), 4.32 (dd, *J* = 10.3, 4.2 Hz, 1H), 4.02–3.83 (m, 2H), 3.55 (dt, *J* = 12.4, 4.8 Hz, 1H), 3.25–3.13 (m, 1H), 2.90–2.69 (m, 2H), 2.69–2.55 (m, 4H), 2.01–1.86 (m, 2H), 1.60–1.45 (m, 2H), 1.07 (t, *J* = 7.1 Hz, 6H); ^13^C NMR (101 MHz, CDCl_3_) *δ* 168.2, 164.7, 162.3 (d, *J* = 247.5 Hz), 132.9, 131.4 (d, *J* = 7.6 Hz), 127.3 (d, *J* = 7.5 Hz), 119.4 (d, *J* = 21.2 Hz), 115.3 (d, *J* = 23.0 Hz), 57.9, 46.1, 43.5, 43.3, 43.2, 42.1, 40.6, 29.9, 28.5, 13.6, 13.2; HRMS (ESI+), *m/z* calcd for C_19_H_27_FN_3_O_2_ [M + H]^+^ 348.2082, found 348.2081.

##### 4.2.1.31. 4-(4-(Azepan-1-yl)piperidine-1-carbonyl)-7-fluoro-3,4-dihydroisoquinolin-1(2H)-one (3ae)

Yield 86 mg (46%); White solid; m. p. = 179–180 °C; ^1^H NMR (400 MHz, CDCl_3_) *δ* 7.78 (dd, *J* = 8.8, 2.6 Hz, 1H), 7.23–6.98 (m, 3H), 4.75 (br.s, 1H), 4.35–4.26 (m, 1H), 4.02–3.80 (m, 2H), 3.55 (dt, *J* = 12.4, 4.7 Hz, 1H), 3.23–3.11 (m, 1H), 2.86–2.65 (m, 6H), 2.00–1.88 (m, 2H), 1.70–1.56 (m, 8H), 1.56–1.44 (m, 2H); ^13^C NMR (101 MHz, CDCl_3_) *δ* 168.3, 164.8, 162.2 (d, *J* = 247.4 Hz), 133.0, 131.5 (d, *J* = 7.3 Hz), 127.4, 119.3 (d, *J* = 22.2 Hz), 115.3 (d, *J* = 23.2 Hz), 62.1, 61.2, 51.4, 46.0, 45.5, 43.1, 42.0, 41.8, 40.5, 29.6, 29.1, 28.3, 27.0; HRMS (ESI+), *m/z* calcd for C_21_H_29_FN_3_O_2_ [M + H]^+^ 374.2238, found 374.2249.

##### 4.2.1.32. 4–(3,3-Dimethyl-[1,4'-bipiperidine]-1'-carbonyl)-7-fluoro-3,4-dihydroisoquinolin-1(2H)-one (3af)

Yield 114 mg (59%); Light pink solid; m. p. = 182–183 °C; ^1^H NMR (400 MHz, CDCl_3_) *δ* 7.80 (dd, *J* = 8.8, 2.3 Hz, 1H), 7.26–6.99 (m, 2H), 6.81–6.67 (m, 1H), 4.68 (br.s, 1H), 4.36–4.27 (m, 1H), 3.99–3.85 (m, 2H), 3.55 (dt, *J* = 12.4, 4.8 Hz, 1H), 3.25–3.13 (m, 1H), 2.89–2.76 (m, 1H), 2.62–2.38 (m, 3H), 2.16 (br.s, 2H), 1.96–1.83 (m, 2H), 1.65–1.48 (m, 4H), 1.30–1.20 (m, 2H), 0.95 (s, 6H); ^13^C NMR (101 MHz, CDCl_3_) *δ* 168.2, 164.6, 162.3 (d, *J* = 247.5 Hz), 132.9, 131.4, 127.3, 119.4 (d, *J* = 21.9 Hz), 115.5, 61.7, 50.7, 45.8, 45.3, 43.2, 41.8, 40.6, 37.9, 30.8, 29.1, 28.1, 27.3, 23.0;HRMS (ESI+), *m/z* calcd for C_22_H_31_FN_3_O_2_ [M + H]^+^ 388.2395, found 388.2406.

##### 4.2.1.33. 4–(4,4-Dimethyl-[1,4'-bipiperidine]-1'-carbonyl)-7-fluoro-3,4-dihydroisoquinolin-1(2H)-one (3ag)

Yield 78 mg (40%); White solid; m. p. = 200–201 °C; ^1^H NMR (400 MHz, CDCl_3_) *δ* 7.80 (dd, *J* = 8.8, 2.7 Hz, 1H), 7.23–6.98 (m, 2H), 6.86 (d, *J* = 2.9 Hz, 1H), 4.78 (d, *J* = 10.8 Hz, 1H), 4.31 (dd, *J* = 10.7, 4.4 Hz, 1H), 4.02–3.83 (m, 2H), 3.54 (dt, *J* = 12.4, 4.8 Hz, 1H), 3.25–3.13 (m, 1H), 2.84–2.67 (m, 1H), 2.63–2.43 (m, 5H), 2.12–1.93 (m, 2H), 1.55 (qd, *J* = 12.4, 4.0 Hz, 2H), 1.44 (br.s, 4H), 0.94 (s, 6H); ^13^C NMR (101 MHz, CDCl_3_) *δ* 168.3, 164.7, 162.3 (d, *J* = 247.5 Hz), 132.9, 131.4 (d, *J* = 7.4 Hz), 127.2 (d, *J* = 7.7 Hz), 119.4 (d, *J* = 25.3 Hz), 115.4 (d, *J* = 23.2 Hz), 62.0, 61.8, 46.1, 45.8, 43.2, 41.9, 41.5, 40.6, 38.9, 28.6, 28.2; HRMS (ESI+), *m/z* calcd for C_22_H_31_FN_3_O_2_ [M + H]^+^ 388.2395, found 388.2390.

##### 4.2.1.34. 4–(4,4-Difluoro-[1,4'-bipiperidine]-1'-carbonyl)-7-fluoro-3,4-dihydroisoquinolin-1(2H)-one (3ah)

Yield 99 mg (50%); Light brown viscous oil; ^1^H NMR (400 MHz, CDCl_3_) *δ* 7.80 (dd, *J* = 8.8, 2.4 Hz, 1H), 7.23–6.99 (m, 2H), 6.89 (br.s, 1H), 4.77 (br.s, 1H), 4.35–4.26 (m, 1H), 4.05–3.81 (m, 2H), 3.56 (dt, *J* = 12.3, 4.7 Hz, 1H), 2.84–2.58 (m, 5H), 2.12–1.84 (m, 7H), 1.62–1.45 (m, 2H); ^13^C NMR (101 MHz, CDCl_3_) *δ* 168.3, 164.7, 162.3 (d, *J* = 247.5 Hz), 132.8, 131.4 (d, *J* = 8.1 Hz), 127.2 (d, *J* = 7.5 Hz), 119.4 (d, *J* = 22.1 Hz), 115.4 (d, *J* = 23.4 Hz), 61.3, 60.9, 45.9, 45.8, 43.2, 41.8, 40.6, 34.6, 34.4, 34.1, 29.3, 28.3; HRMS (ESI+), *m/z* calcd for C_20_H_25_F_3_N_3_O_2_ [M + H]^+^ 396.1893, found 396.1883.

##### 4.2.1.35. 7-Fluoro-4–(4-fluoro-[1,4'-bipiperidine]-1'-carbonyl)-3,4-dihydroisoquinolin-1(2H)-one (3ai)

Yield 138 mg (73%); Light brown viscous oil; ^1^H NMR (400 MHz, CDCl_3_) *δ* 7.76 (dd, *J* = 8.9, 2.7 Hz, 1H), 7.26 (br.s, 1H), 7.22–6.97 (m, 2H), 4.82–4.59 (m, 2H), 4.30 (dd, *J* = 10.4, 4.8 Hz, 1H), 4.03–3.78 (m, 2H), 3.54 (dt, *J* = 12.5, 4.7 Hz, 1H), 3.25–3.11 (m, 1H), 2.83–2.66 (m, 3H), 2.64–2.47 (m, 3H), 2.03–1.82 (m, 6H), 1.53 (qd, *J* = 12.3, 3.8 Hz, 2H); ^13^C NMR (101 MHz, CDCl_3_) *δ* 168.3, 164.8, 162.2 (d, *J* = 247.5 Hz), 133.0, 131.5 (d, *J* = 7.4 Hz), 127.3 (d, *J* = 7.5 Hz), 119.3 (d, *J* = 21.9 Hz), 115.2 (d, *J* = 23.5 Hz), 88.4 (d, *J* = 170.2 Hz), 77.3, 61.8, 61.5, 45.3, 43.1, 41.8, 41.5, 40.5, 31.7 (d, *J* = 19.3 Hz), 29.2, 28.1; HRMS (ESI+), *m/z* calcd for C_20_H_26_F_2_N_3_O_2_ [M + H]^+^ 378.1988, found 378.1999.

##### 4.2.1.36. 7-Fluoro-4–(4-methoxy-[1,4'-bipiperidine]-1'-carbonyl)-3,4-dihydroisoquinolin-1(2H)-one (3aj)

Yield 127 mg (65%); Beige solid; m. p. = 186–187 °C; ^1^H NMR (400 MHz, CDCl_3_) *δ* 7.79 (dd, *J* = 8.9, 2.8 Hz, 1H), 7.23–6.98 (m, 2H), 6.89 (br.s, 1H), 4.76 (d, *J* = 12.2 Hz, 1H), 4.30 (dd, *J* = 10.6, 4.7 Hz, 1H), 4.02–3.81 (m, 2H), 3.54 (dt, *J* = 12.5, 4.8 Hz, 1H), 3.35 (s, 3H), 3.28–3.12 (m, 2H), 2.89–2.77 (m, 2H), 2.62–2.47 (m, 1H), 2.39–2.30 (m, 2H), 2.05–1.89 (m, 5H), 1.69–1.46 (m, 4H); ^13^C NMR (101 MHz, CDCl_3_) *δ* 168.4, 164.6, 162.3 (d, *J* = 247.6 Hz), 132.9, 131.4 (d, *J* = 7.6 Hz), 127.2 (d, *J* = 7.5 Hz), 119.4 (d, *J* = 22.4 Hz), 115.3 (d, *J* = 23.0 Hz), 76.2, 61.7, 61.4, 55.5, 46.8, 45.8, 45.3, 43.2, 41.8, 40.6, 31.1, 29.4, 28.1; HRMS (ESI+), *m/z* calcd for C_21_H_29_FN_3_O_3_ [M + H]^+^ 390.2187, found 390.2189.

##### 4.2.1.37. *4-([1,4'-Bipiperidine]-1'-carbonyl)isoquinolin-1(2H)-one (*11*).*

Yield 110 mg (65%); Pale yellow solid; m. p. = 171–174 °C; ^1^H NMR (400 MHz, DMSO-*d*_6_) *δ* 11.52 (br.s, 1H), 8.25 (d, *J* = 7.6 Hz, 1H), 7.75 (t, *J* = 7.5 Hz, 1H), 7.54 (t, *J* = 7.6 Hz, 1H), 7.50 (br.s, 1H), 7.26 (br.s, 1H), 4.56 (br.s, 1H), 3.63 (br.s, 1H), 3.02–2.74 (m, 2H), 2.48–2.37 (m, 5H), 1.85–1.58 (m, 2H), 1.51–1.41 (m, 5H), 1.40–1.30 (m, 3H); ^13^C NMR (101 MHz, DMSO-*d*_6_) *δ* 165.7, 161.7, 135.4, 133.3, 128.4, 127.6, 127.3, 126.2, 124.4, 113.1, 62.0, 50.2, 47.0, 28.2, 26.5, 25.0; HRMS (ESI+), *m/z* calcd for C_20_H_26_N_3_O_2_ [M + H]^+^ 340.2020, found 340.2020.

### 4.3. *In vitro* biological testing

#### 4.3.1. Parp1 and PARP2 inhibition assays

The compounds’ inhibitory activity towards PARP1 and PARP1 was assessed using the commercially available colorimetric activity assay kit from BPS Bioscience (San Diego, CA) in full accordance of the supplier’s method description^15^^,^^16^

#### 4.3.2. Aqueous PBS solubility determination

The stock solution of the compound tested in DMSO (10 mM) was diluted with 0.01 M sodium phosphate buffer solution (pH 7.4) to the concentration of 100 µM, 80 µM, 60 µM, 50 µM, 40 µM, 30 µM, 20 µM and 10 µM. The serially diluted solutions were transferred into a 96-well plate (Corning 3635, UV Plate) and the plate was incubated at 37 °C for 2 h using BioSan PST-60HL-4 thermo shaker. The plate was read at the wavelength of 620 nM using spectrophotometric microplate reader Sunrise (Tecan, Australia). The optical density values were plotted against compound concentration and approximated by a linear relationship to determine the aqueous solubility.

#### 4.3.3. Microsomal and S9 stability determination

The rate of enzymatic degradation of the compound in *liver microsomes* was determined by incubating a mixture containing 0.5 mg/mL of pooled human liver microsomes (XenoTech, USA, cat. # H2620), 10 μM of compound tested, 2 mM β-nicotinamide adenine dinucleotide (Carbosynth, UK, cat. # NN10871) and 4 mM magnesium chloride in 0.1 M sodium phosphate buffer (pH 7.4) in a solid-state thermostat at 37 °C. The rate of enzymatic degradation of the compound in *human liver S9 fraction* was determined by incubating a reaction mixture containing 0.5 mg/mL of pooled S9 human liver fractions (XenoTech, USA, cat # H0610), 10 μM of compound tested, 2 mM β-nicotinamide adenine dinucleotide (Carbosynth, UK, cat # NN10871) and 4 mM magnesium chloride in 0.1 M sodium phosphate buffer (pH 7.4) in a solid-state thermostat at 37 °C. The reaction was stopped with acetonitrile (100 μL MeCN per 100 μL of the reaction mixture). After stopping the reaction, the samples were centrifuged for 10 min at 10,000 rpm. The supernatant was analysed by chromatography using an Agilent 1260 chromatograph (Agilent, United States). Chromatographic analysis was performed in a gradient elution mode at a flow rate of 1 ml/min. A graph of the dependence of the logarithm of the area of ​​the peak of the substance on time was built. The dependent coefficient of this straight line corresponded to the elimination constant *k*, on the basis of which the half-life of the drug (T_1/2_) and intrinsic clearance (Cl_int_) were calculated.

#### 4.3.6. Plasma stability determination

Determination of stability in human blood plasma was carried out using pooled human blood plasma obtained from ten healthy donors. The candidate stock solution (10 mM in DMSO) was diluted with the pooled blood plasma to a concentration of 10 μM (test solution). The test solution was kept in a solid-state thermostat for 4 h at a temperature of 37 °C. HPLC method using an Agilent 1260 chromatograph (Agilent, USA) was employed to determine the peak area of the compounds in the test samples corresponding to the initial test time (before exposure) and the final test time (after incubation in a solid-state thermostat for 4 h at 37 °C) with preliminary precipitation of protein mass with acetonitrile (300 μL MeCN per 100 μL of the reaction mixture). After stopping the reaction, the samples were centrifuged for 10 min at 10,000 rpm and the supernatant was collected. Chromatographic analysis was performed in a gradient elution mode at a flow rate of 1 ml/min.

#### 4.3.7. Plasma protein binding determination

Determination of binding to human blood plasma proteins was carried out using pooled human blood plasma obtained from ten healthy donors. Determination was performed using Thermo Scientific ™ Single-RED dialysis plates. Test solutions of the candidate (10 mM in DMSO) were prepared in blood plasma and in 0.01 M sodium phosphate buffer by dilution in pooled blood plasma to a concentration of 10 μM (test solution 1) and in buffer to a concentration of 10 μM (test solution 2), respectively. The test solutions were transferred to the chambers of the dialysis plate, incubated in an Environmental Shaker – Incubator ES-20/60 (Biosan, Latvia) for 4 h at a temperature of 37 °C at a speed of 130–250 rpm. During the incubation, passive diffusion of the unbound compound occurs, and after 4 h, equilibrium is reached between the chambers with plasma with buffer solution. At the end of the incubation time, samples were taken from the chambers of the plate. HPLC analysis performed using an Agilent 1260 chromatograph (Agilent, USA) allowed determination of the peak area of the compounds in the test samples with preliminary precipitation of proteins with acetonitrile (300 µL of acetonitrile per 100 µL of the reaction mixture). After stopping the reaction, the samples were centrifuged for 10 min at 10,000 rpm and the supernatant was collected.

The concentration of the test compound in the buffer and plasma chambers was calculated using the formula:

$${С}_{}={\frac{A_{t=4}}{A_{t=0}}}_{}\times 10,$$

where $A_{t=0}$ is the value of the peak area of the candidate's test solution on the chromatogram at the initial moment of time;

$A_{t=4}$ is the value of the peak area of the candidate test solution on the chromatogram after the incubation time;

10 is the initial concentration of the candidate in DMSO (10 mM);

The % unbound compound was calculated using the formula:

$$\%\mathrm{Fre}e_{}={\frac{C_{1}}{C_{2}}}_{}\times 100,$$

where $C_{1}$ is the concentration value in the buffer chamber;

$C_{2}$ is the value of the concentration in the plasma chamber.

### 4.4. *In silico* studies

#### 4.4.1. Protein structure preparation

PARP1 and 2 protein structures was downloaded from RCSB Protein Data Bank (models ID: 4ZZX, 4ZZY, 4ZZZ, 5A00). All model needed to be preprocessed with Protein Prep Wizard tool (Schrodinger suite 2020–4) in order to fix incorrect bond orders, missing sidechains, crystal water removed from model, restrained minimisation of protein structure with H-bond network refinement^19^.

#### 4.4.2. Small molecule preparation

Three-dimensional structure of observed small-molecule compounds was generated with use of OPLS3e forcefield. For better accuracy, forcefield was used in order to achieve missing torsions parameterisation^20^.

#### 4.4.3. Protein-ligand docking: GridBox

GridBoxes for used protein models of PARP1 and 2 was generated with the use of the reference ligand coordinates present in all observed models. No constraints were used. GridBox size was 16 Å, in accordance with the reference ligands size. For docking grid generation, GlideGrid module (Schrodinger suite 2020–4) was employed.

#### 4.4.4. Protein-ligand docking: docking and results filtering

Docking of all observed compounds in the active pocket of PARP1 and 2 was performed with use of Glide module (Schrodinger suite 2020–4) ^21^. Reference ligands was redocked into the active site. Best-fitting poses had RMSD less than 1.5Å to initial ligand structure (from PDB model). For each ligand, up to 20 docking solutions were generated. The best-fitting docking pose was selected in accordance with the reference ligand binding mode. Docking solution quality controlled by Emodel parameters and clustering (RMSD ≤2.2 Å in cluster).

#### 4.4.5. Protein-ligand MM-GBSA

Protein-ligand complexes binding free energy and its components was calculated with the use of MM-GBSA method^22^. Calculations were performed in the presence of implicit solvent (water) with residue flexibility cut-off of 6.0 Å.

## Supplementary Material

Supplemental Material
