# Supplementary material for: 1-Oxo-3,4-dihydroisoquinoline-4-carboxamides as novel druglike inhibitors of poly(ADP-ribose) polymerase (PARP) with favourable ADME characteristics
Source: J Enzyme Inhib Med Chem. 2021 Sep 6;36(1):1968–83. doi: 10.1080/14756366.2021.1972993 (PMC8425678; doi:10.1080/14756366.2021.1972993)

Supporting Information

for

3,4-Dihydroisoquinol-1-one-4-carboxamides as novel druglike inhibitors of poly(ADP-ribose) polymerase (PARP) with favorable ADME characteristics

Alexander Safrygin[a], Petr Zhmurov[a], Dmitry Dar'in[a], Sergey Silonov[b], Mariia Kasatkina[b], Yulia Zonis[b], Maxim Gureev[c] and Mikhail Krasavin[a,]*

[a] Saint Petersburg State University, Saint Petersburg, 199034 Russian Federation

[b] JSC BIOCAD, 34a Svyazi Str., Saint Petersburg 198515 Russian Federation

[c] I.M. Sechenov First Moscow State Medical University, Moscow 119991, Russian Federation

*E-mail: m.krasavin@spbu.ru

http://www.krasavin-group.org/

Table of contents

1. ^{1}H and ^{13}C NMR Spectra of **3a** S3
2. ^{1}H and ^{13}C NMR Spectra of **3b** S4
3. ^{1}H and ^{13}C NMR Spectra of **3c** S5
4. ^{1}H and ^{13}C NMR Spectra of **3d** S6
5. ^{1}H and ^{13}C NMR Spectra of **3e** S7
6. ^{1}H and ^{13}C NMR Spectra of **3f** S8
7. ^{1}H and ^{13}C NMR Spectra of **3g** S9
8. ^{1}H and ^{13}C NMR Spectra of **3h** S10
9. ^{1}H and ^{13}C NMR Spectra of **3i** S11
10. ^{1}H and ^{13}C NMR Spectra of **3j** S12
11. ^{1}H and ^{13}C NMR Spectra of **3k** S13
12. ^{1}H and ^{13}C NMR Spectra of **3l** S14
13. ^{1}H and ^{13}C NMR Spectra of **3m** S15
14. ^{1}H and ^{13}C NMR Spectra of **3n** S16
15. ^{1}H and ^{13}C NMR Spectra of **3o** S17
16. ^{1}H and ^{13}C NMR Spectra of **3p** S18
17. ^{1}H and ^{13}C NMR Spectra of **3q** S19
18. ^{1}H and ^{13}C NMR Spectra of **3r** S20
19. ^{1}H and ^{13}C NMR Spectra of **3s** S21
20. ^{1}H and ^{13}C NMR Spectra of **3t** S22
21. ^{1}H and ^{13}C NMR Spectra of **3u** S23
22. ^{1}H and ^{13}C NMR Spectra of **3v** S24
23. ^{1}H and ^{13}C NMR Spectra of **3w** S25
24. ^{1}H and ^{13}C NMR Spectra of **3x** S26
25. ^{1}H and ^{13}C NMR Spectra of **3y** S27
26. ^{1}H and ^{13}C NMR Spectra of **3z** S28
27. ^{1}H and ^{13}C NMR Spectra of **3aa** S29
28. ^{1}H and ^{13}C NMR Spectra of **3ab** S30
29. ^{1}H and ^{13}C NMR Spectra of **3ac** S31

30. ^{1}H and ^{13}C NMR Spectra of **3ad** S32
31. ^{1}H and ^{13}C NMR Spectra of **3ae** S33
32. ^{1}H and ^{13}C NMR Spectra of **3af** S34
33. ^{1}H and ^{13}C NMR Spectra of **3ag** S35
34. ^{1}H and ^{13}C NMR Spectra of **3ah** S36
35. ^{1}H and ^{13}C NMR Spectra of **3ai** S37
36. ^{1}H and ^{13}C NMR Spectra of **3aj** S38
37. ^{1}H and ^{13}C NMR Spectra of **11** S39

1. ^{1}H and ^{13}C NMR Spectra of **3a**

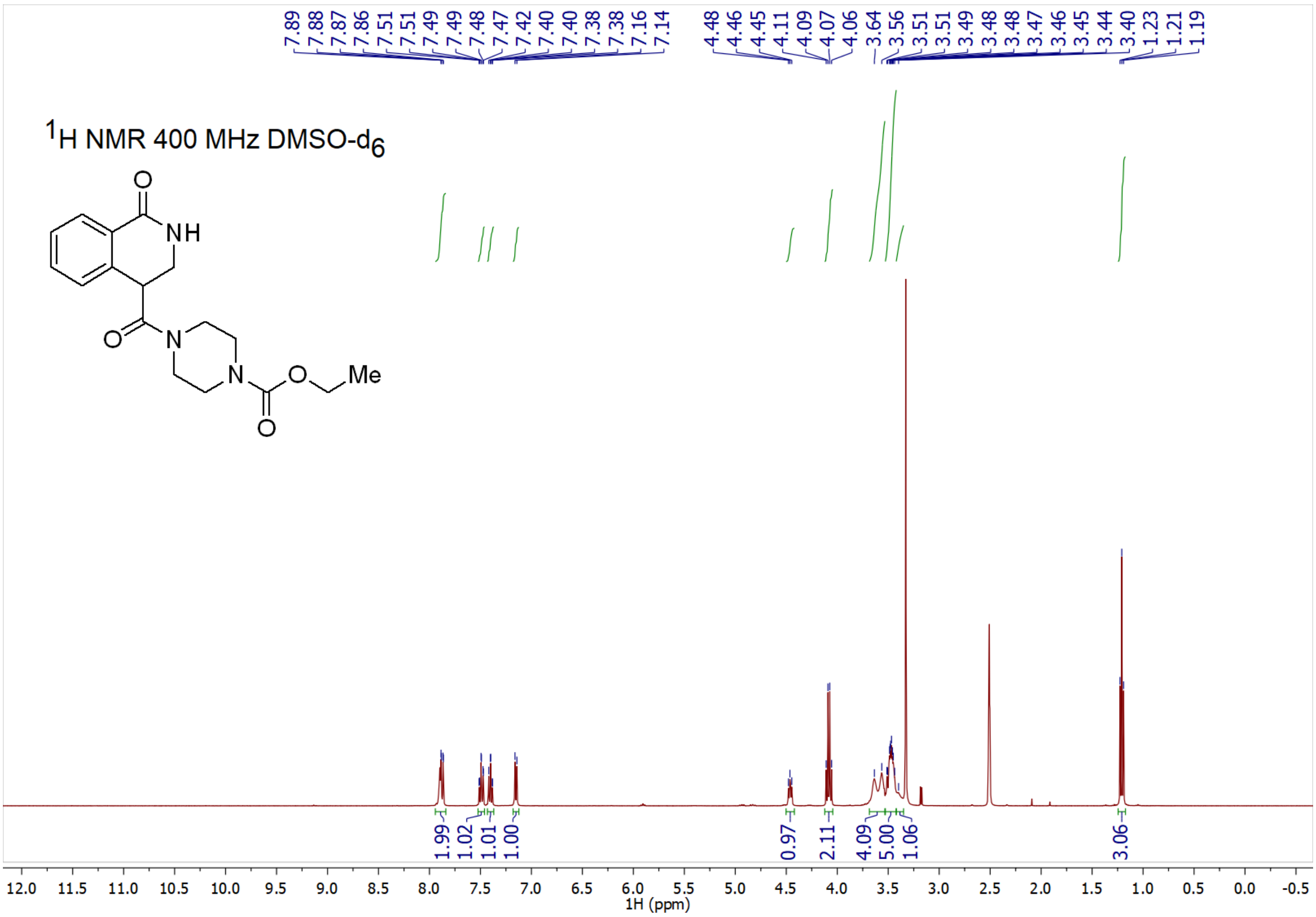

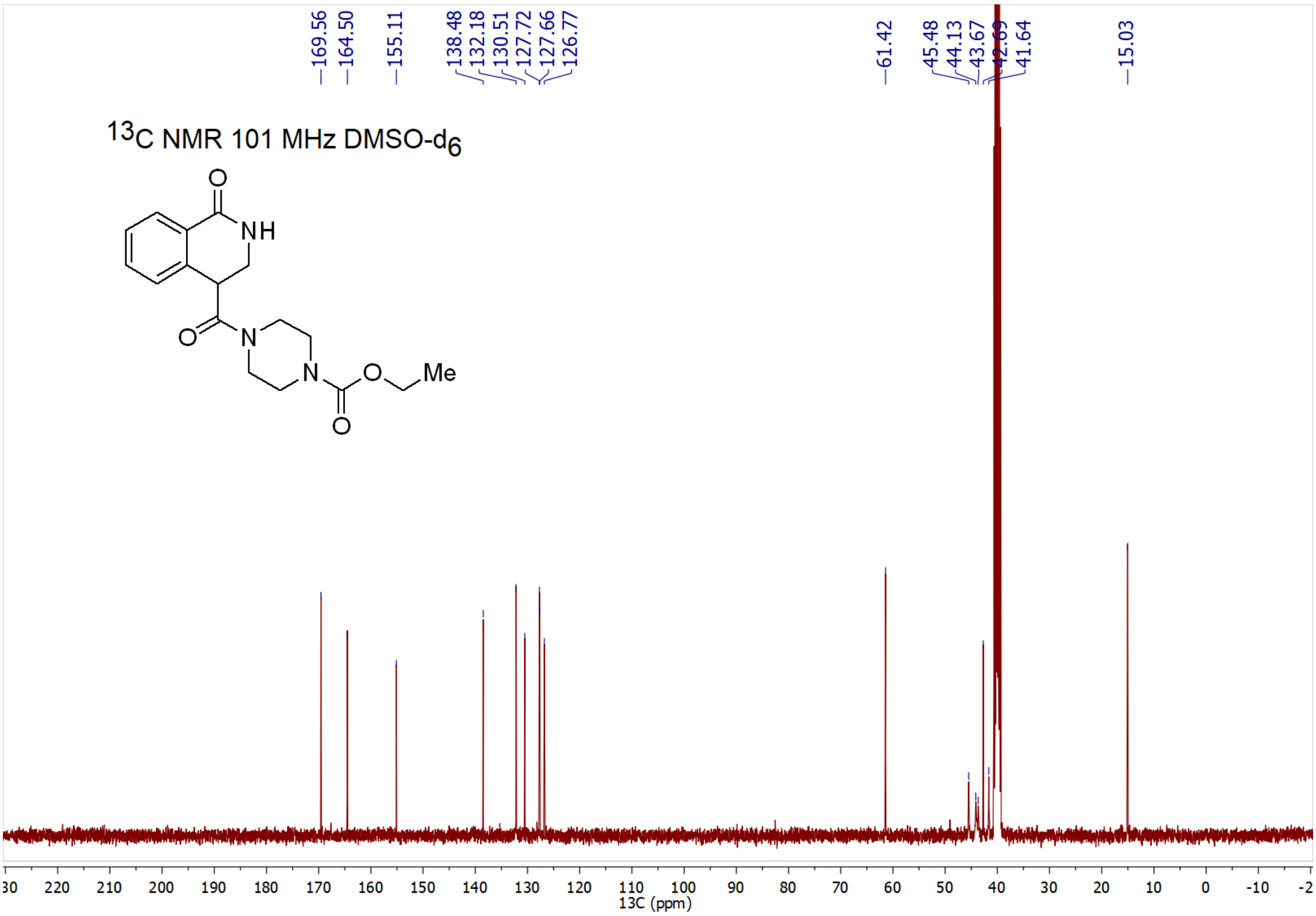

2. ^{1}H and ^{13}C NMR Spectra of **3b**

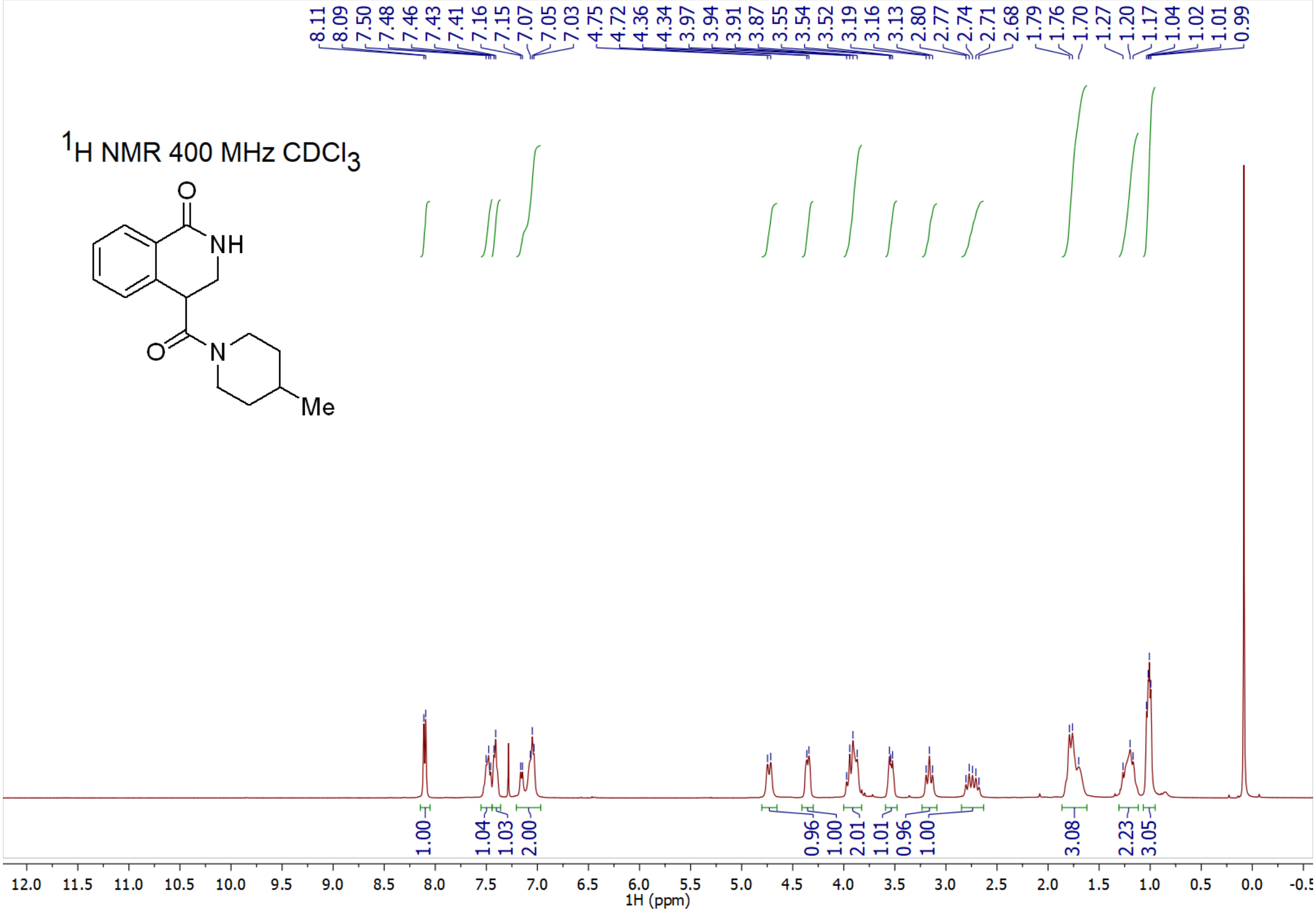

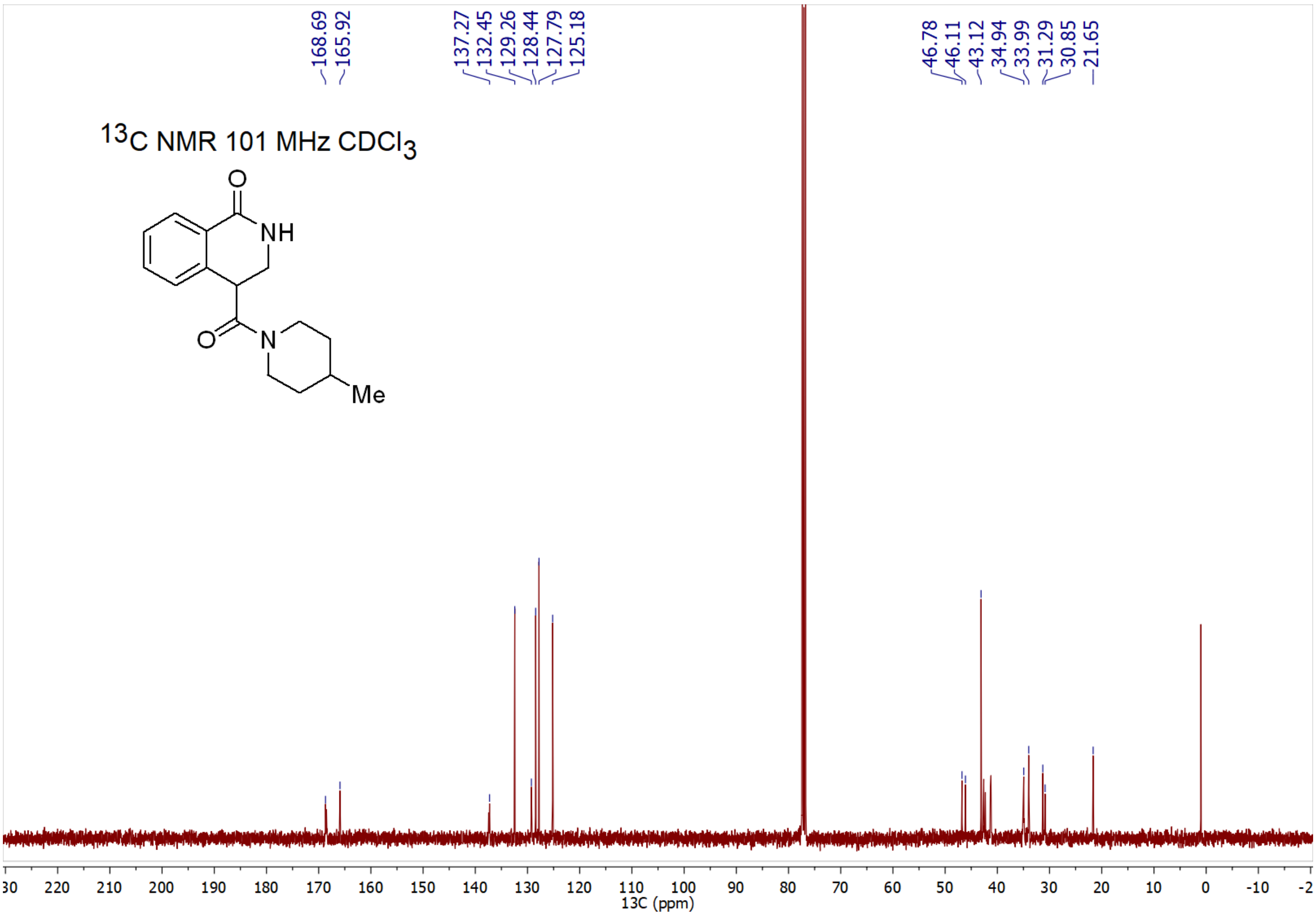

3. ^{1}H and ^{13}C NMR Spectra of **3c**

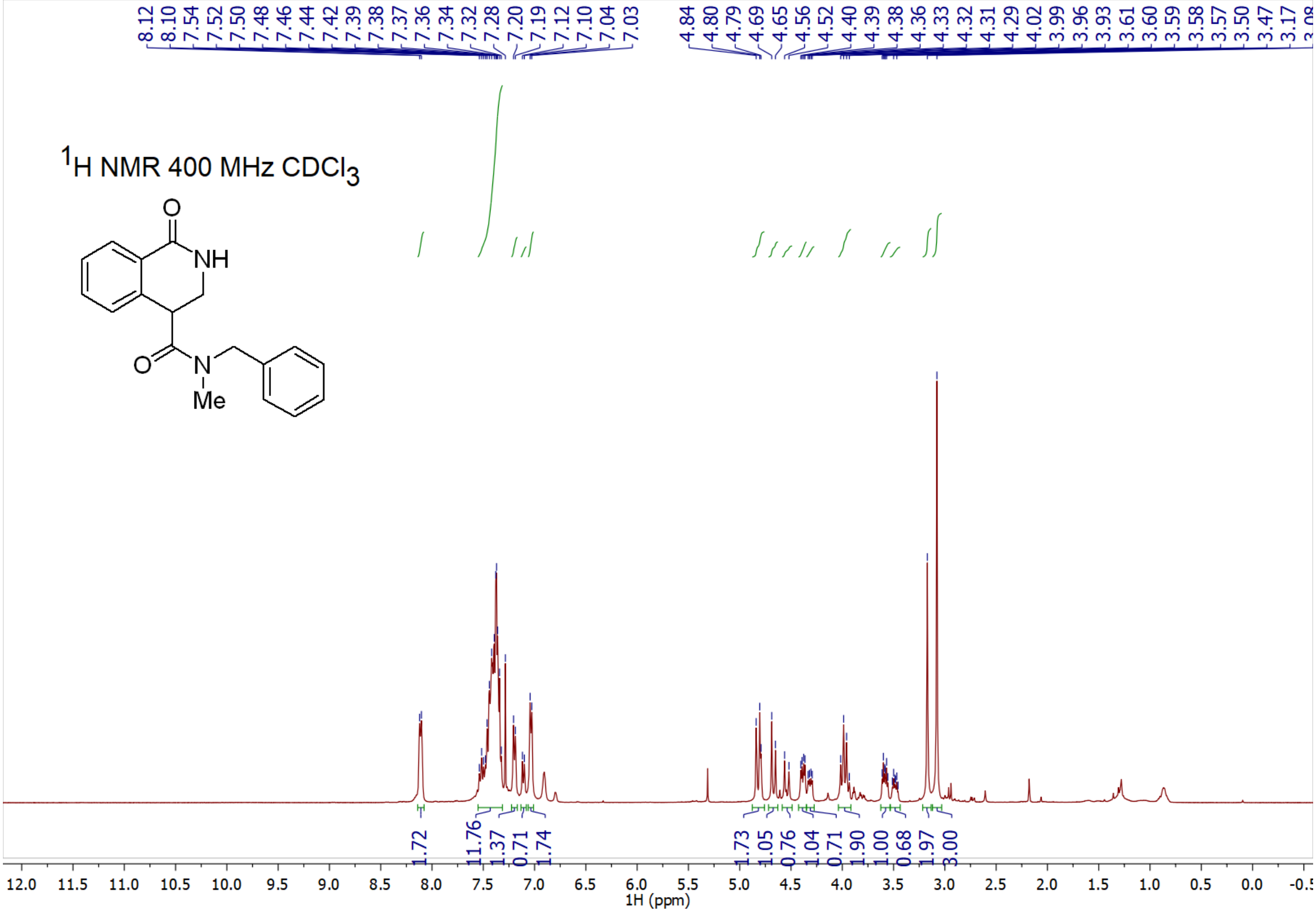

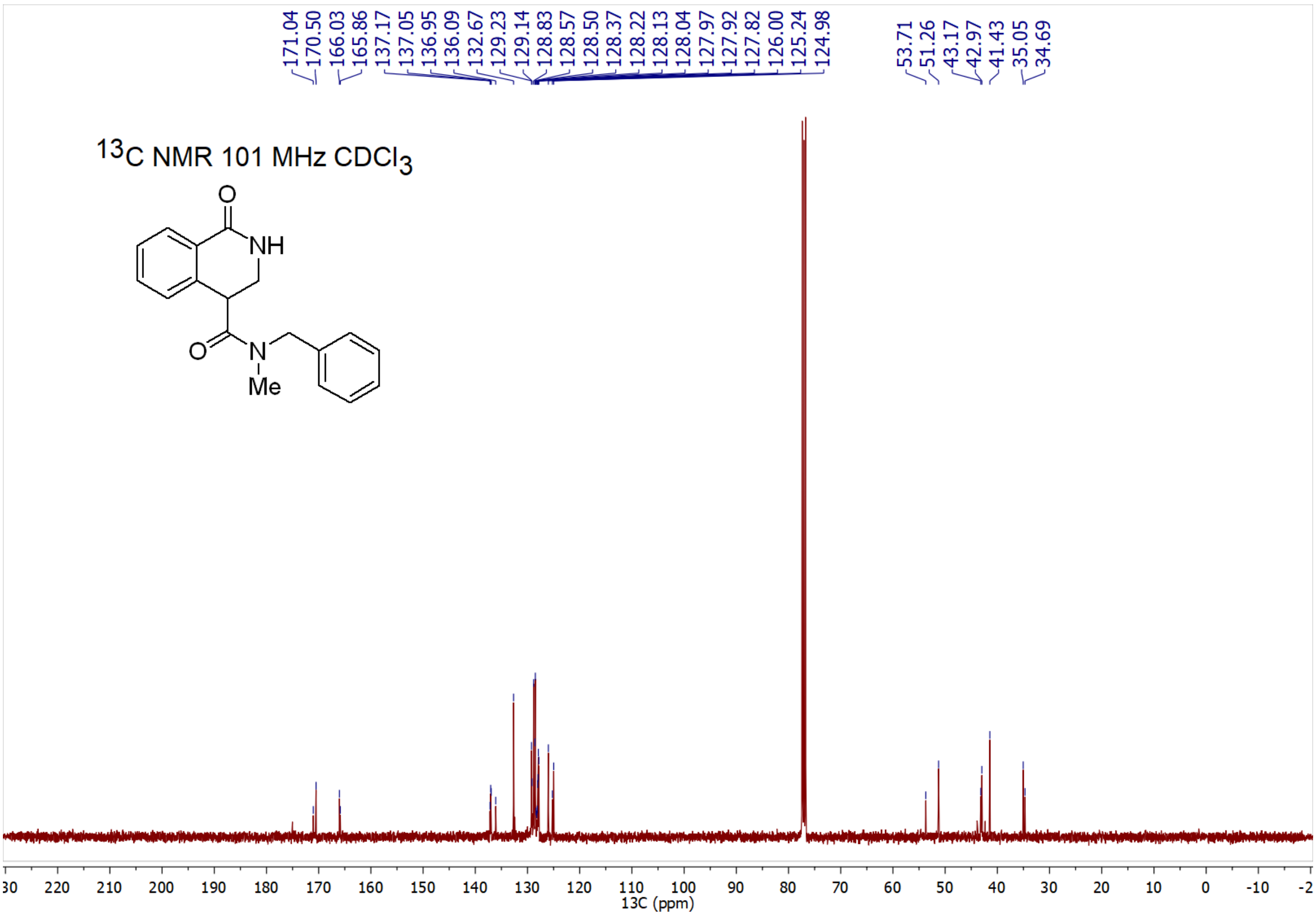

4. ^{1}H and ^{13}C NMR Spectra of **3d**

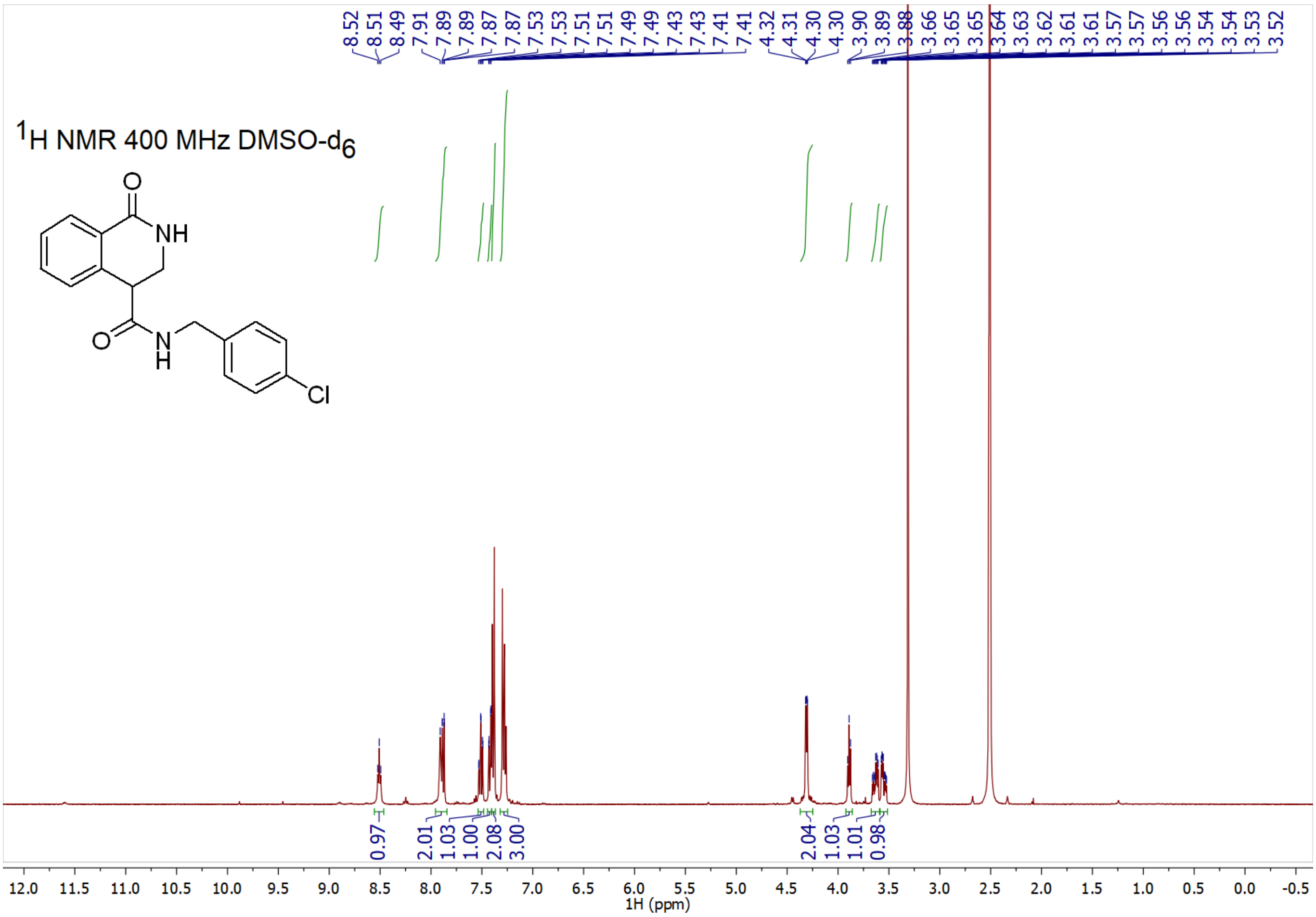

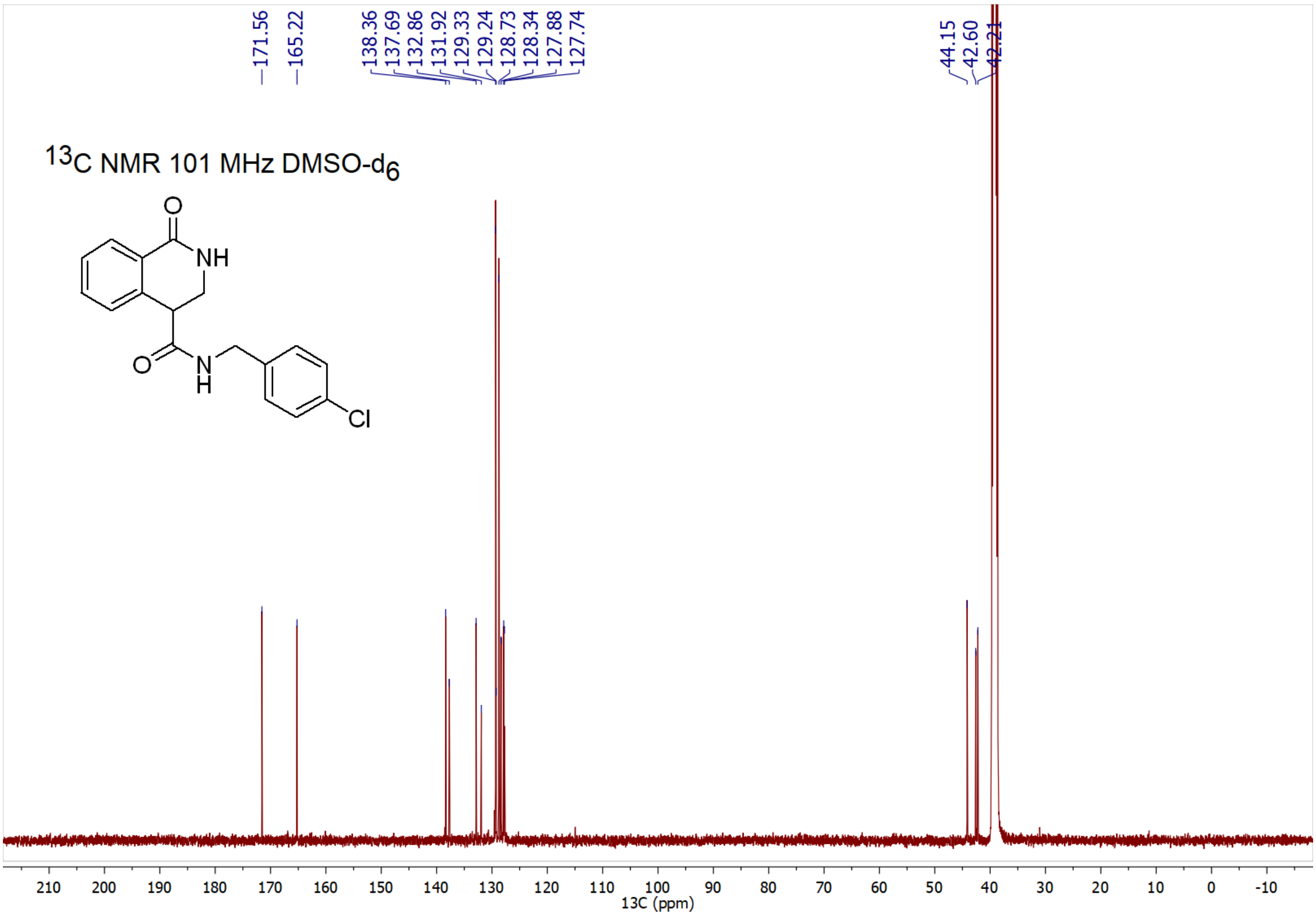

5. ^{1}H and ^{13}C NMR Spectra of **3e**

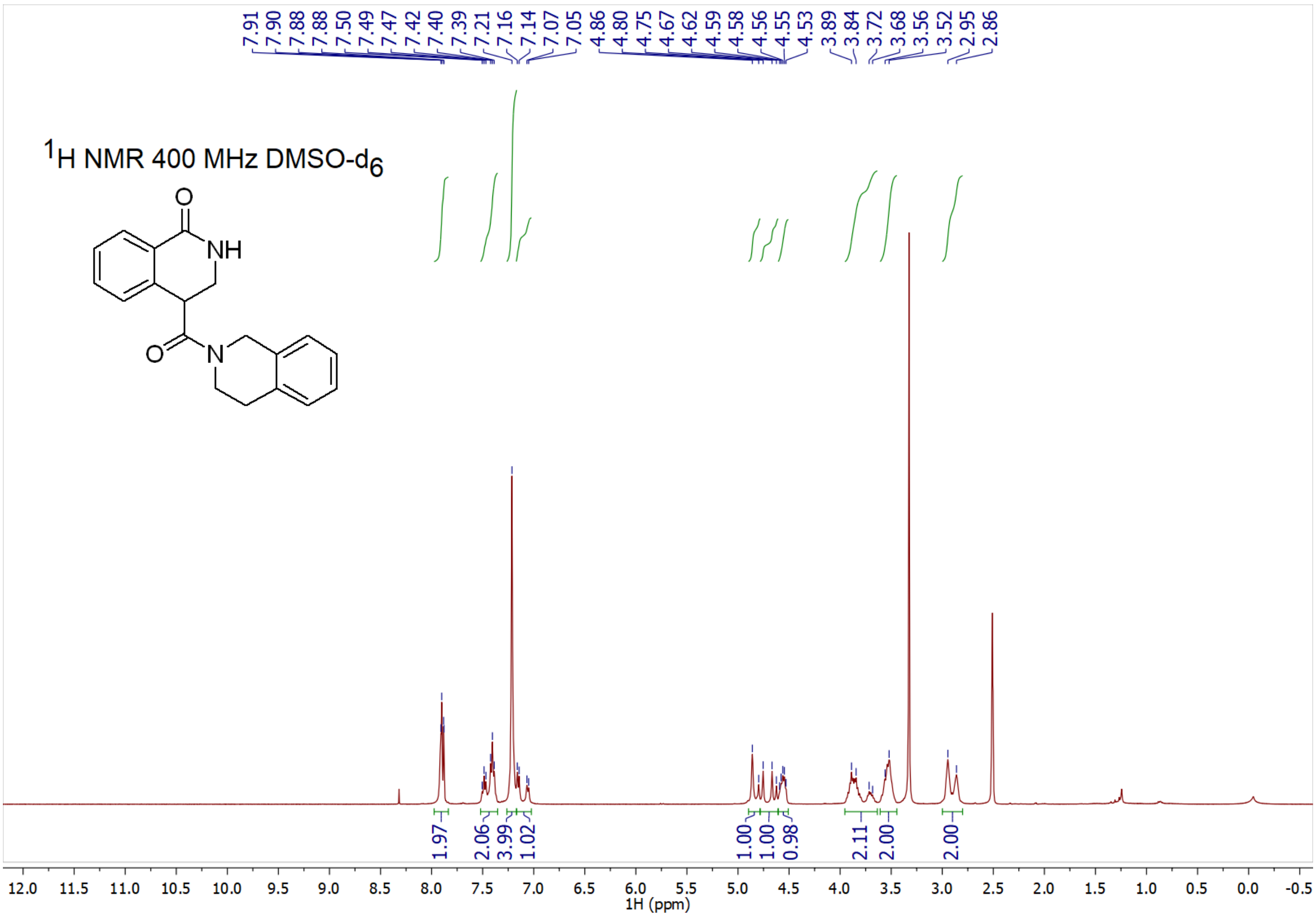

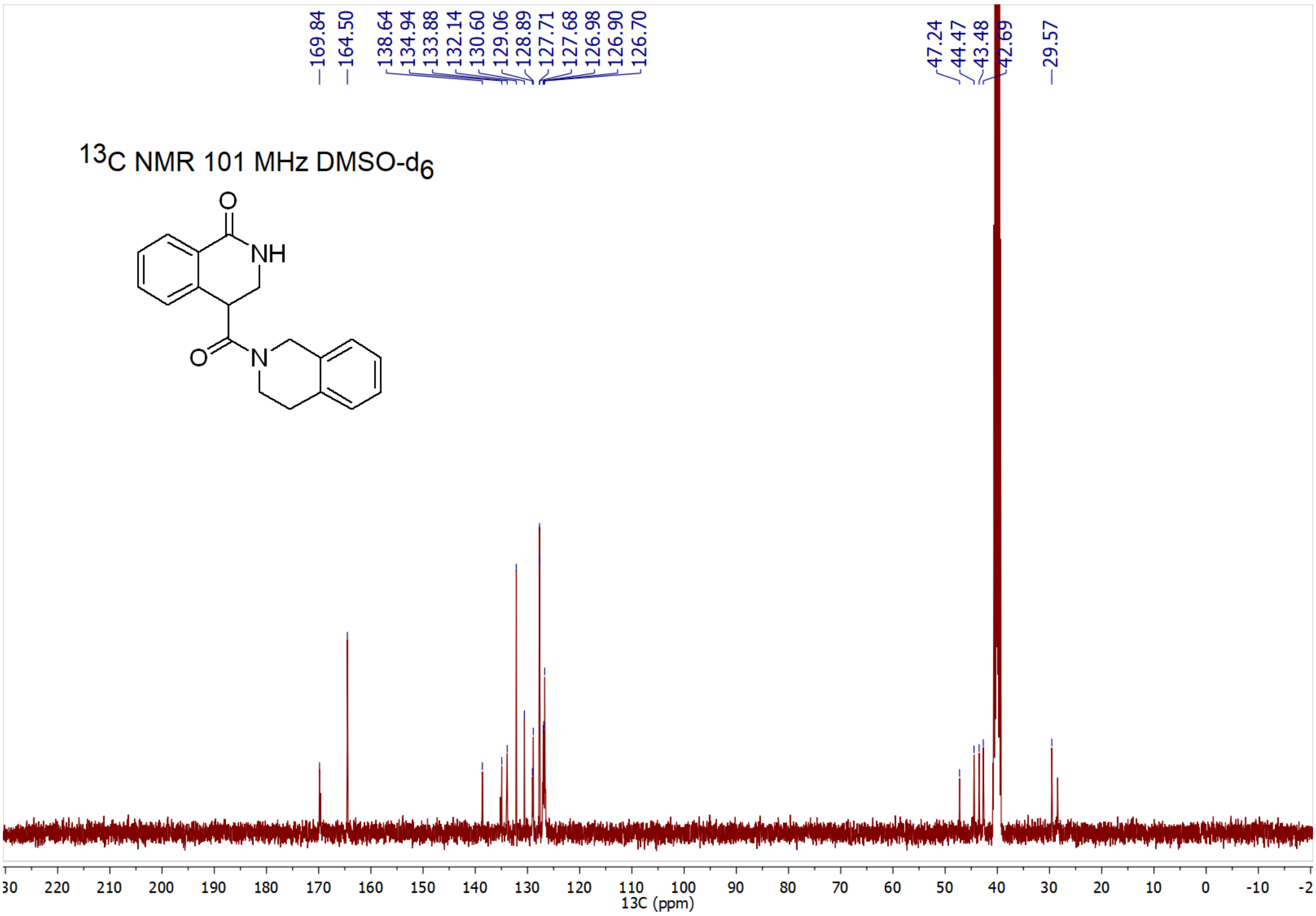

6. ^{1}H and ^{13}C NMR Spectra of **3f**

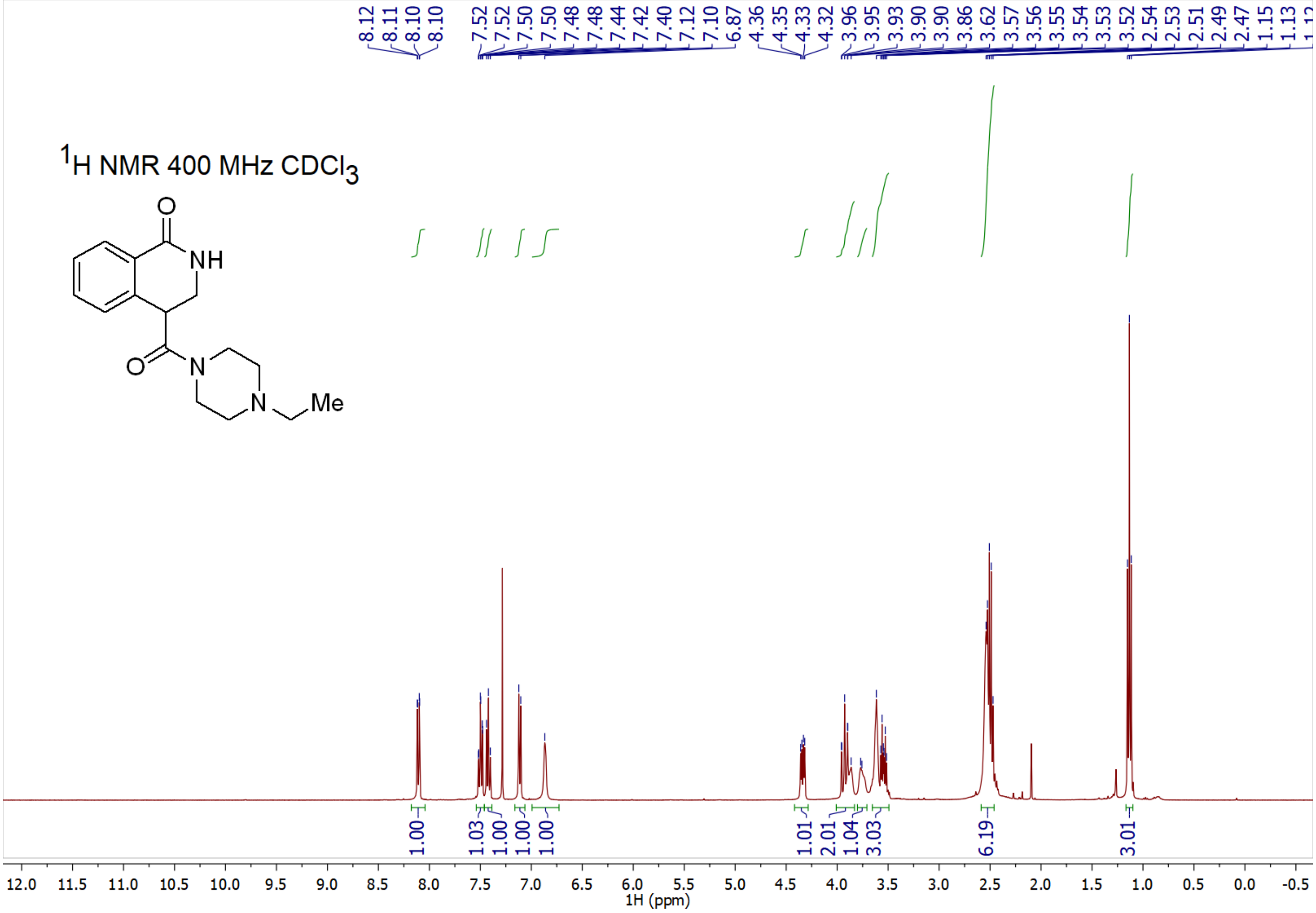

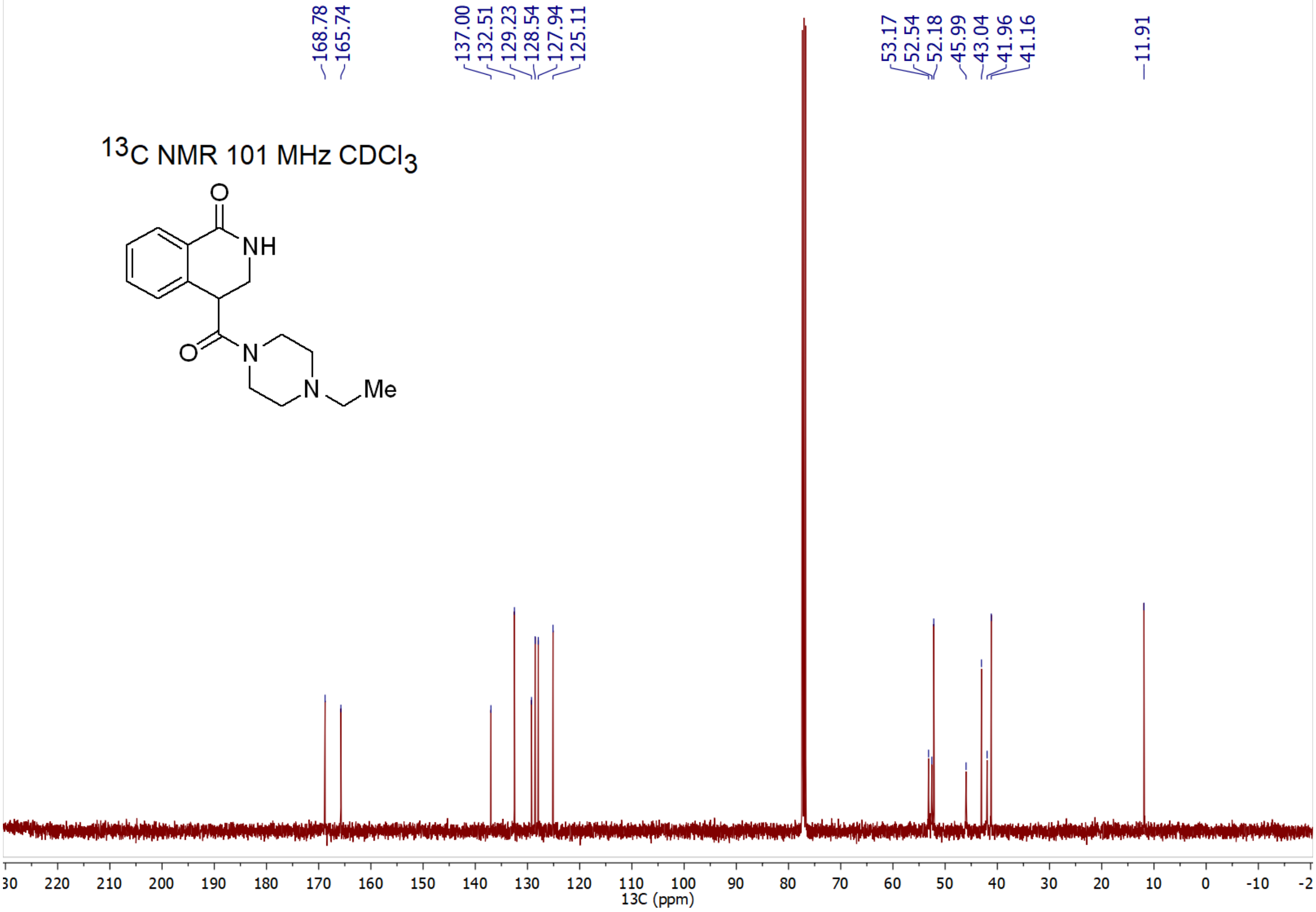

7. ^{1}H and ^{13}C NMR Spectra of **3g**

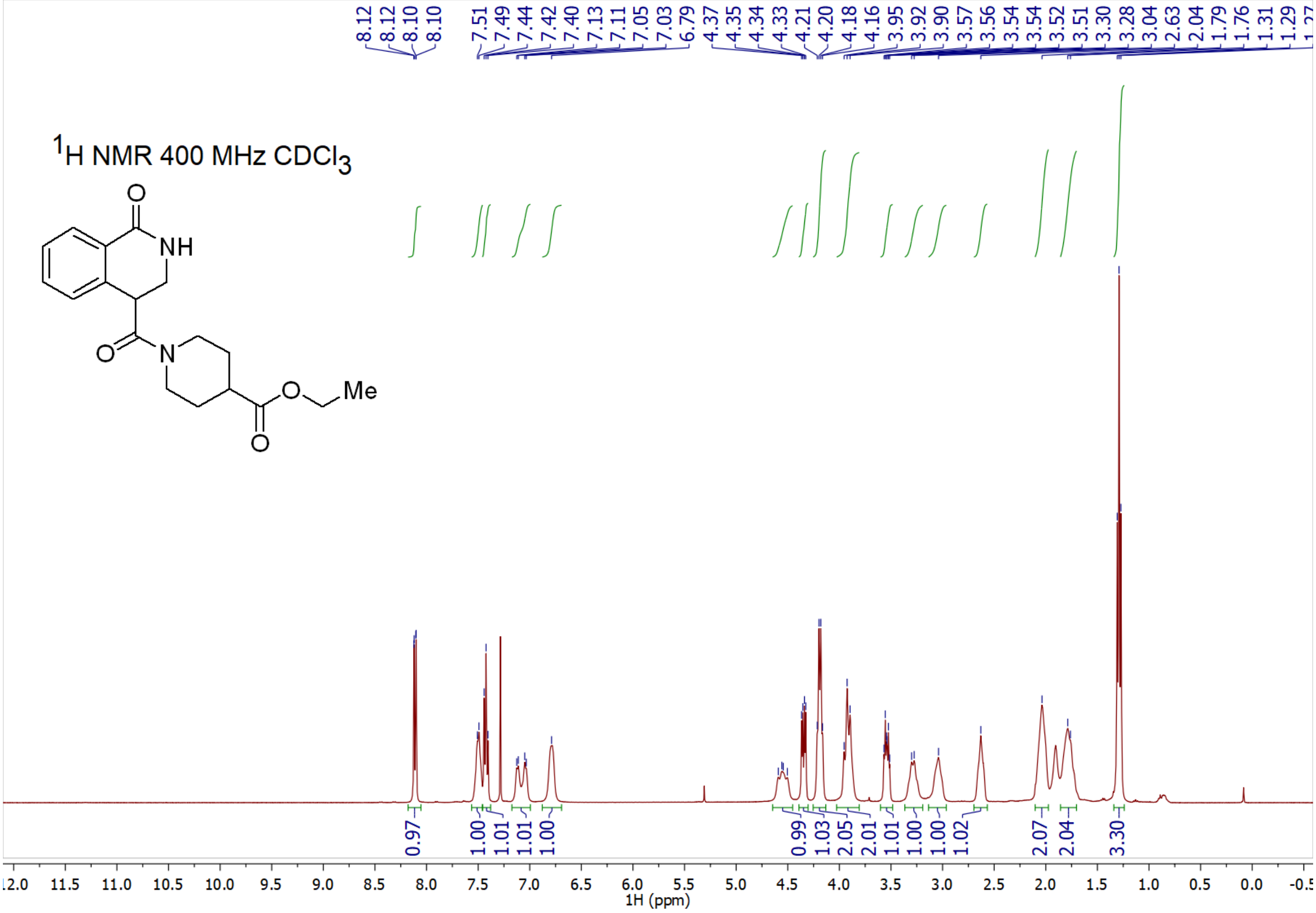

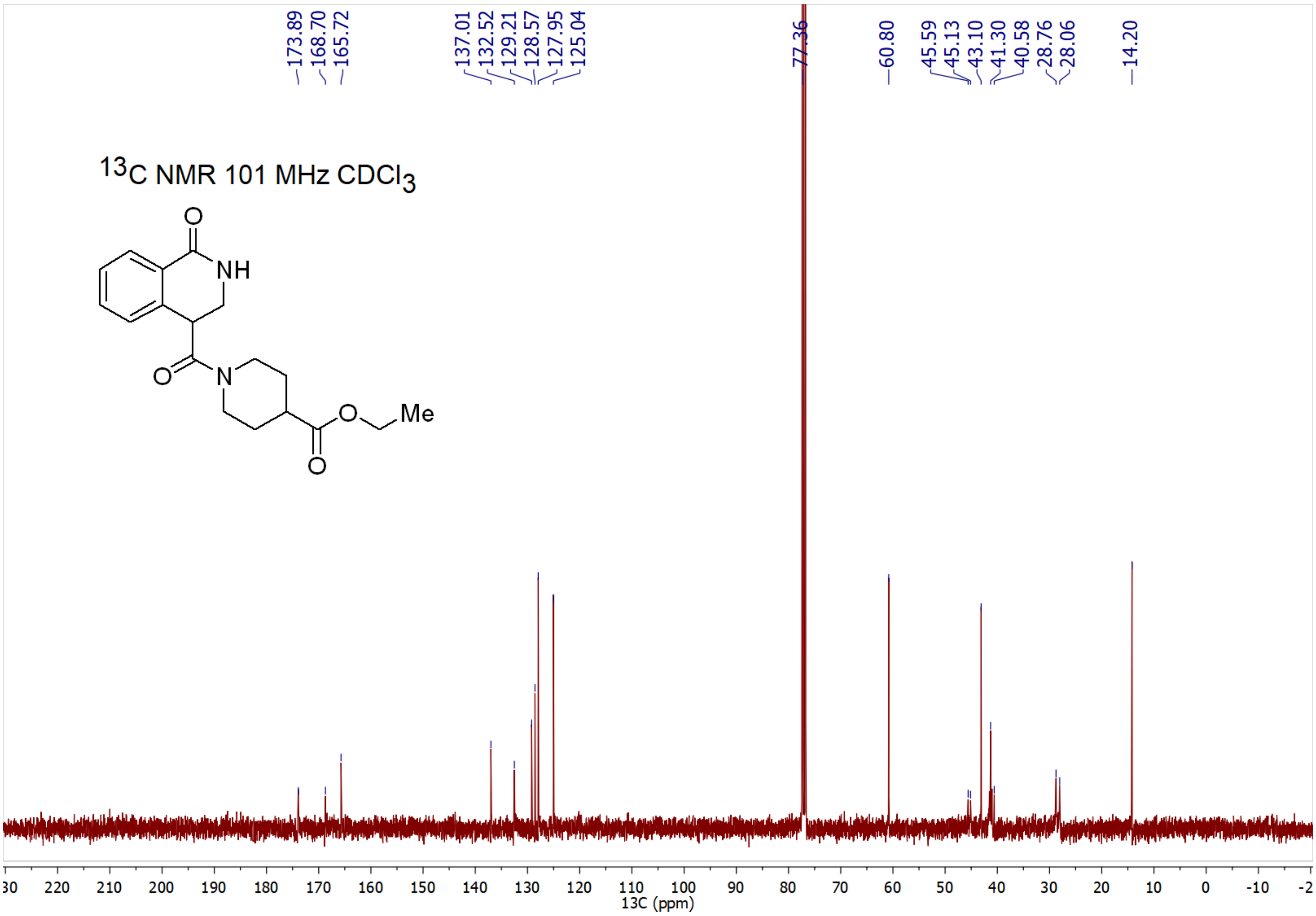

8. ^{1}H and ^{13}C NMR Spectra of **3h**

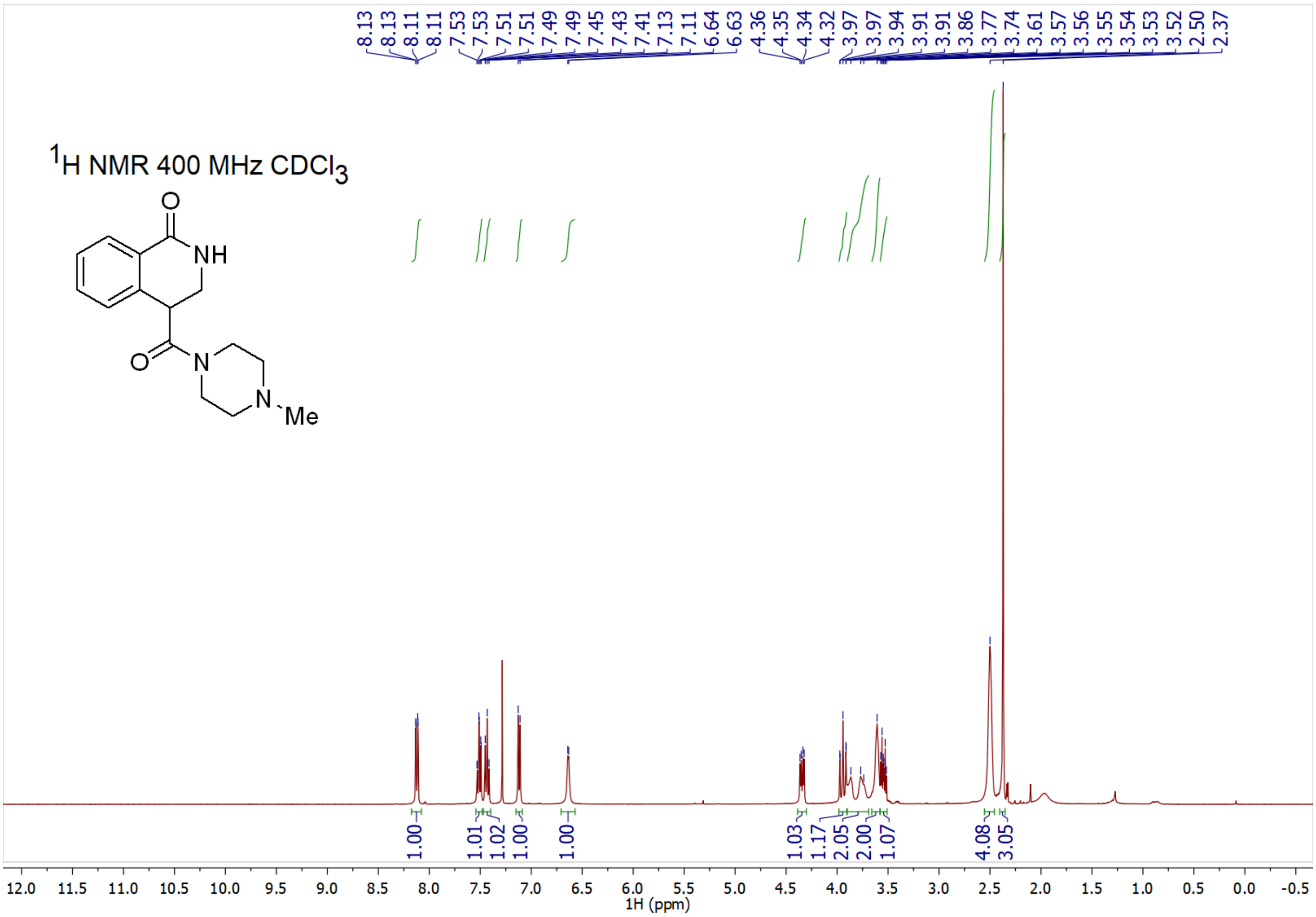

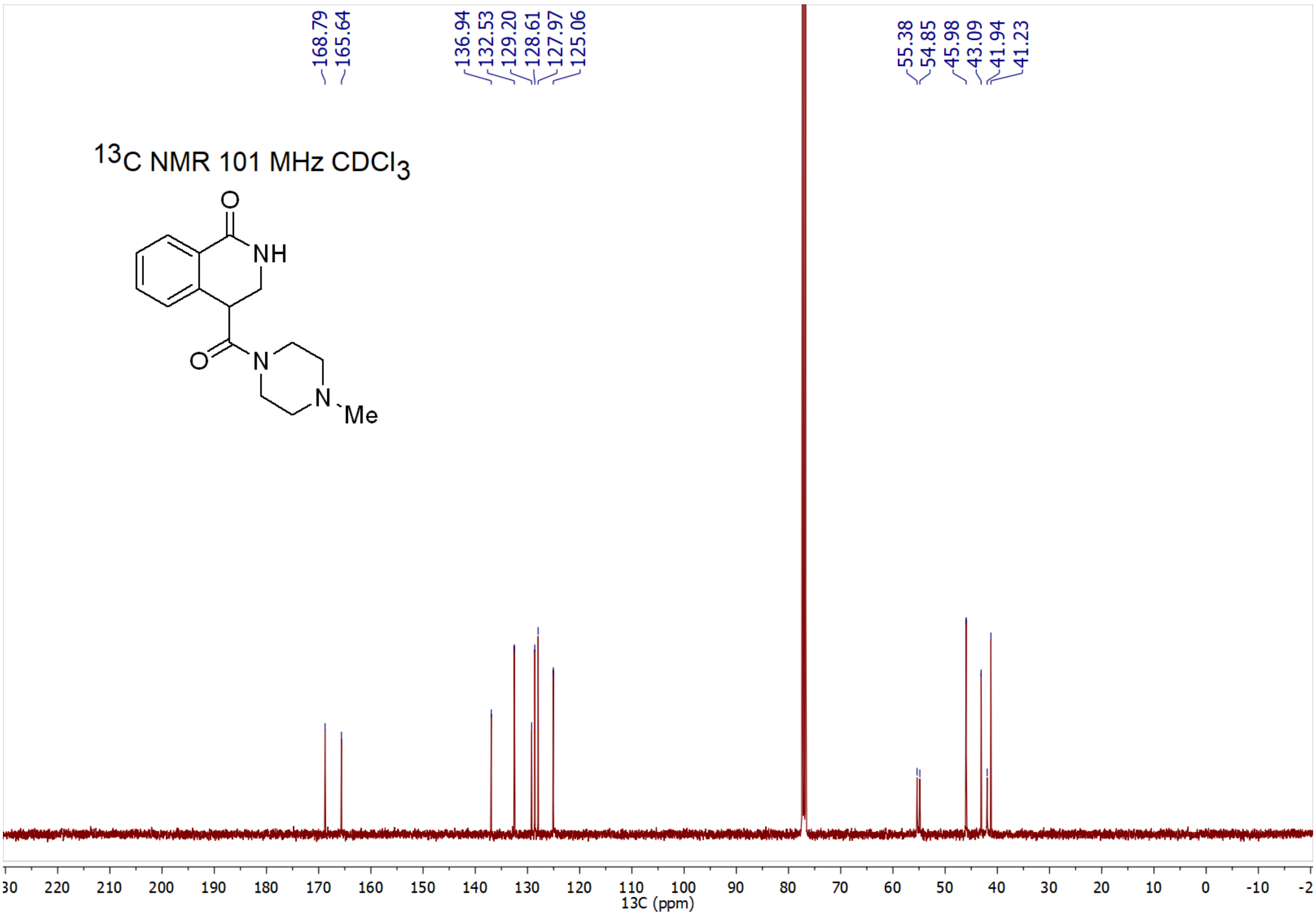

9. ^{1}H and ^{13}C NMR Spectra of **3i**

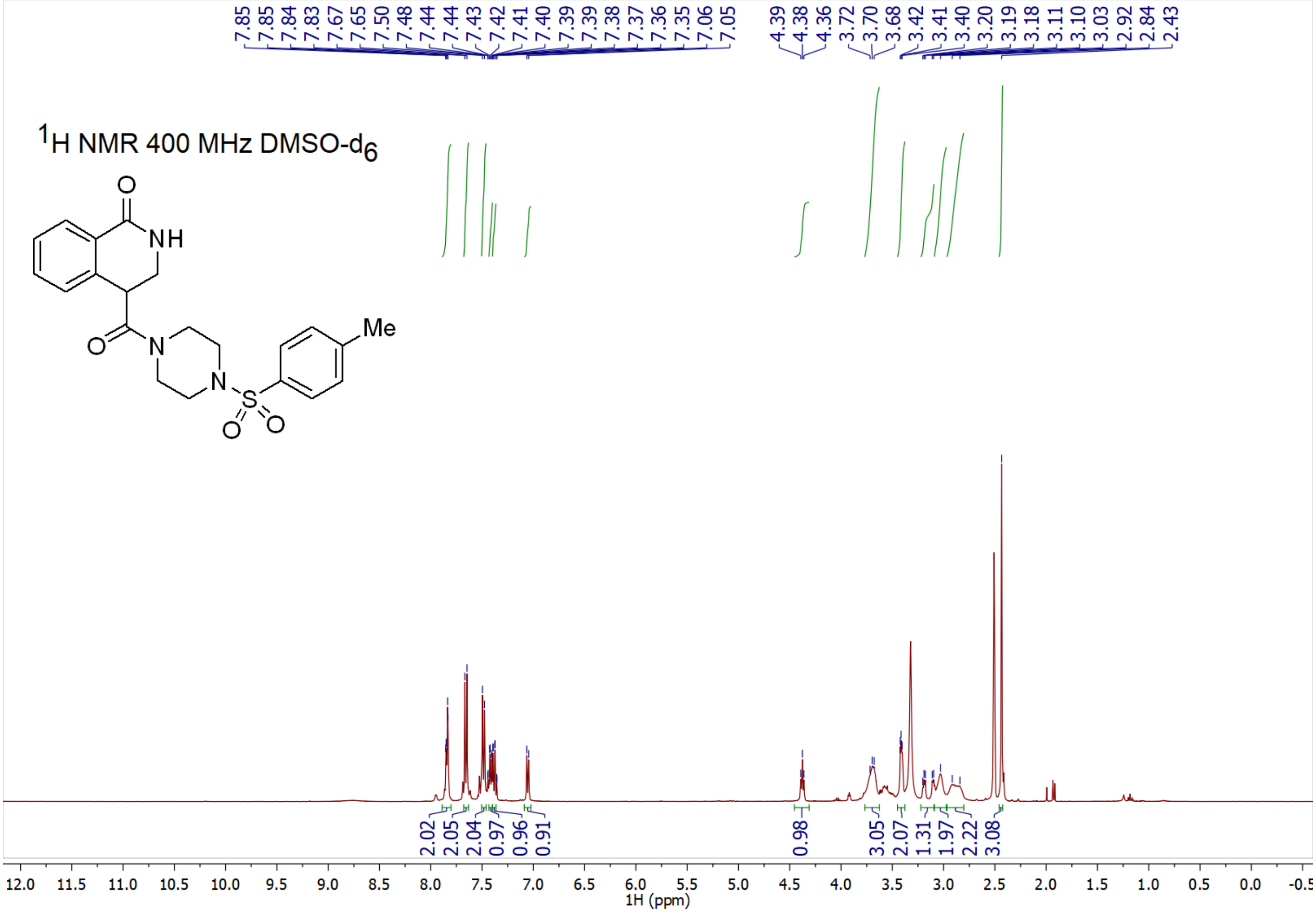

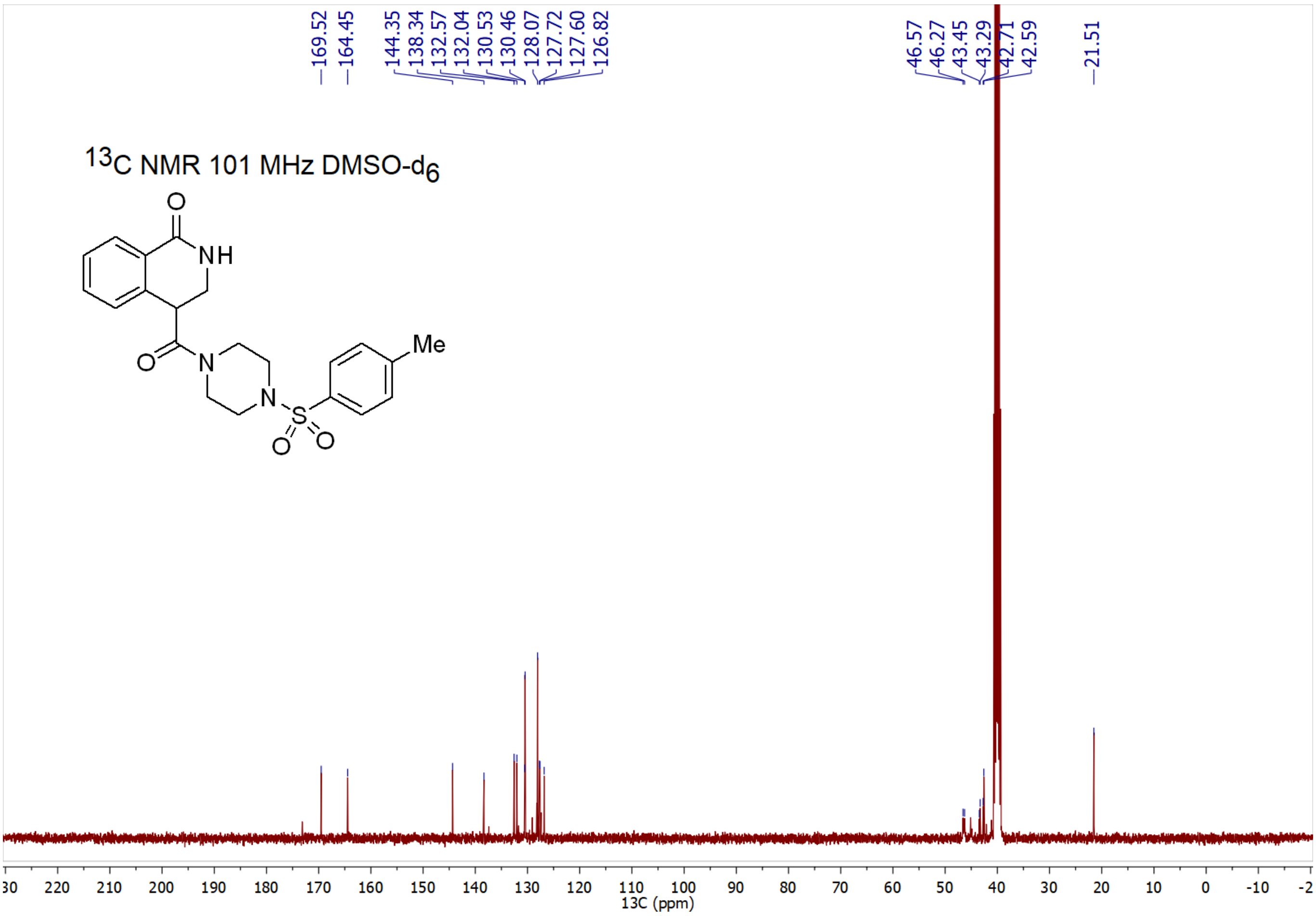

10. ^{1}H and ^{13}C NMR Spectra of **3j**

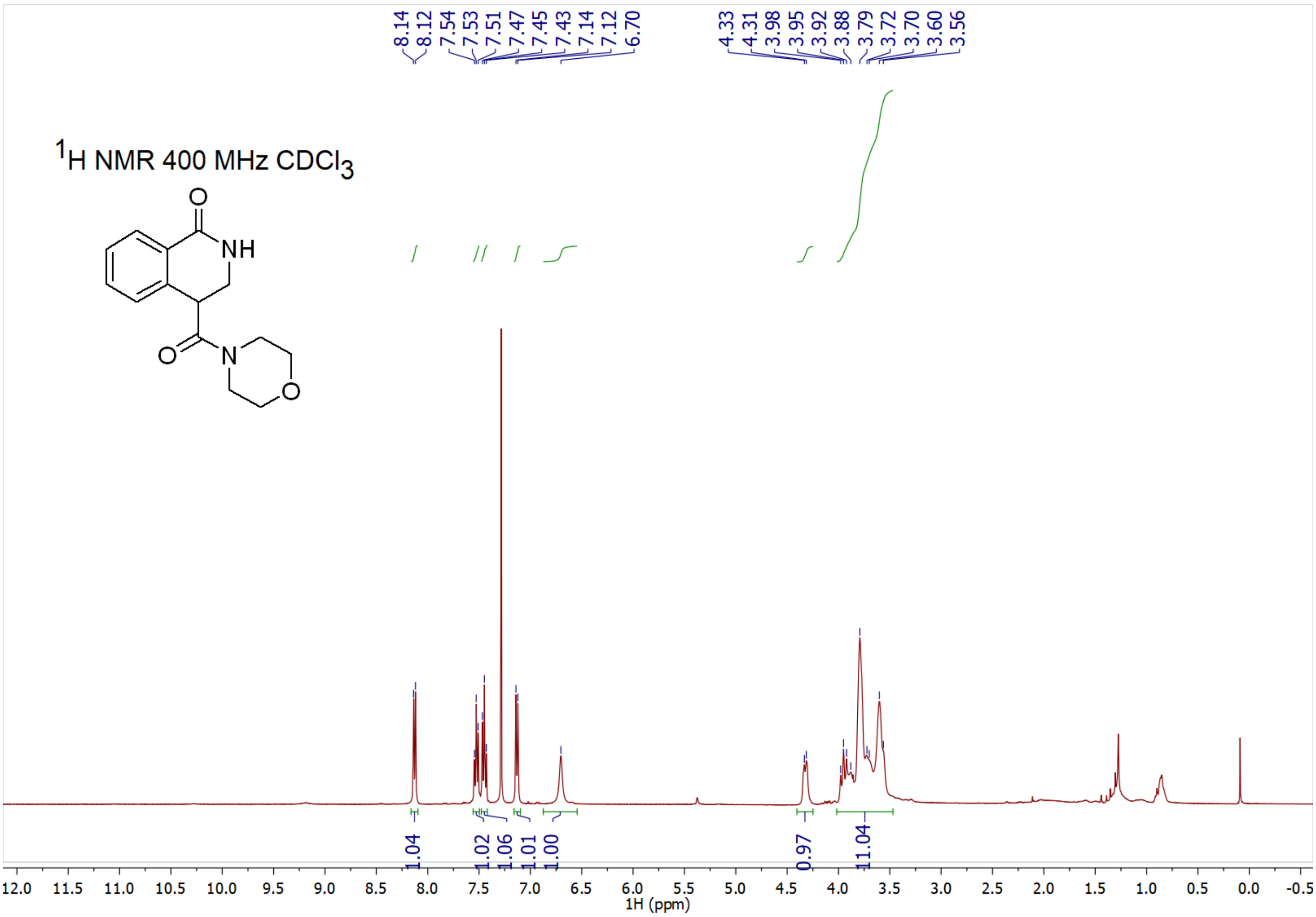

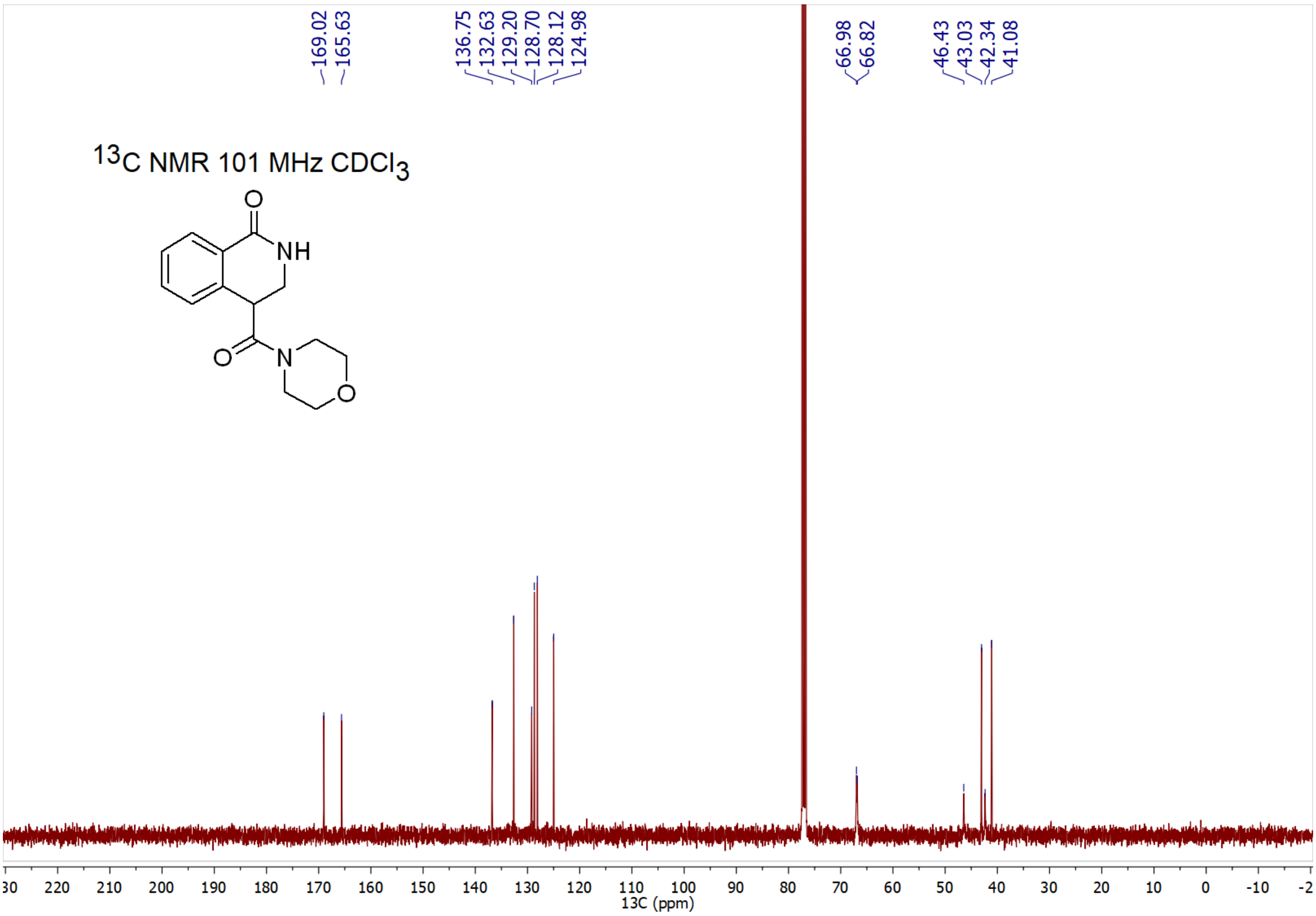

11. ^{1}H and ^{13}C NMR Spectra of **3k**

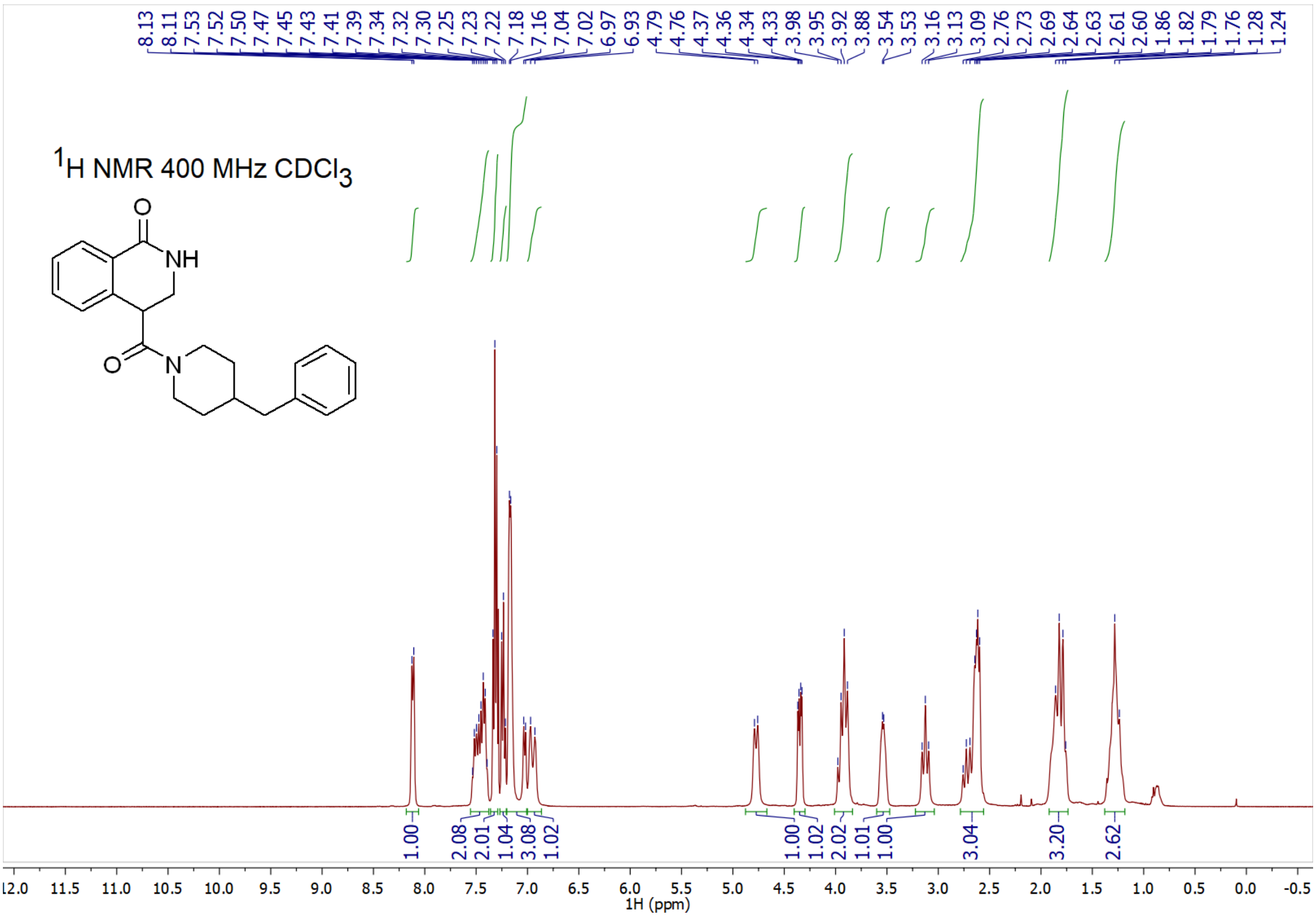

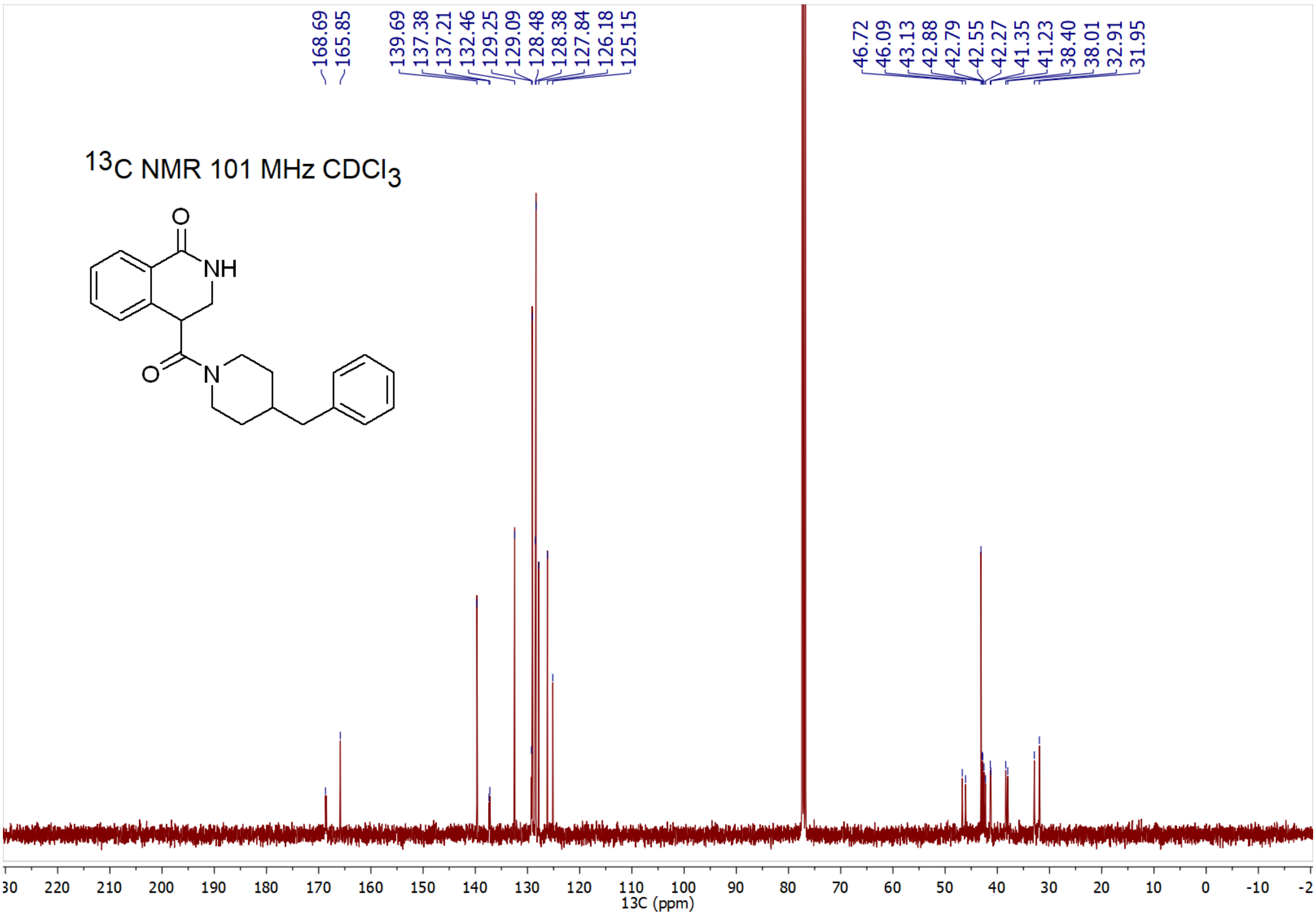

12. ^{1}H and ^{13}C NMR Spectra of **3l**

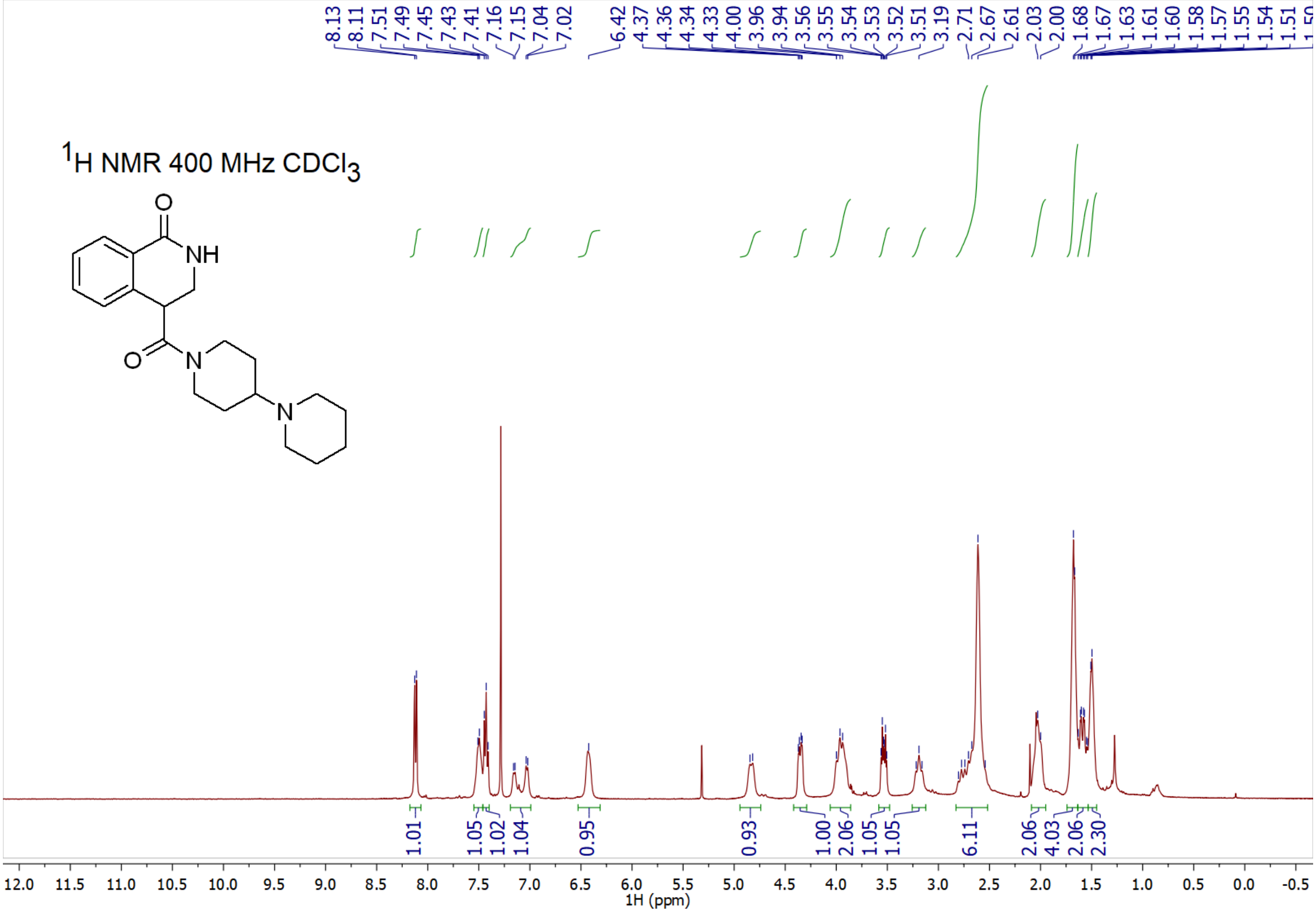

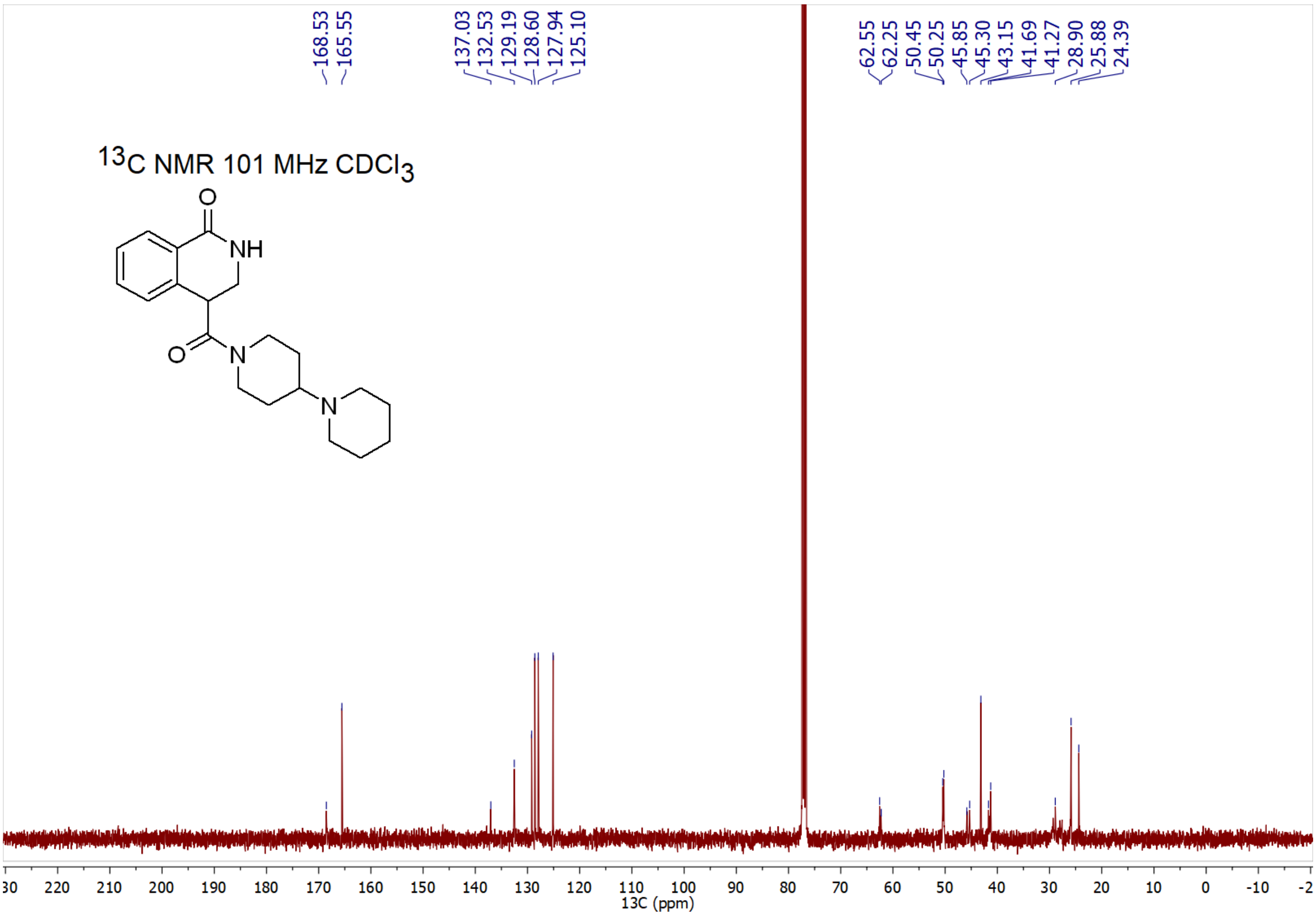

13. ^{1}H and ^{13}C NMR Spectra of **3m**

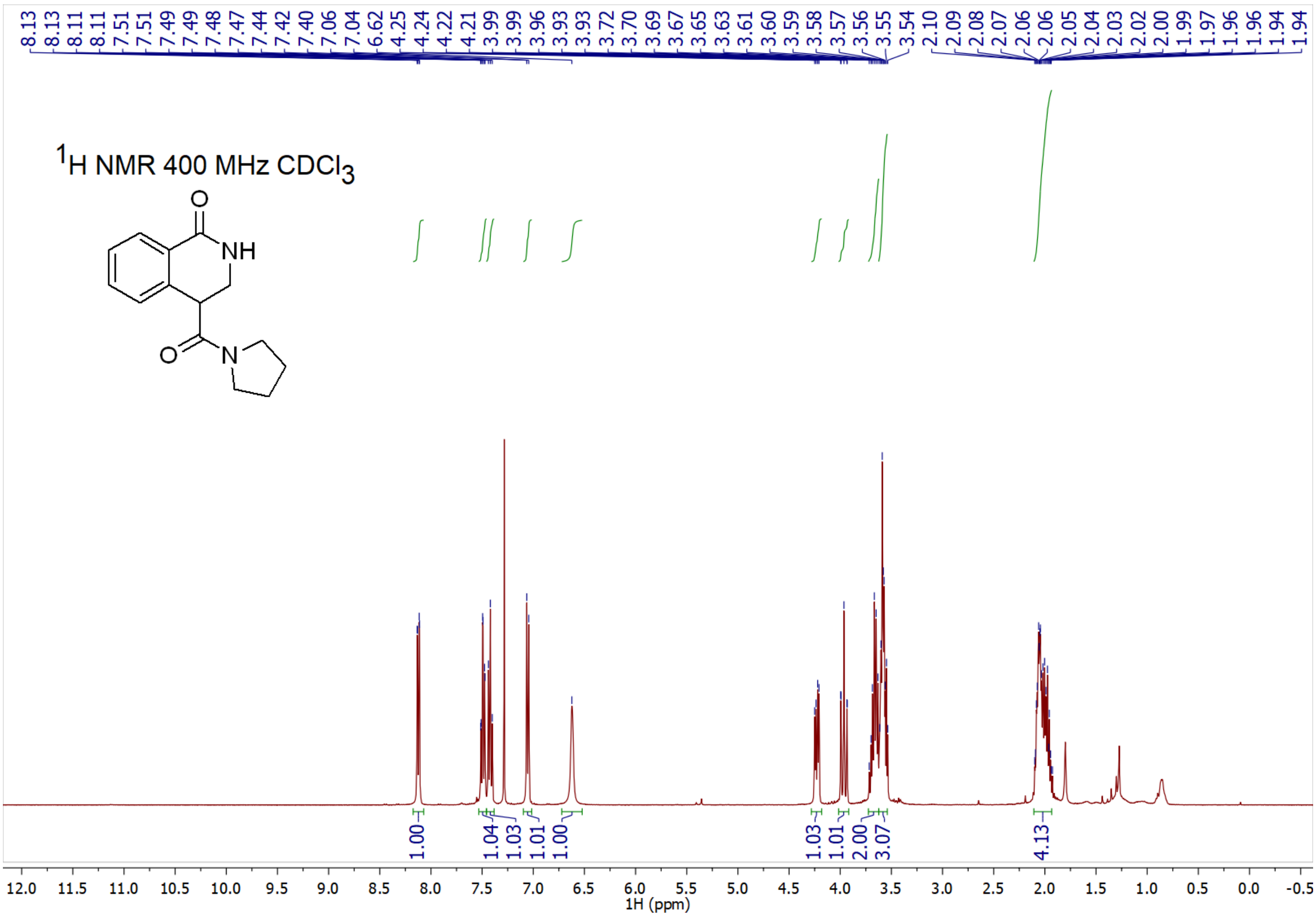

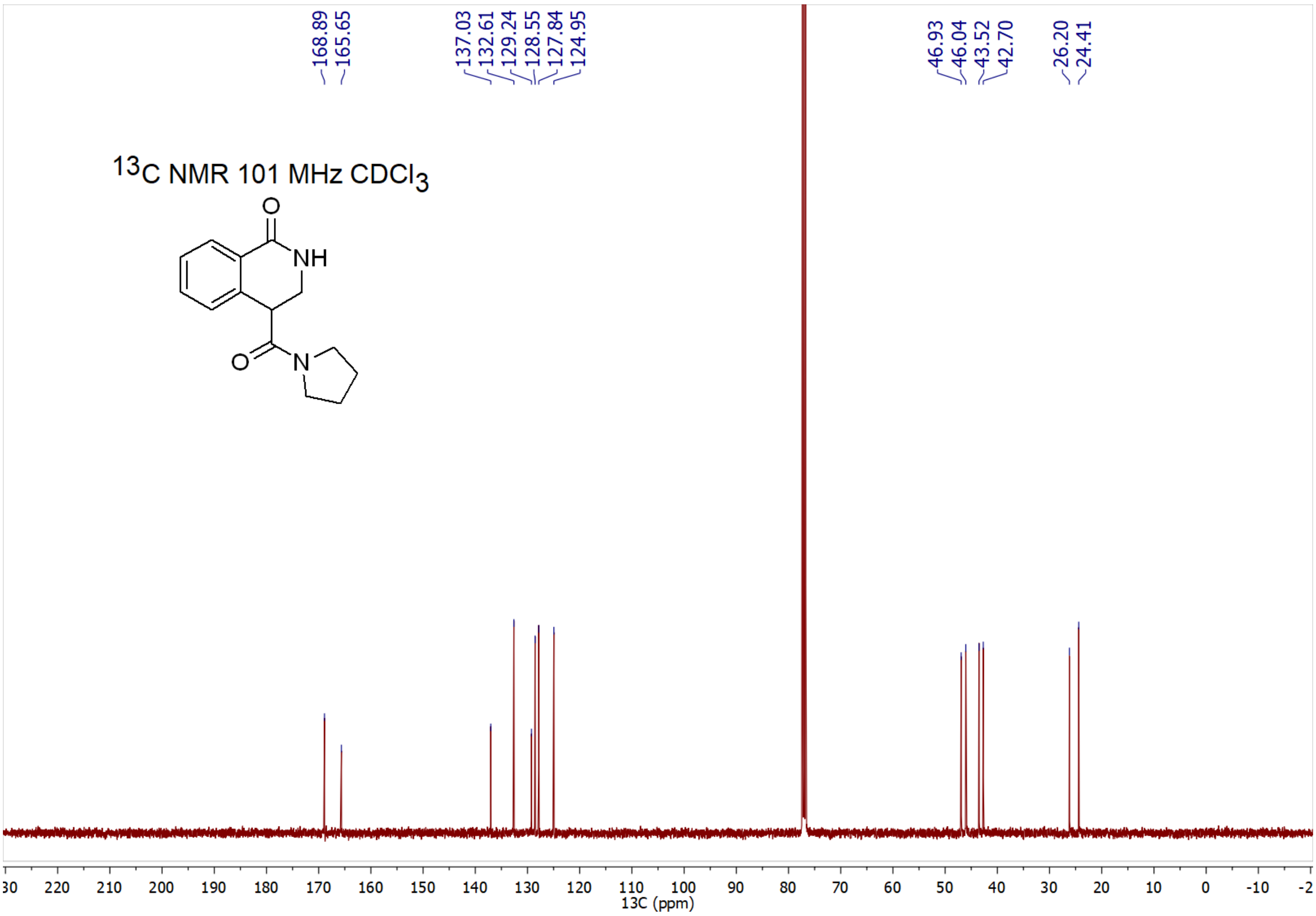

14. ^{1}H and ^{13}C NMR Spectra of **3n**

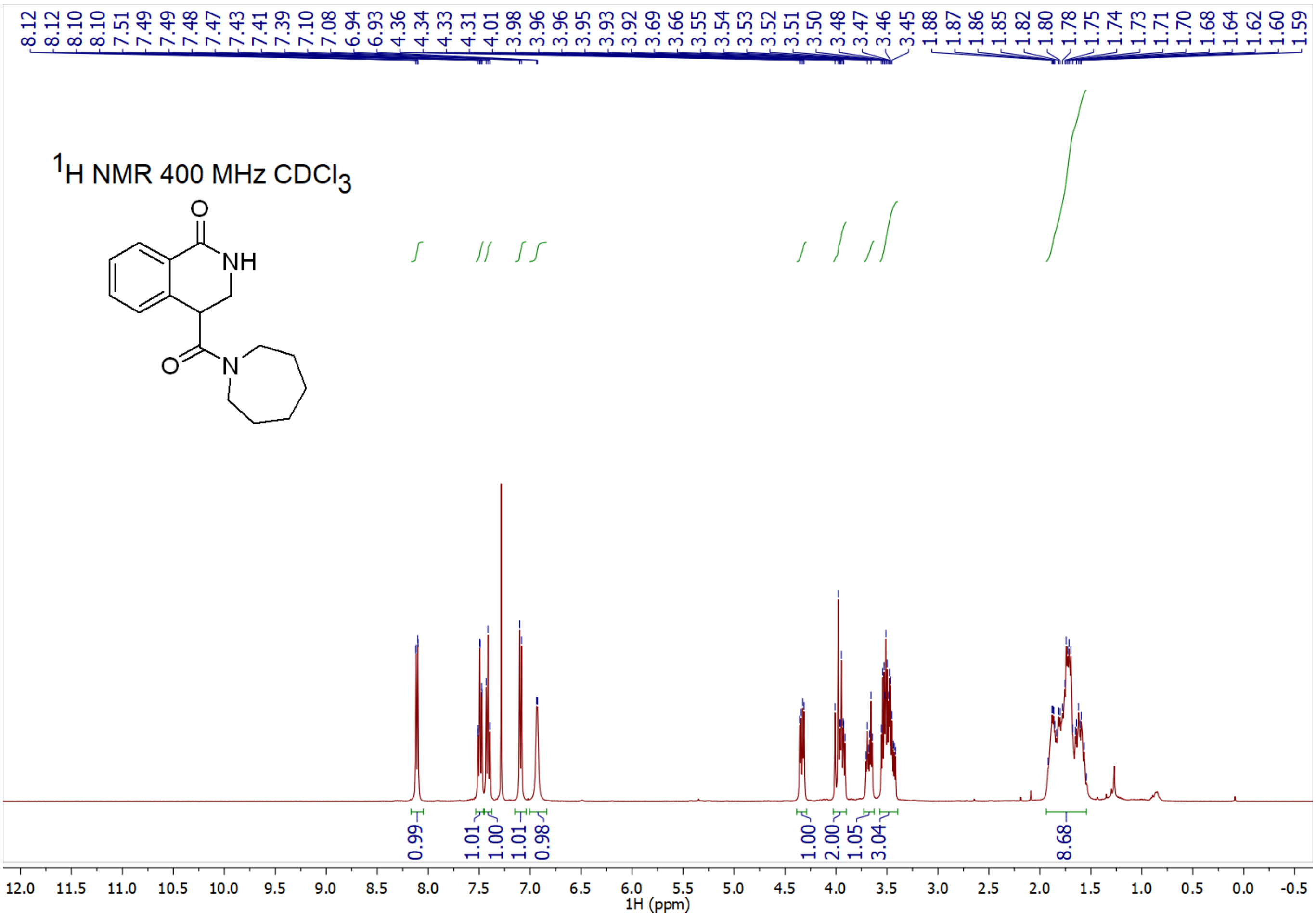

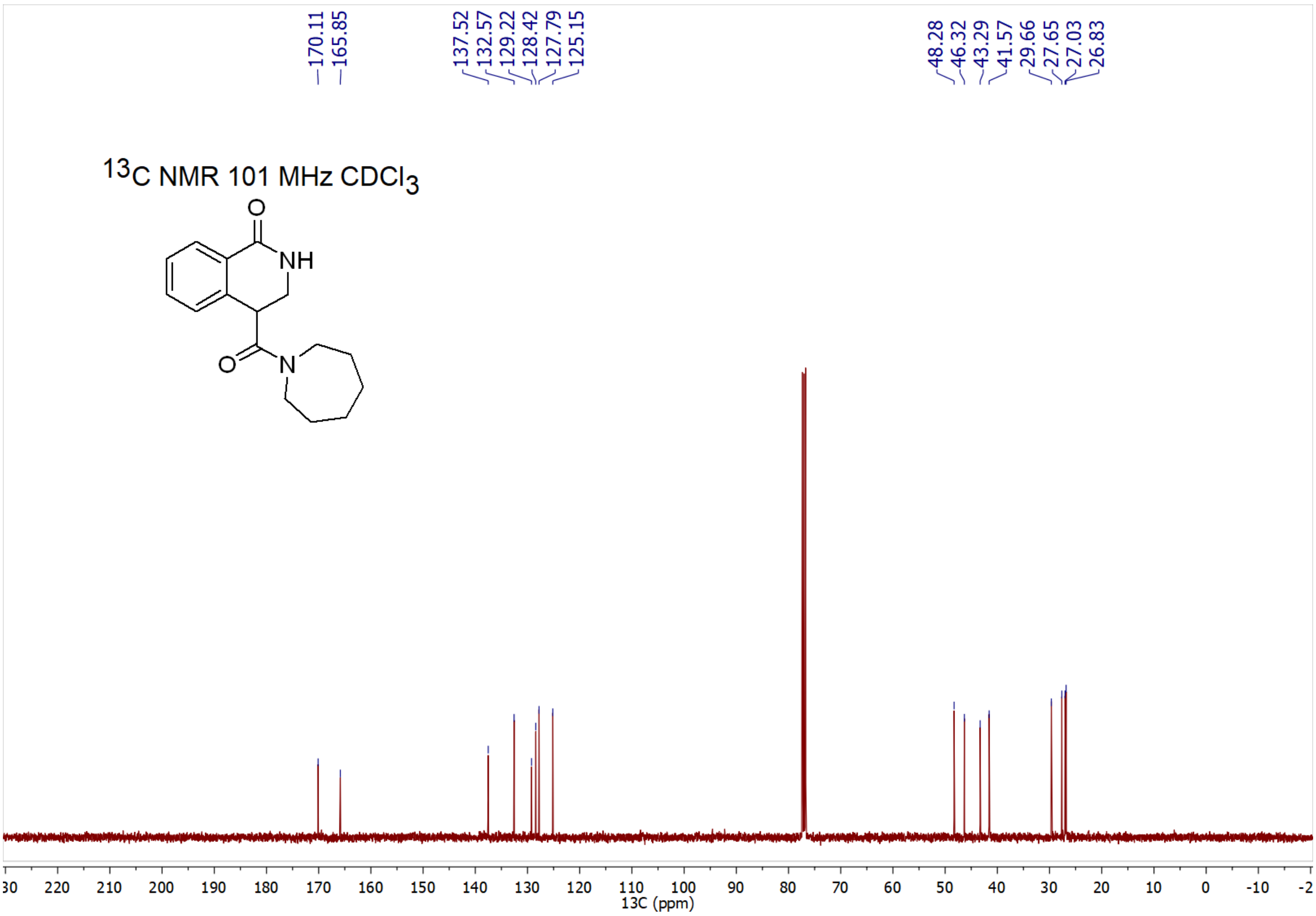

15. ^{1}H and ^{13}C NMR Spectra of **3o**

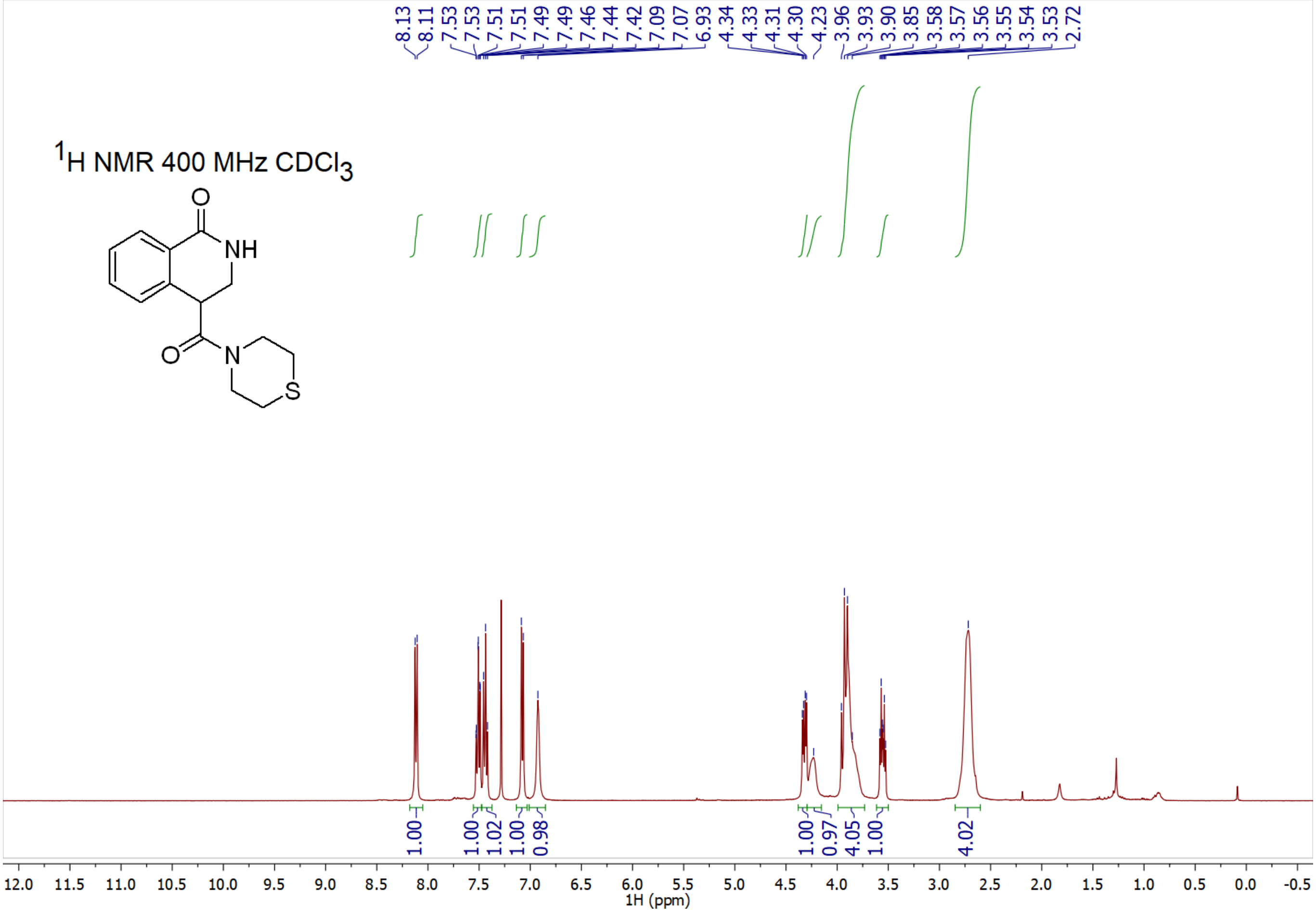

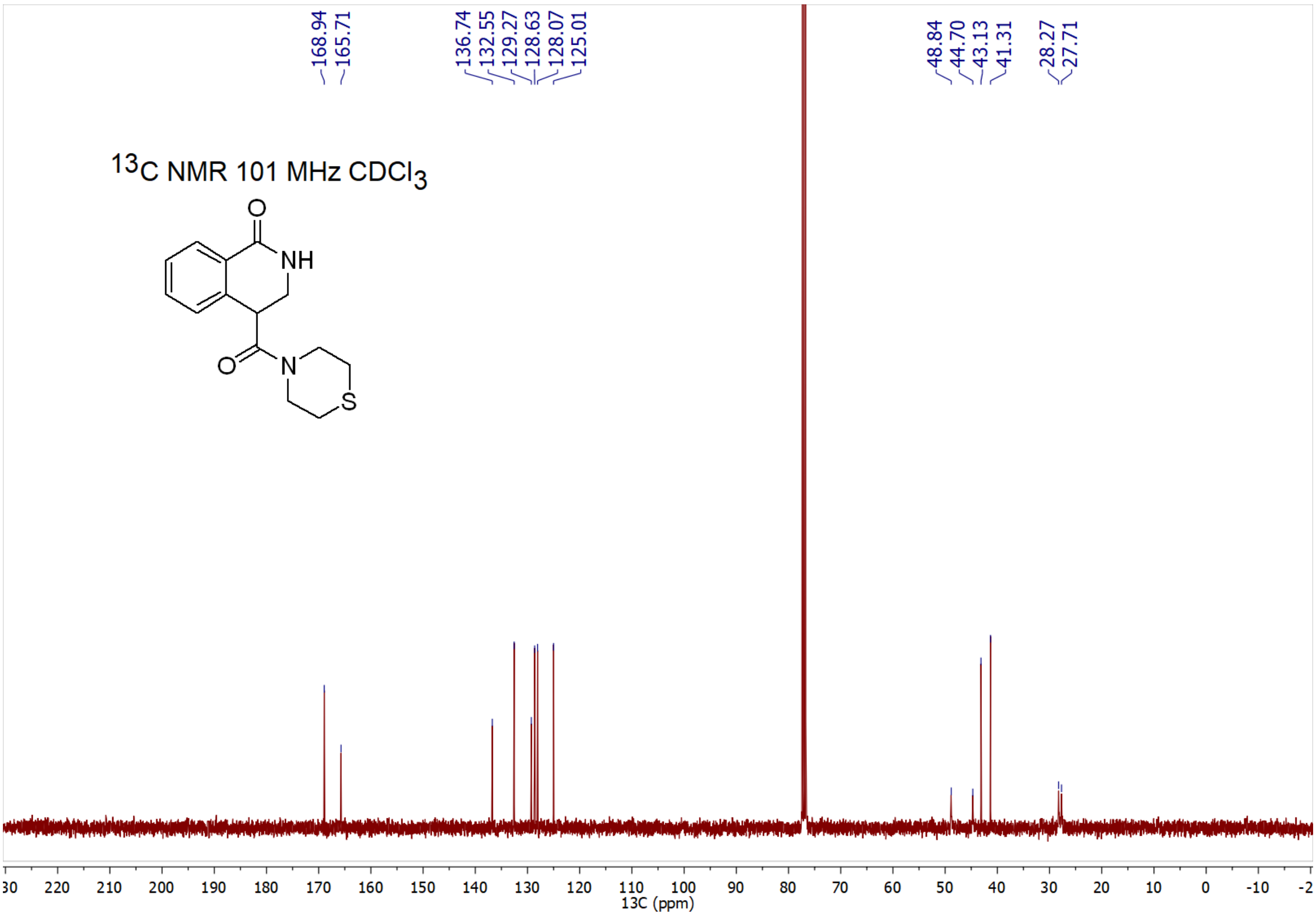

16. ^{1}H and ^{13}C NMR Spectra of **3p**

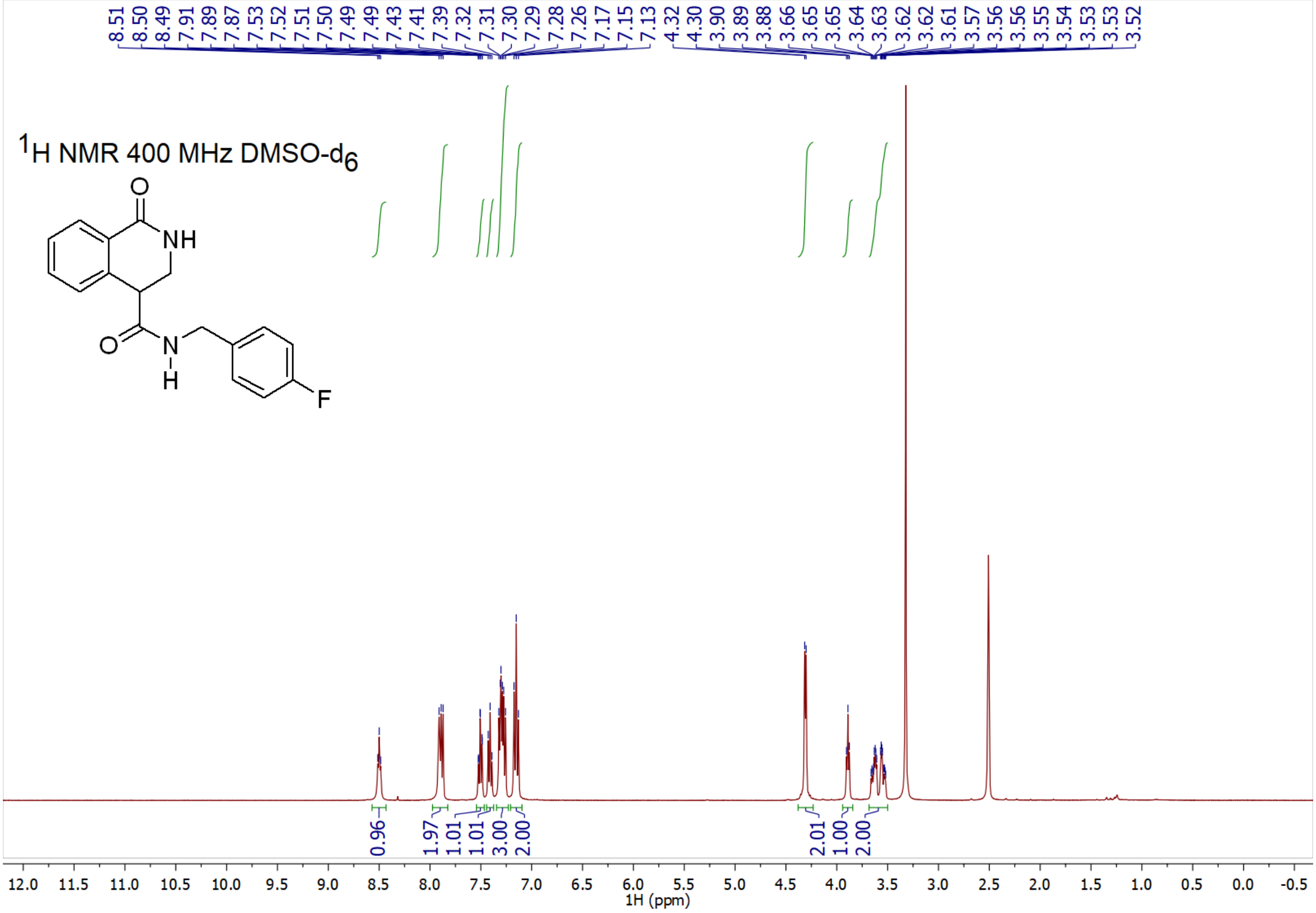

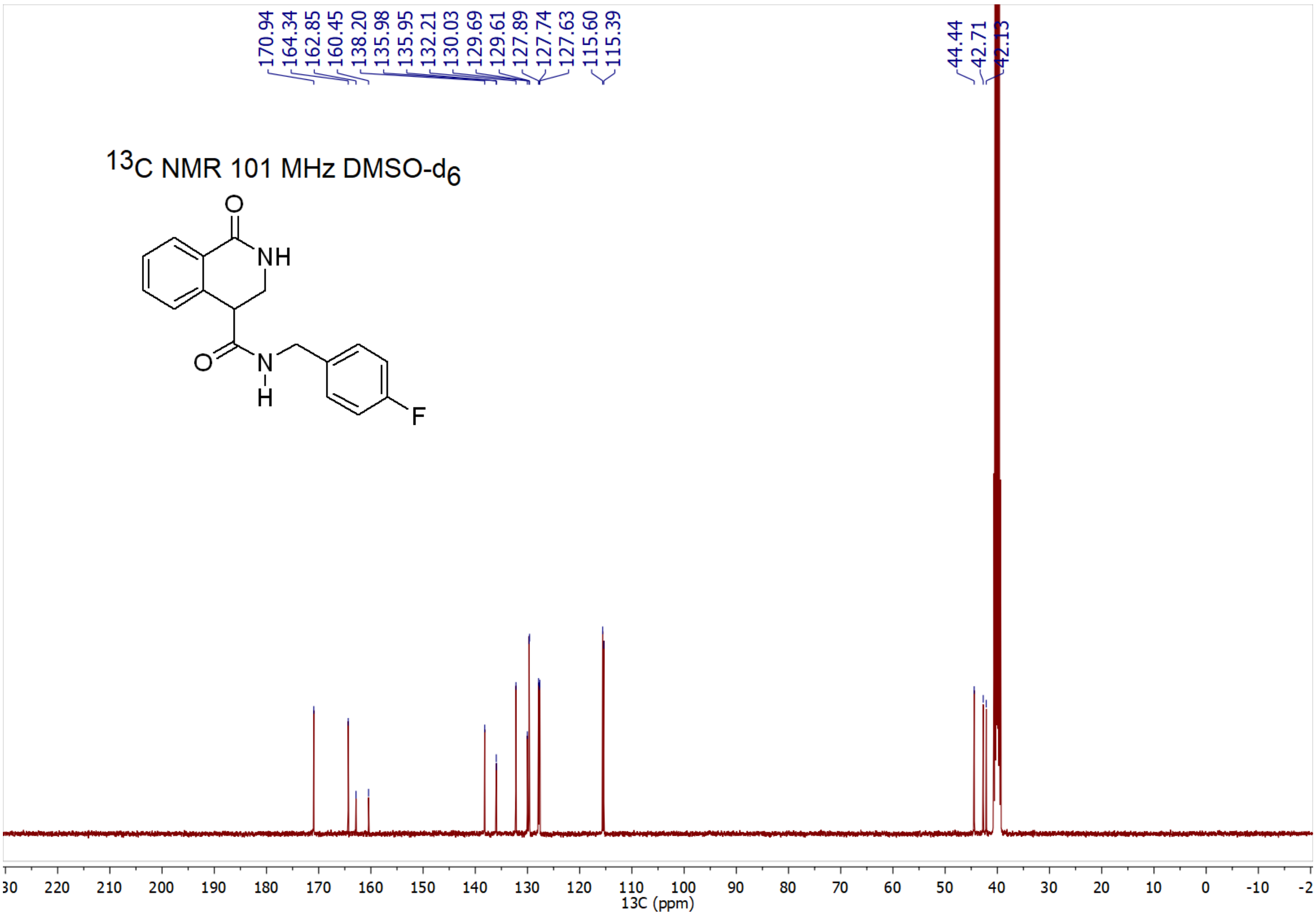

17. ^{1}H and ^{13}C NMR Spectra of **3q**

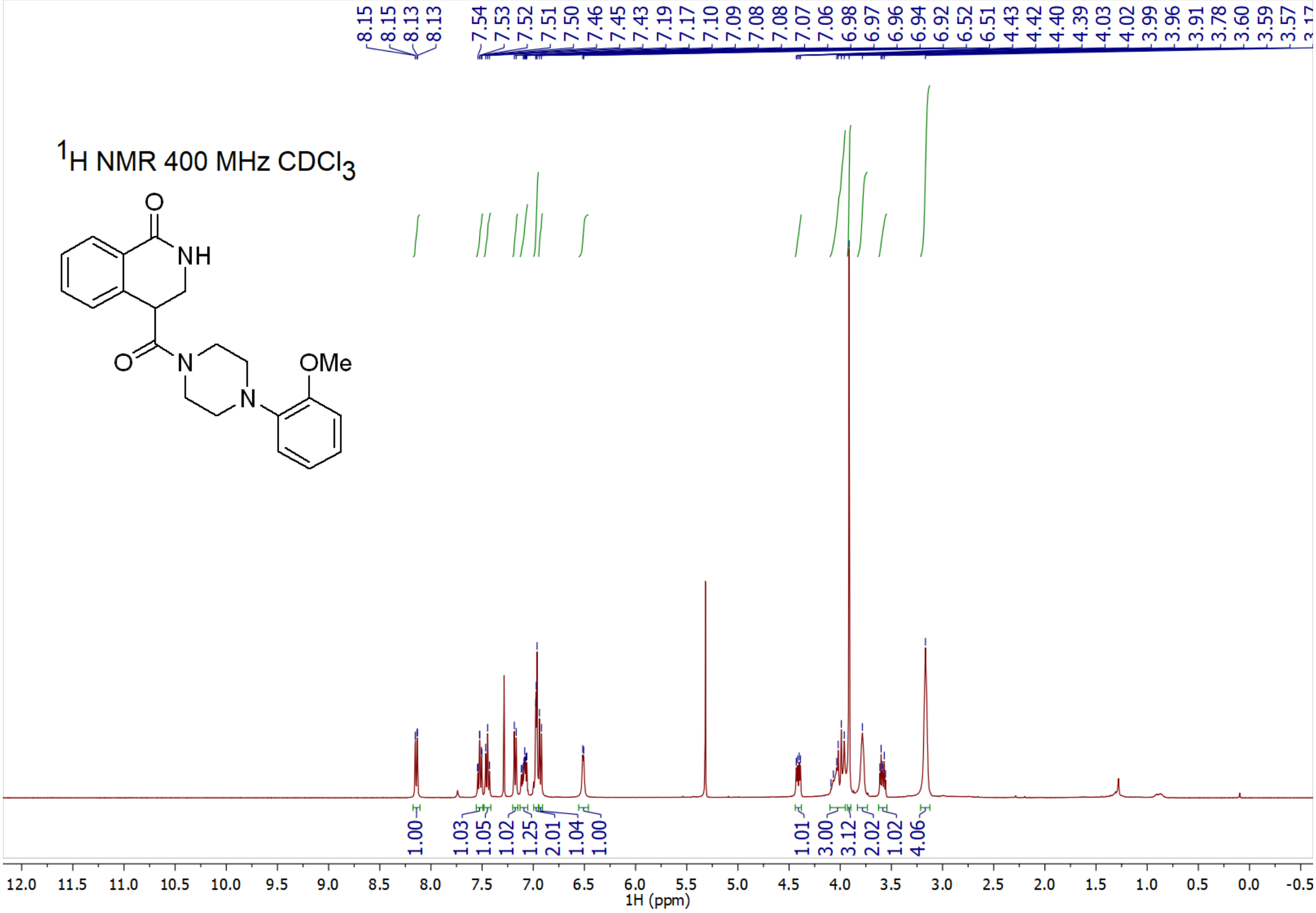

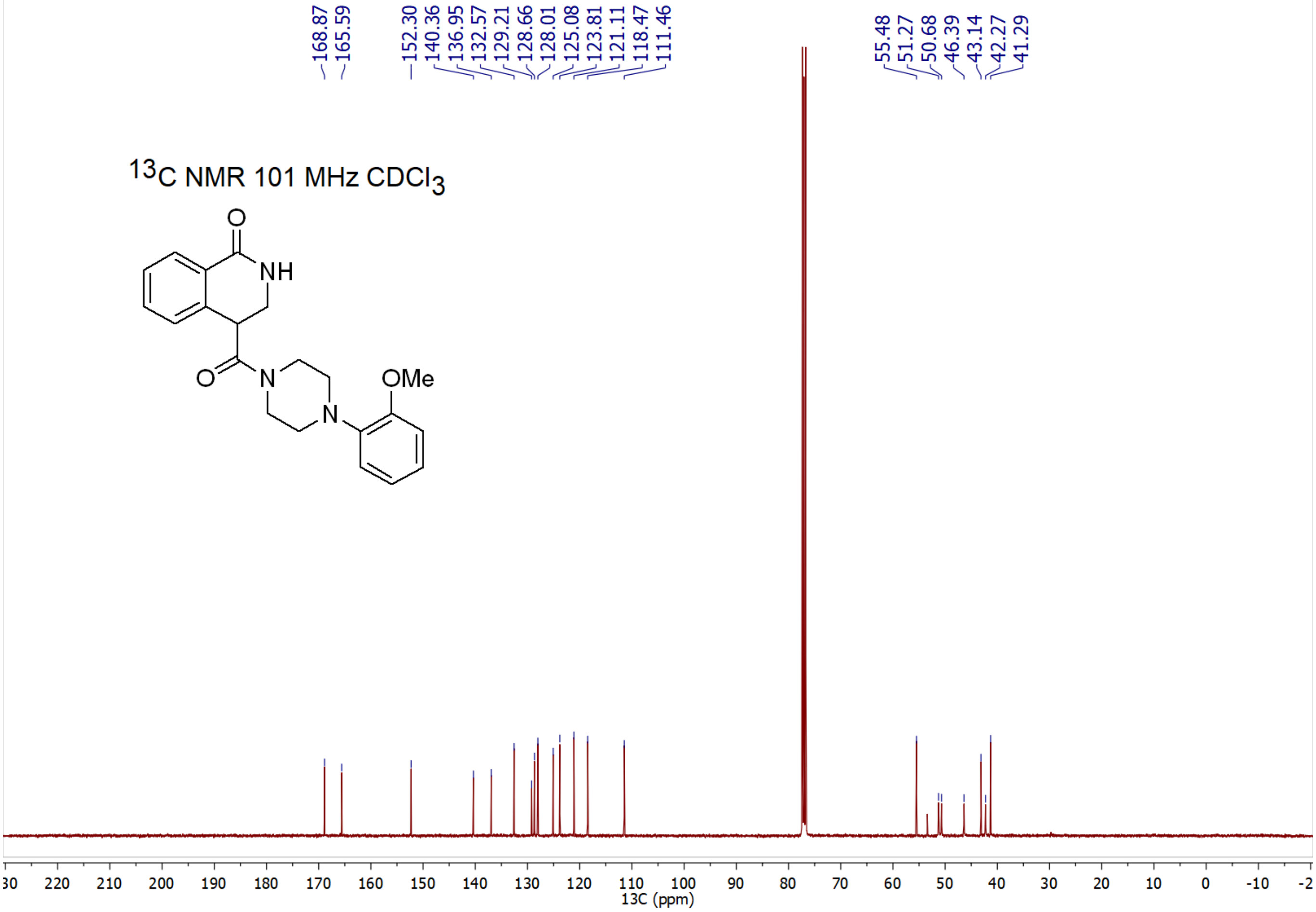

18. ^{1}H and ^{13}C NMR Spectra of **3r**

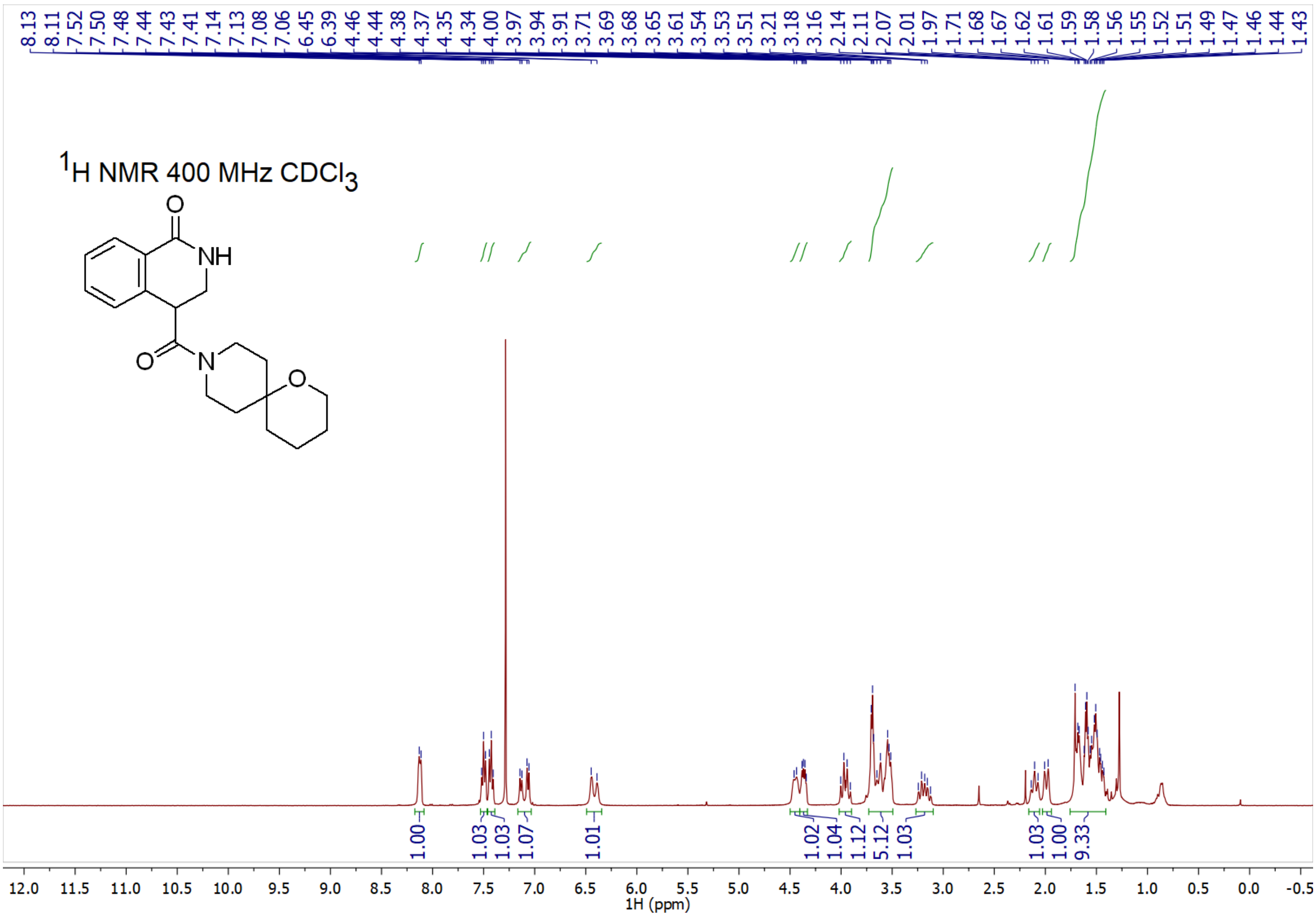

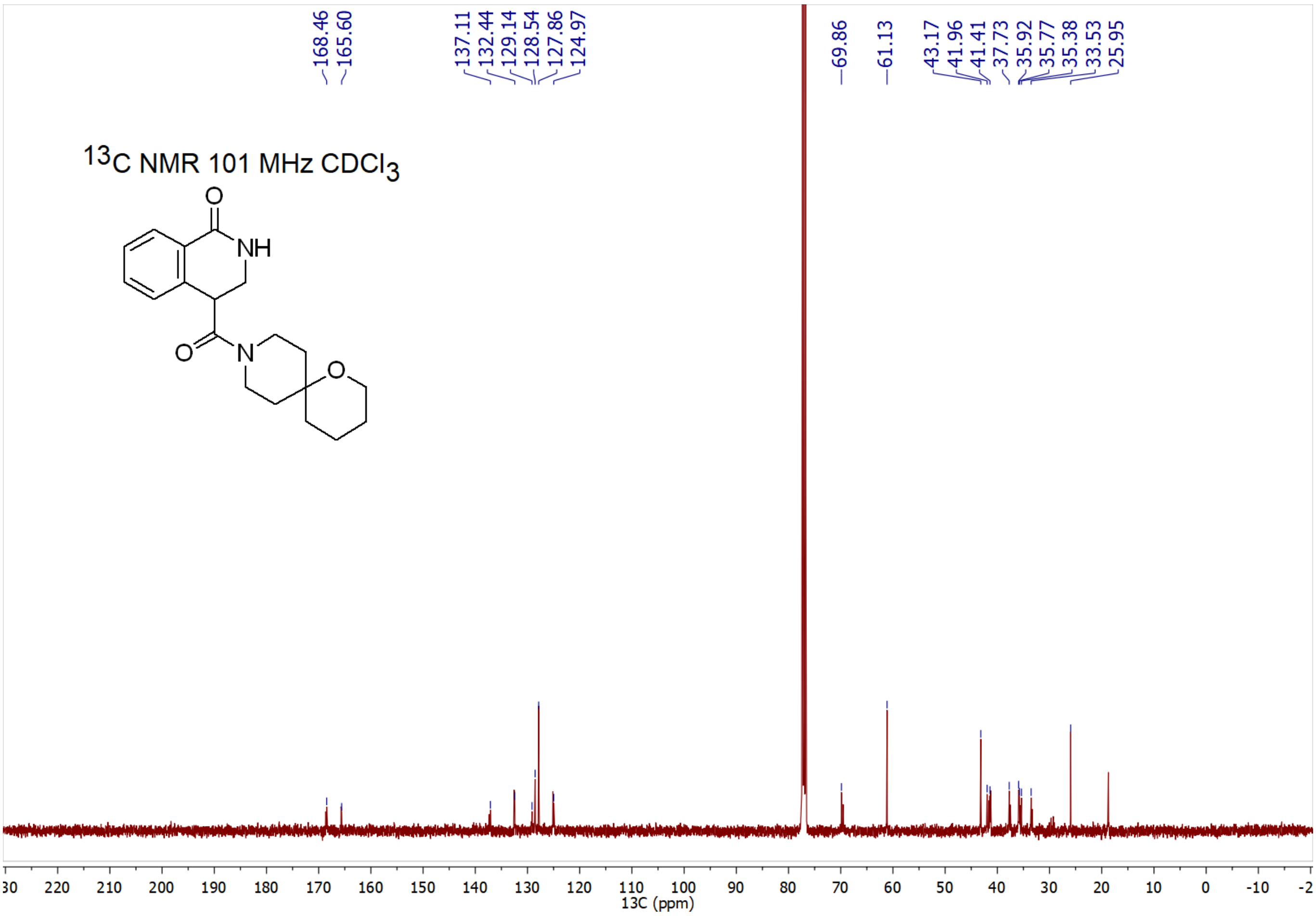

19. ^{1}H and ^{13}C NMR Spectra of **3s**

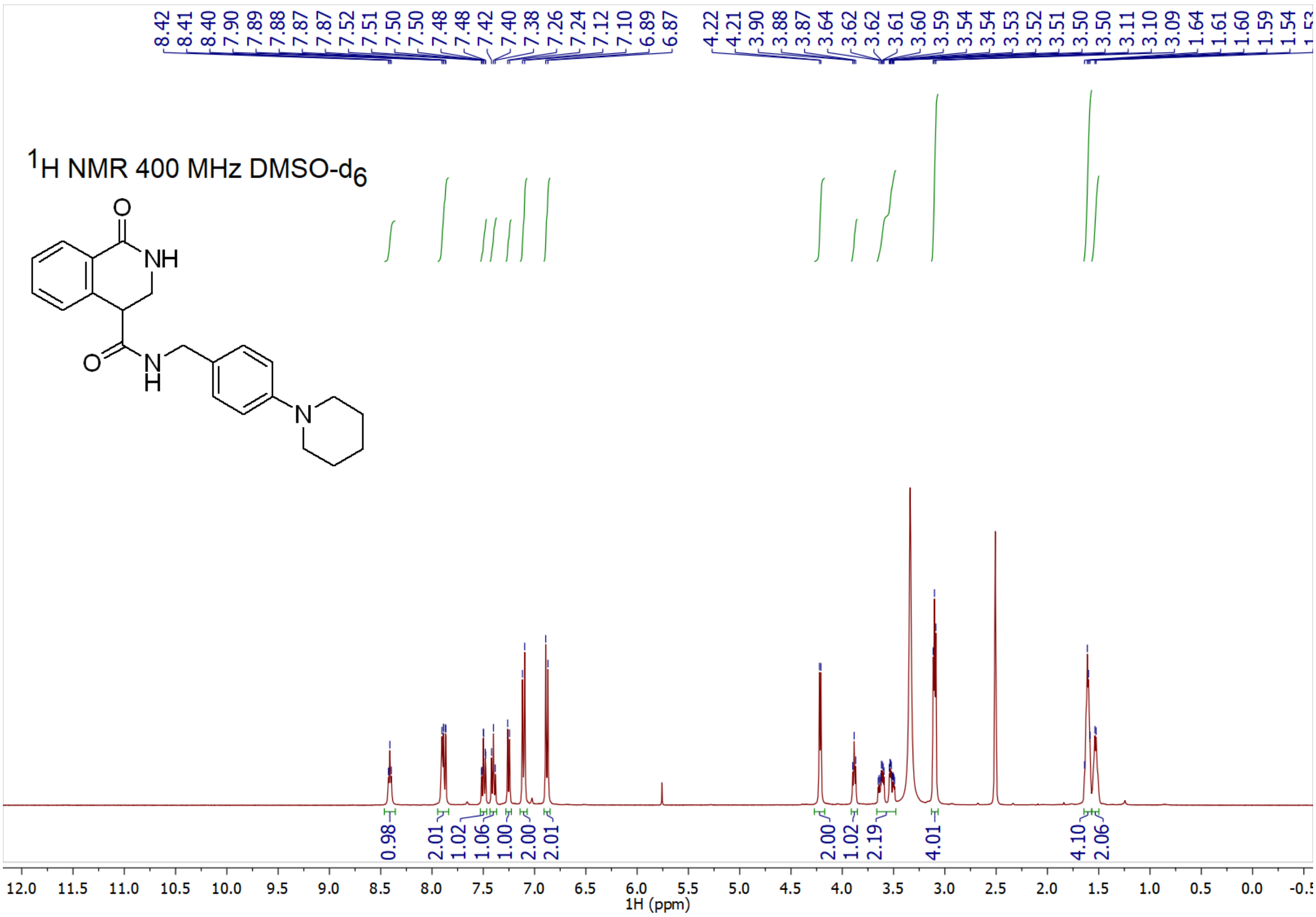

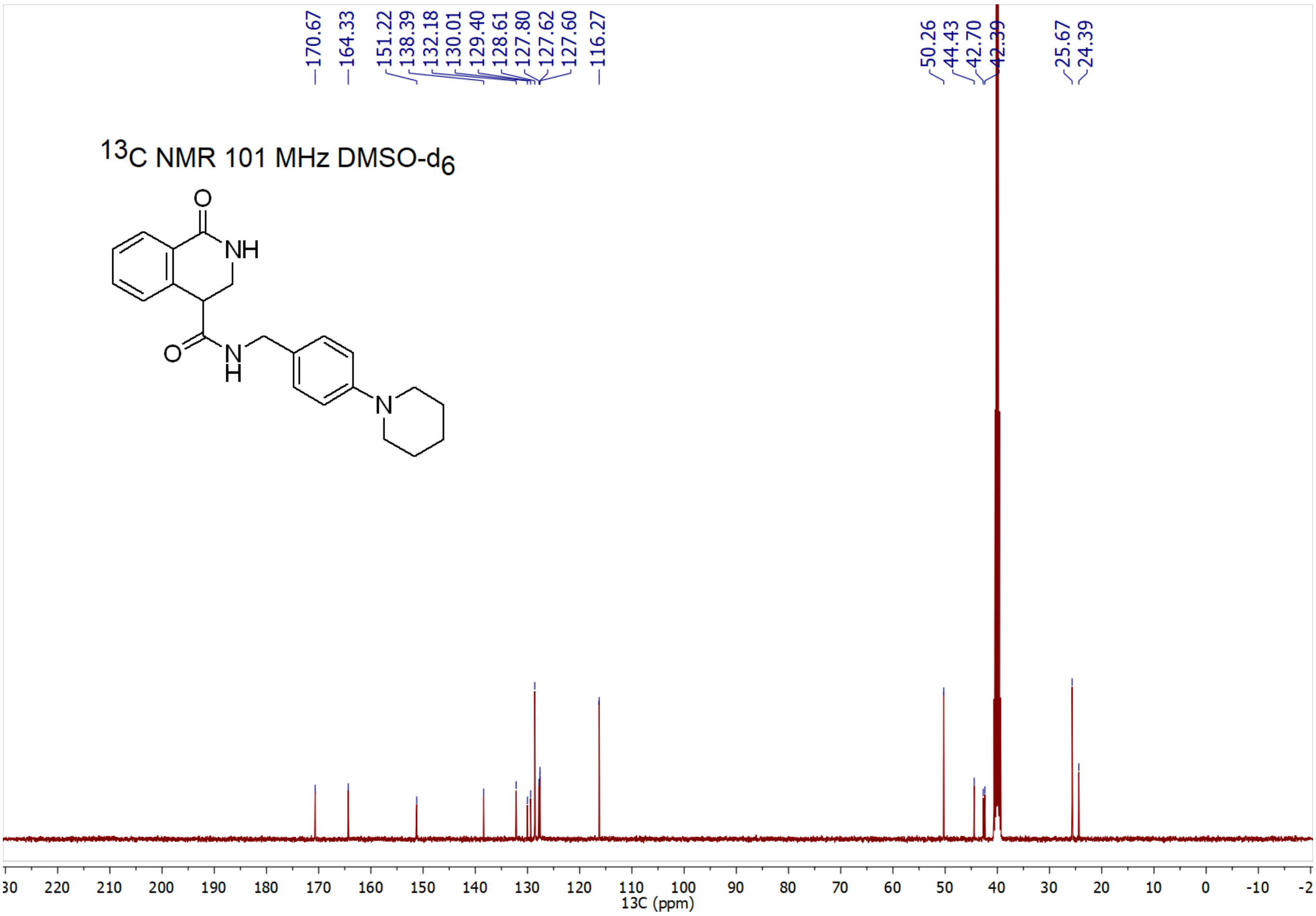

20. ^{1}H and ^{13}C NMR Spectra of **3t**

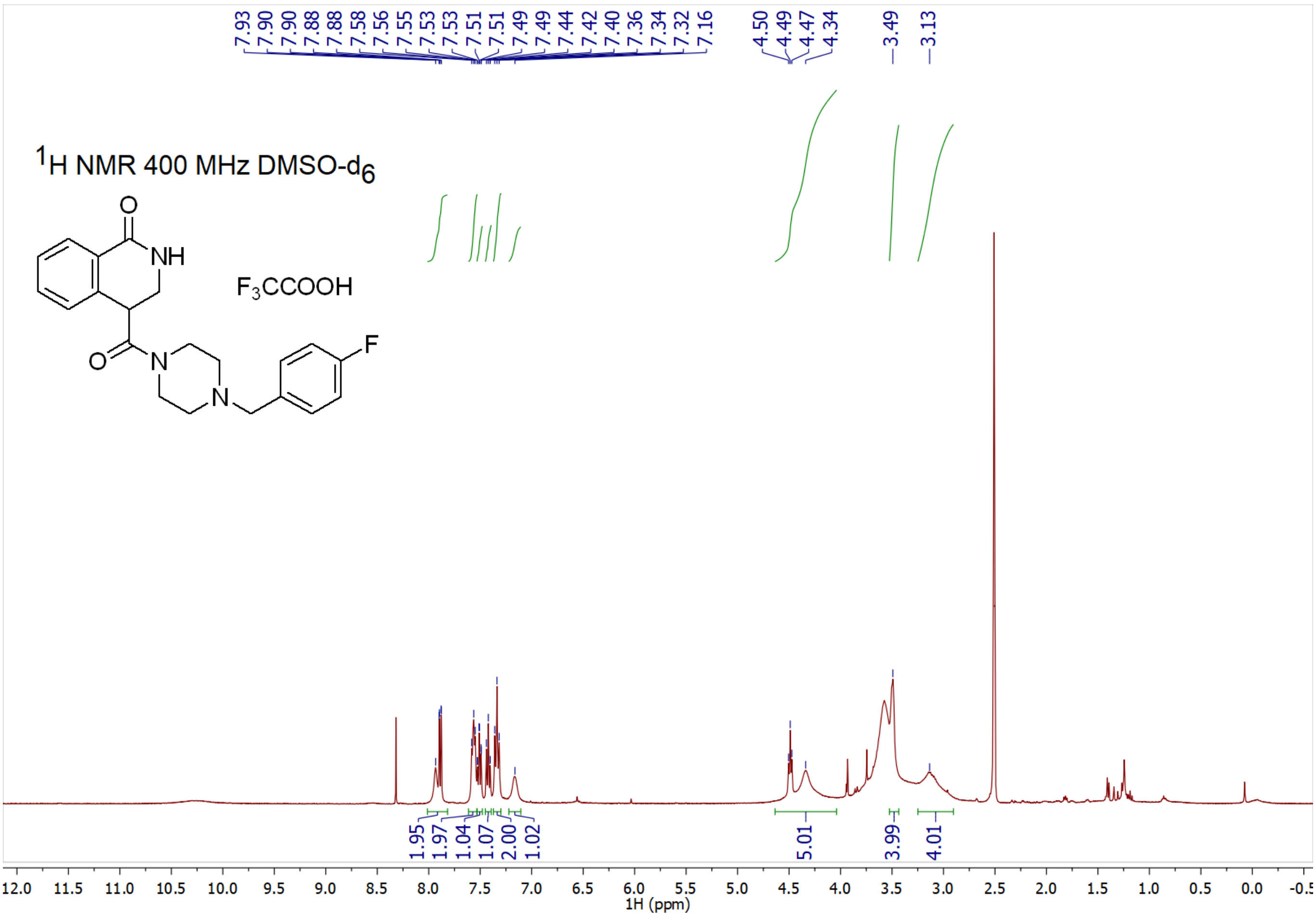

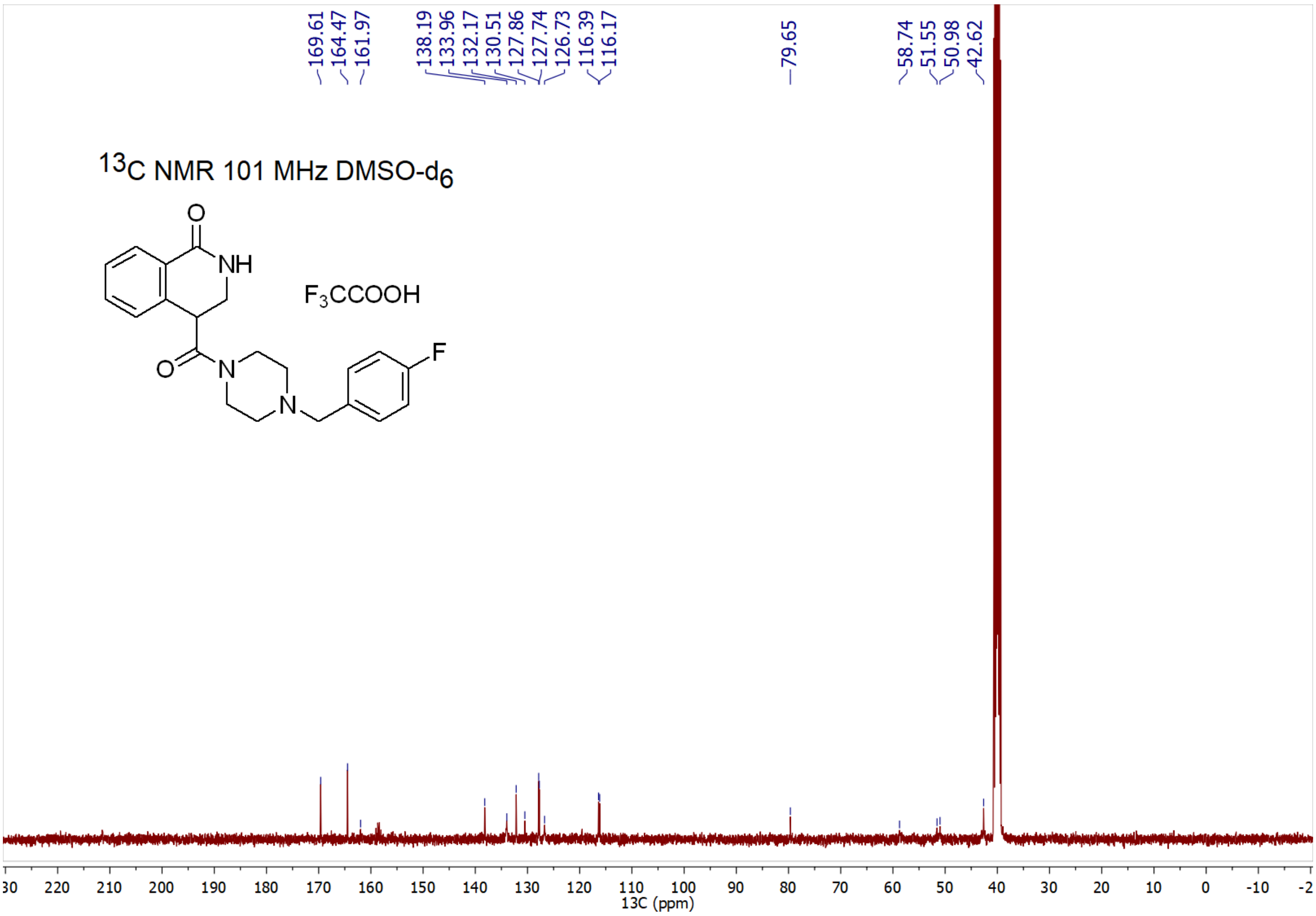

21. ^{1}H and ^{13}C NMR Spectra of **3u**

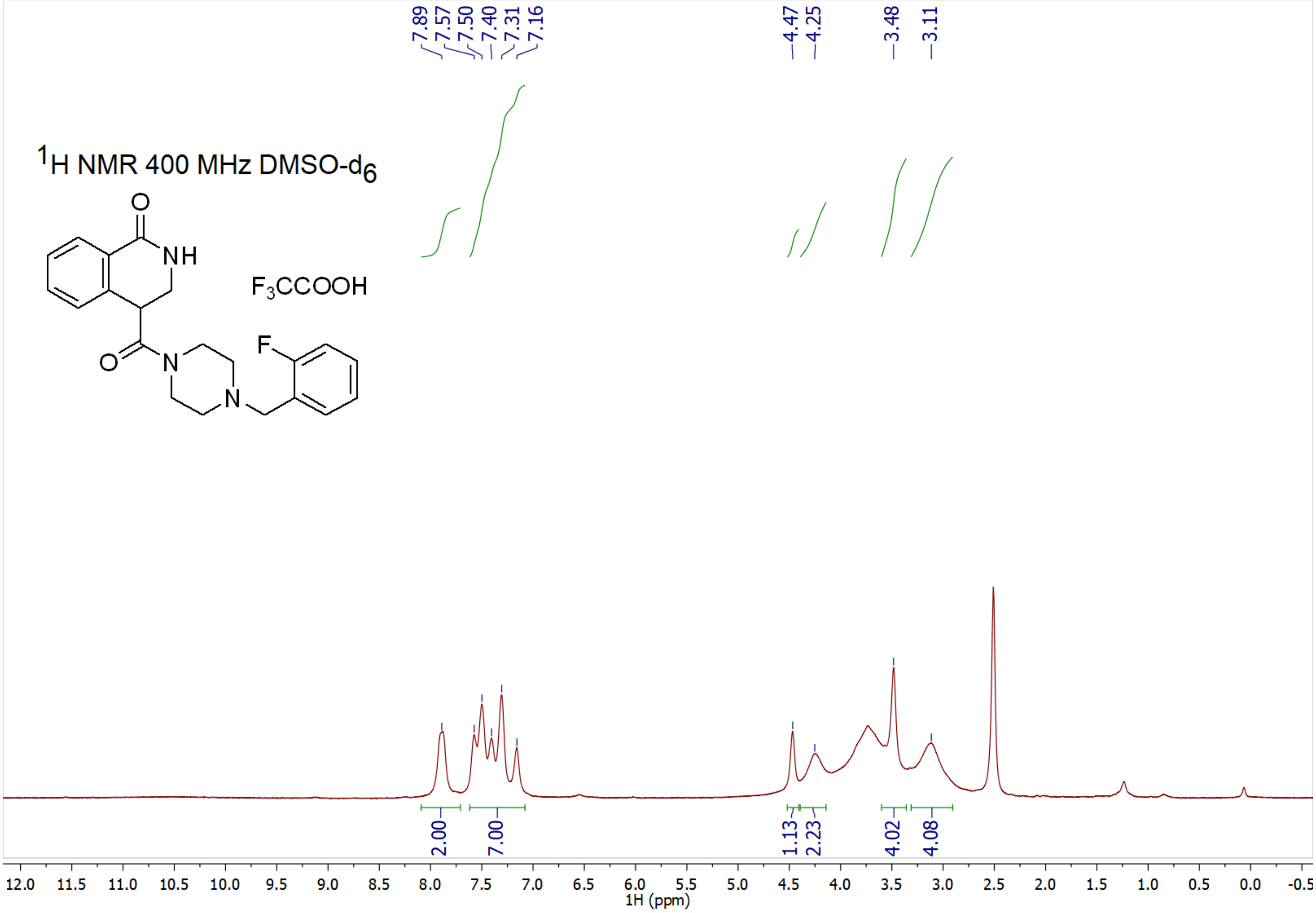

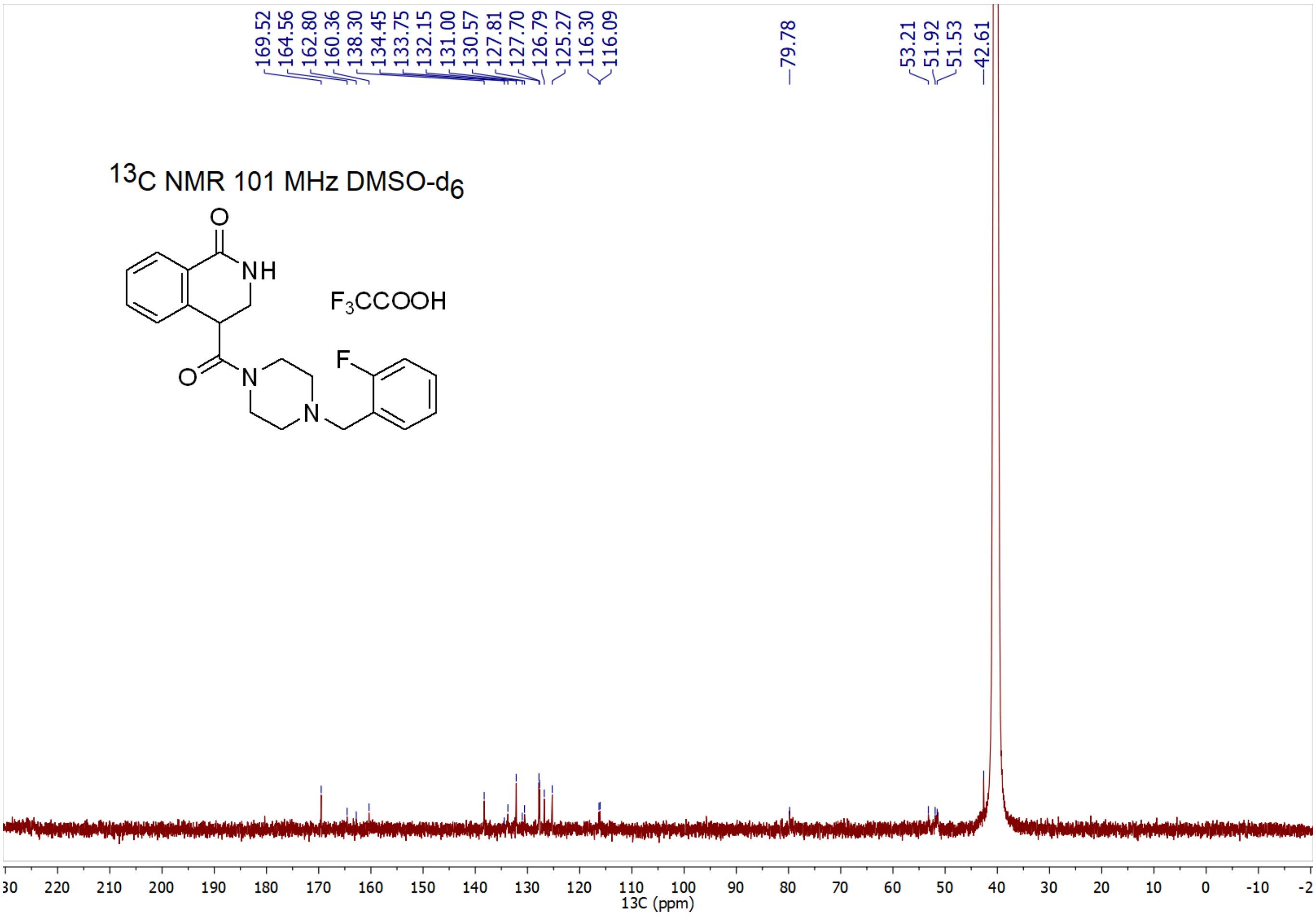

22. ^{1}H and ^{13}C NMR Spectra of **3v**

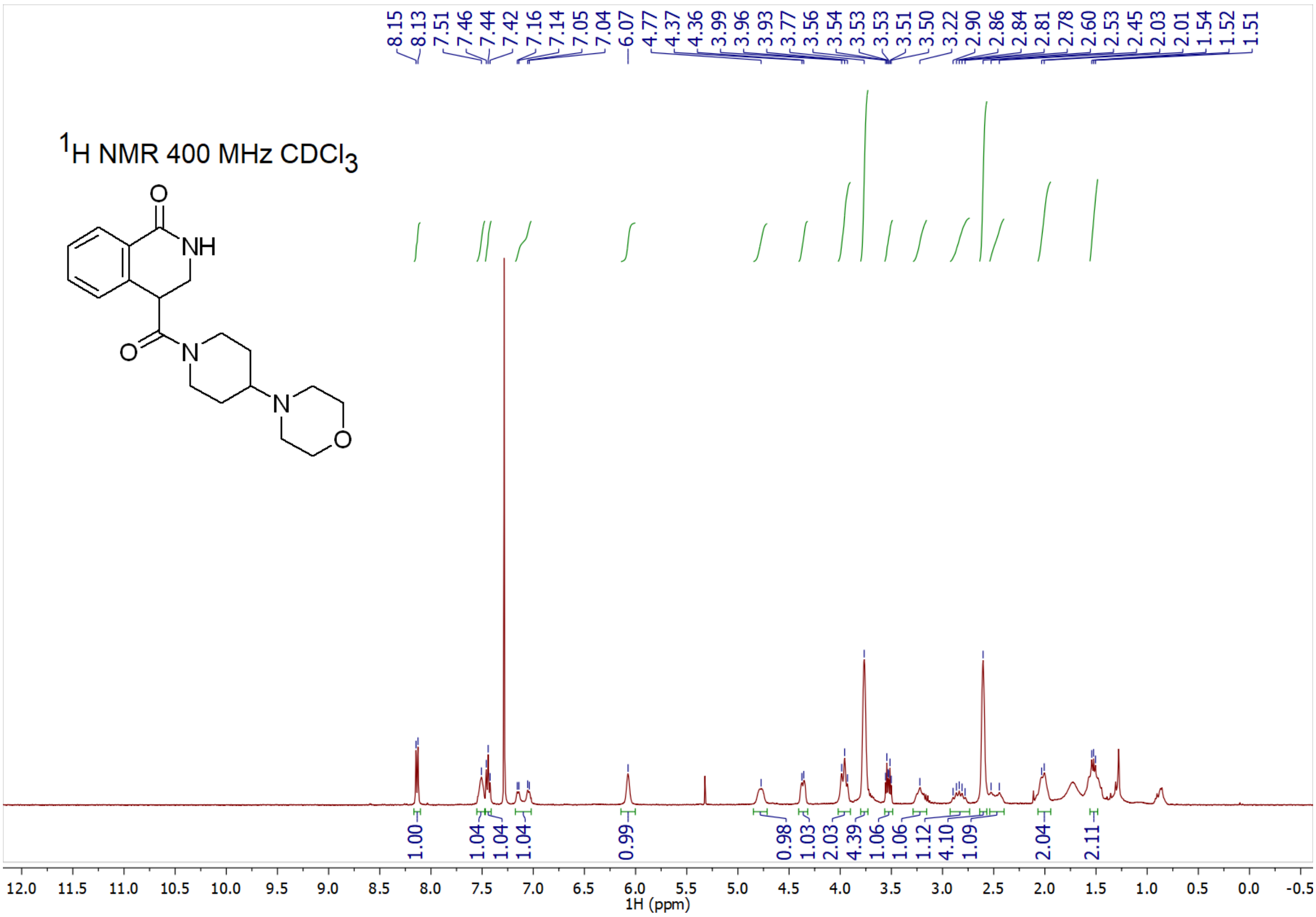

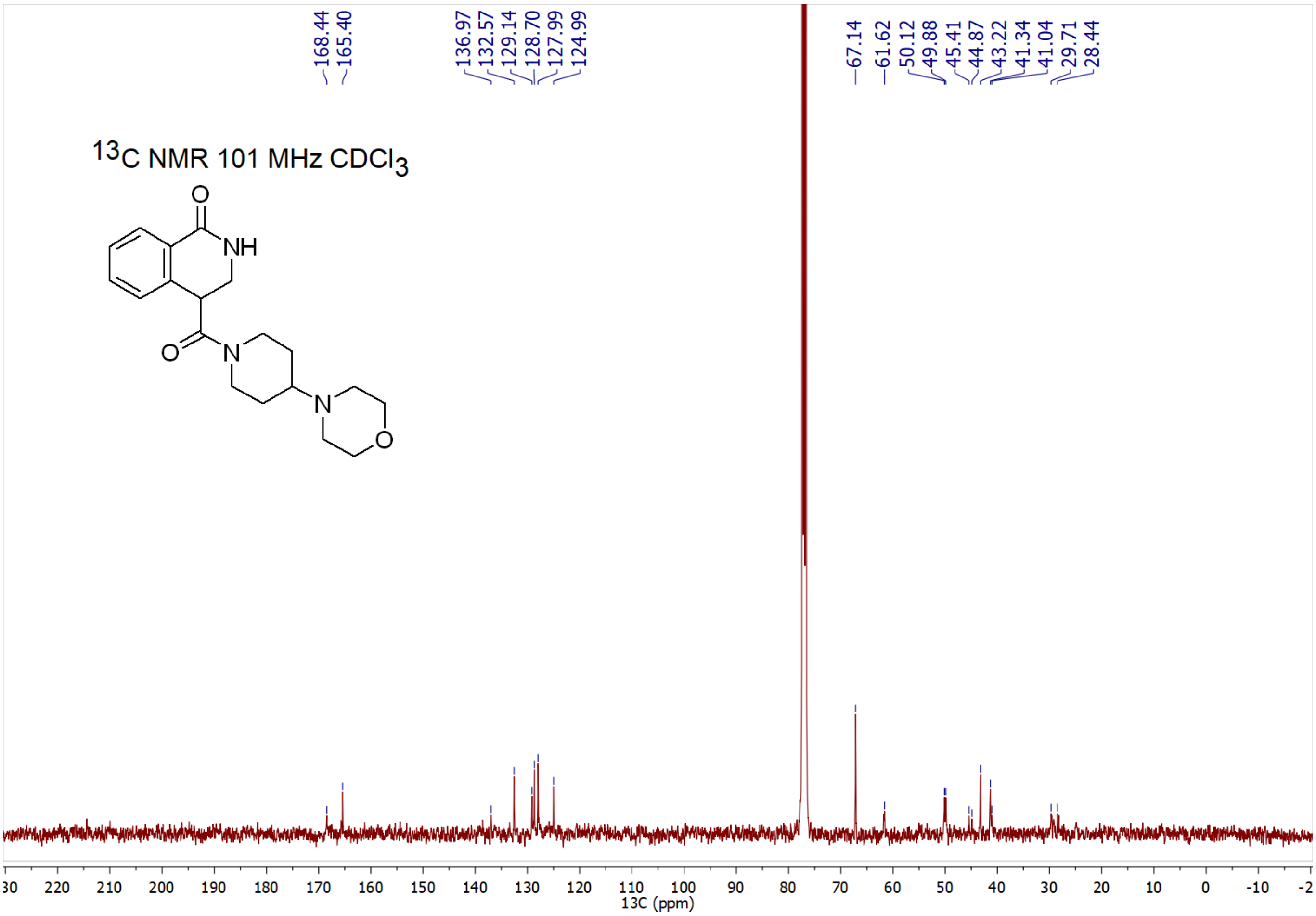

23. ^{1}H and ^{13}C NMR Spectra of **3w**

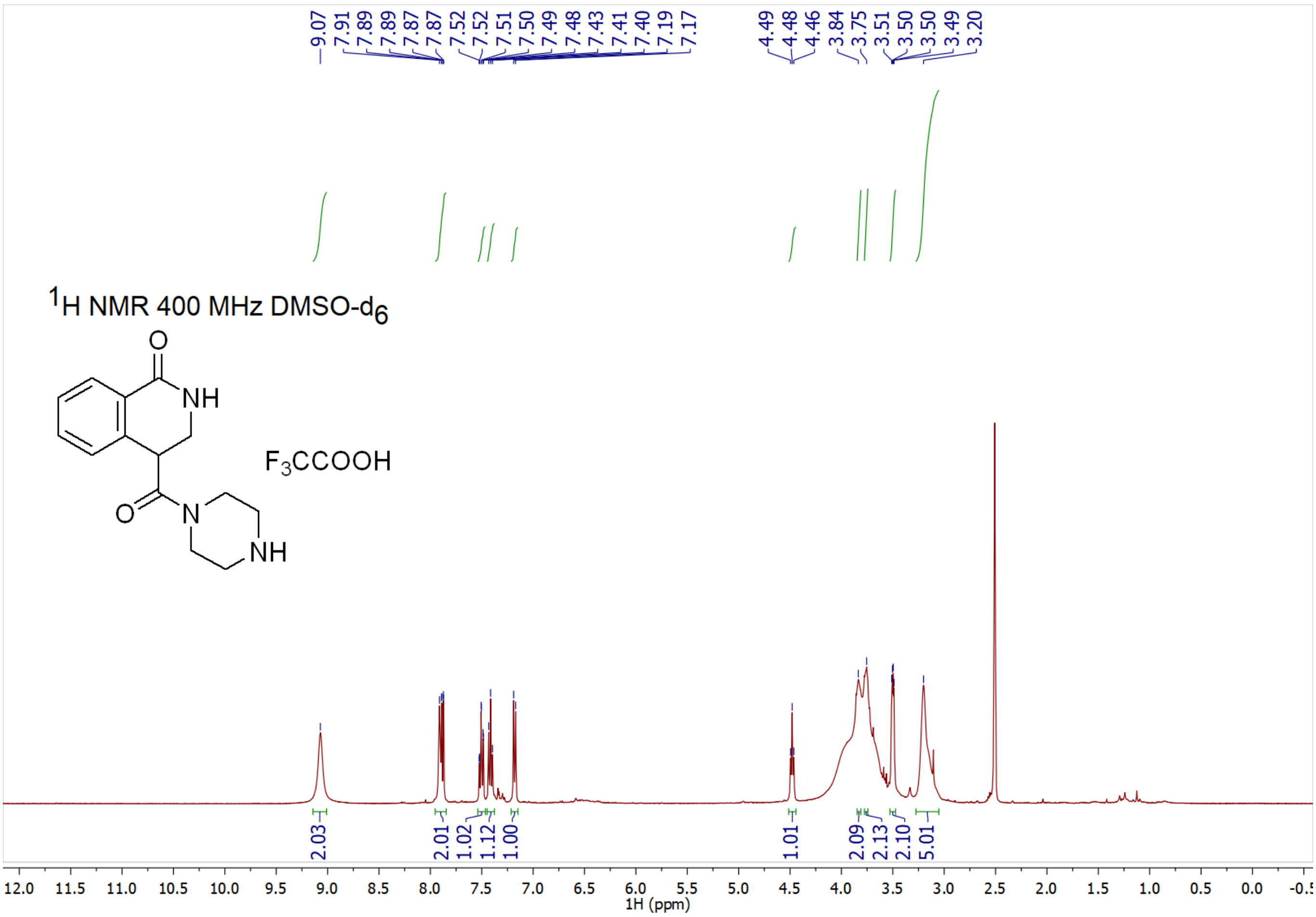

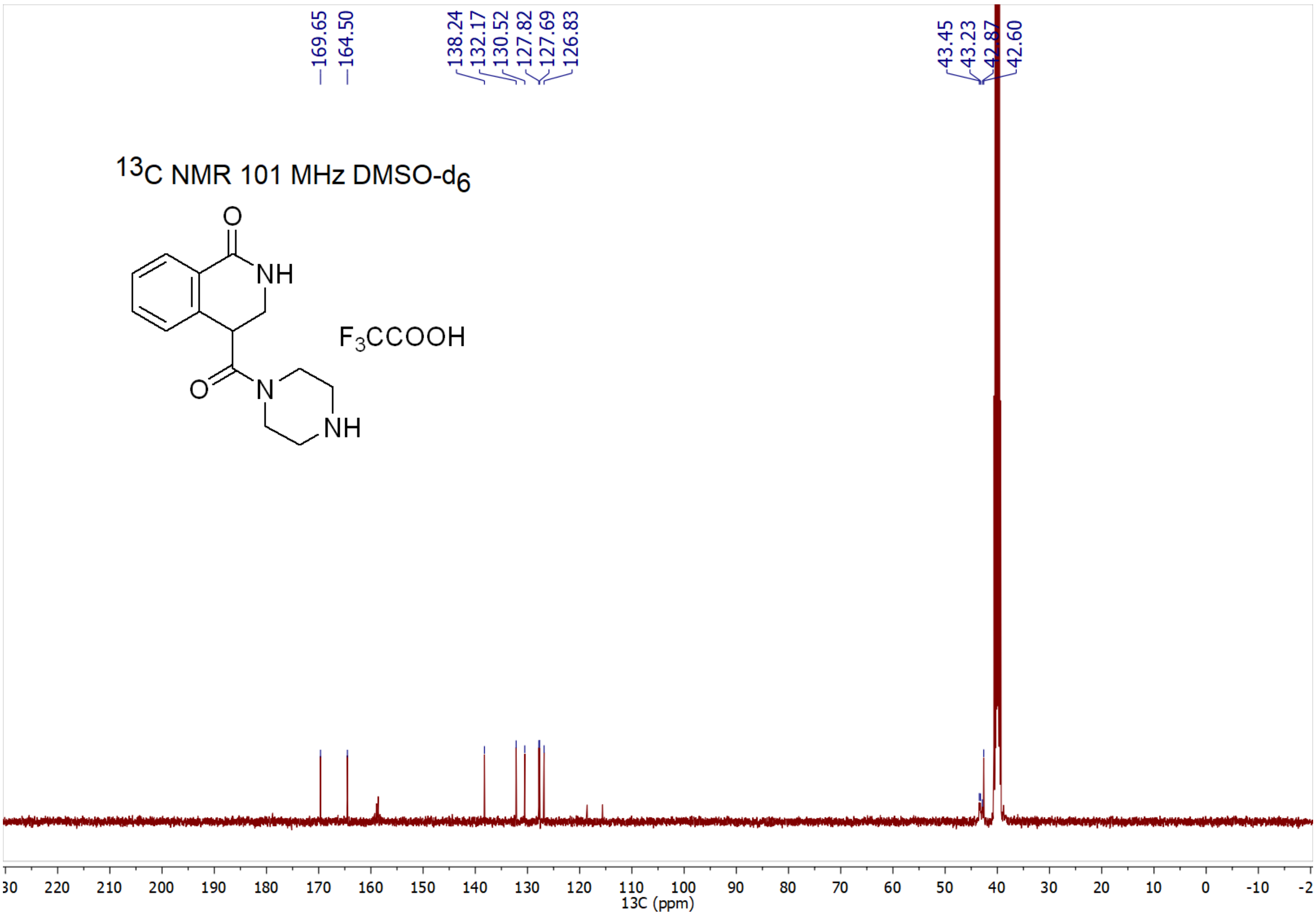

24. ^{1}H and ^{13}C NMR Spectra of **3x**

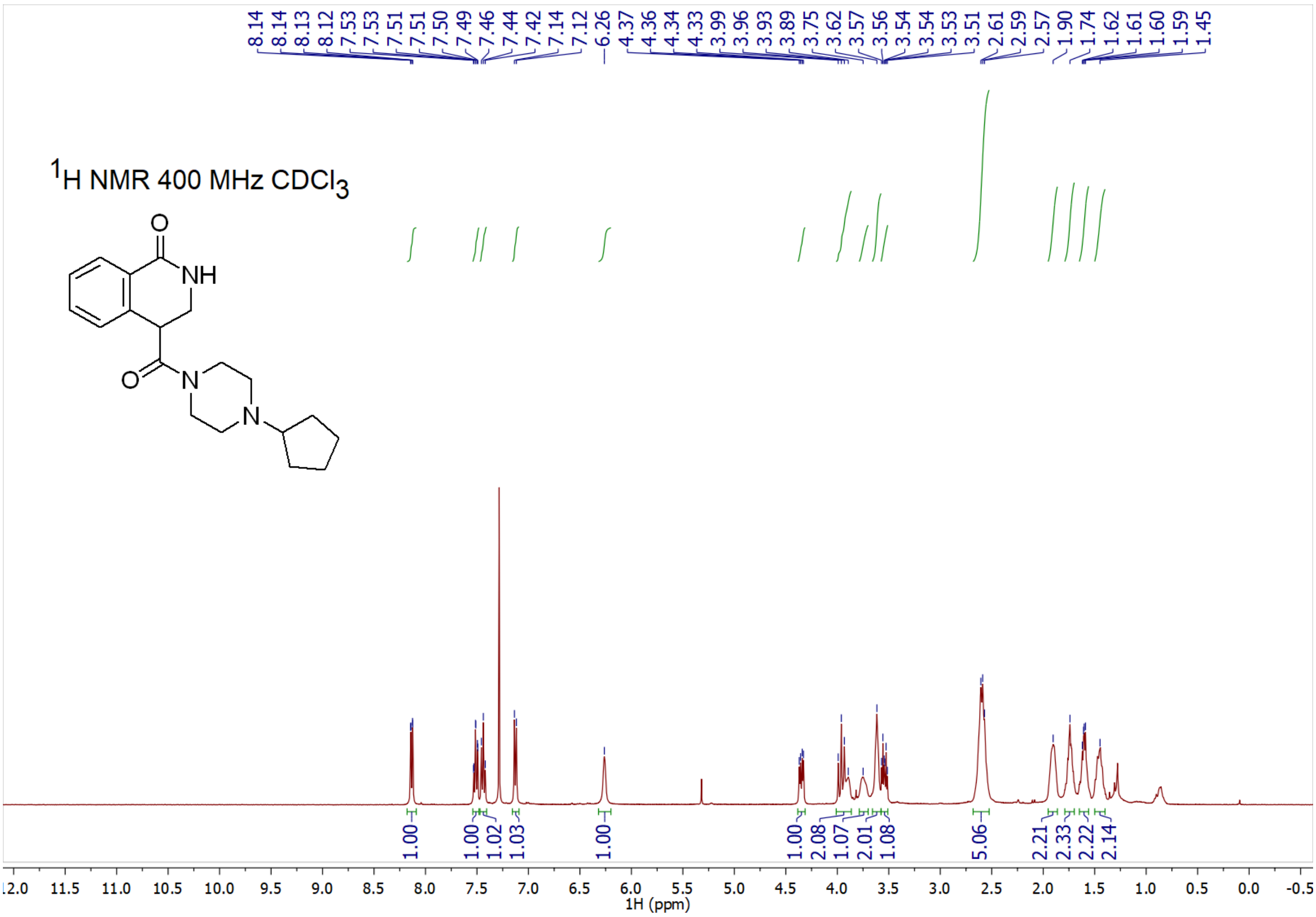

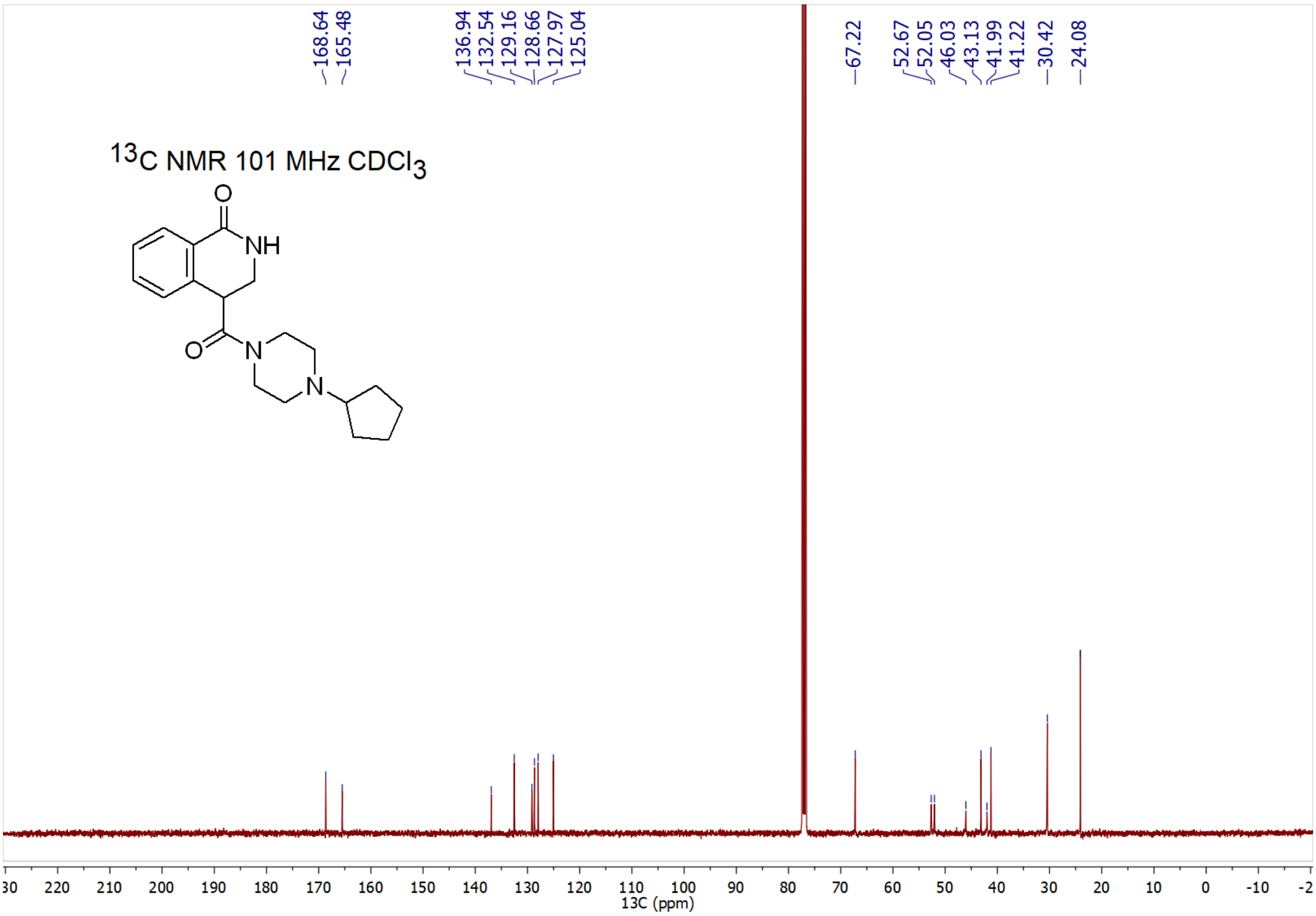

25. ^{1}H and ^{13}C NMR Spectra of **3y**

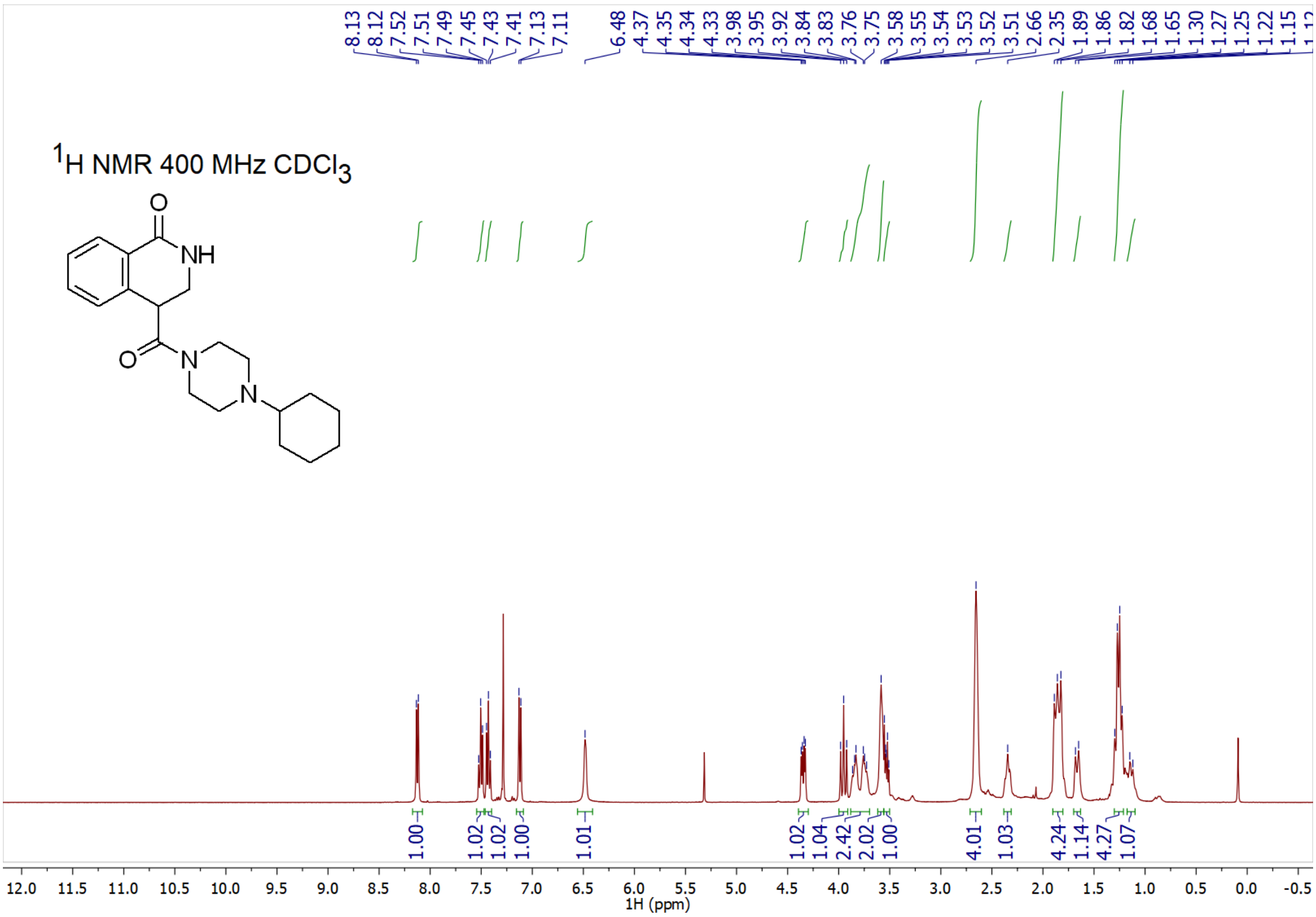

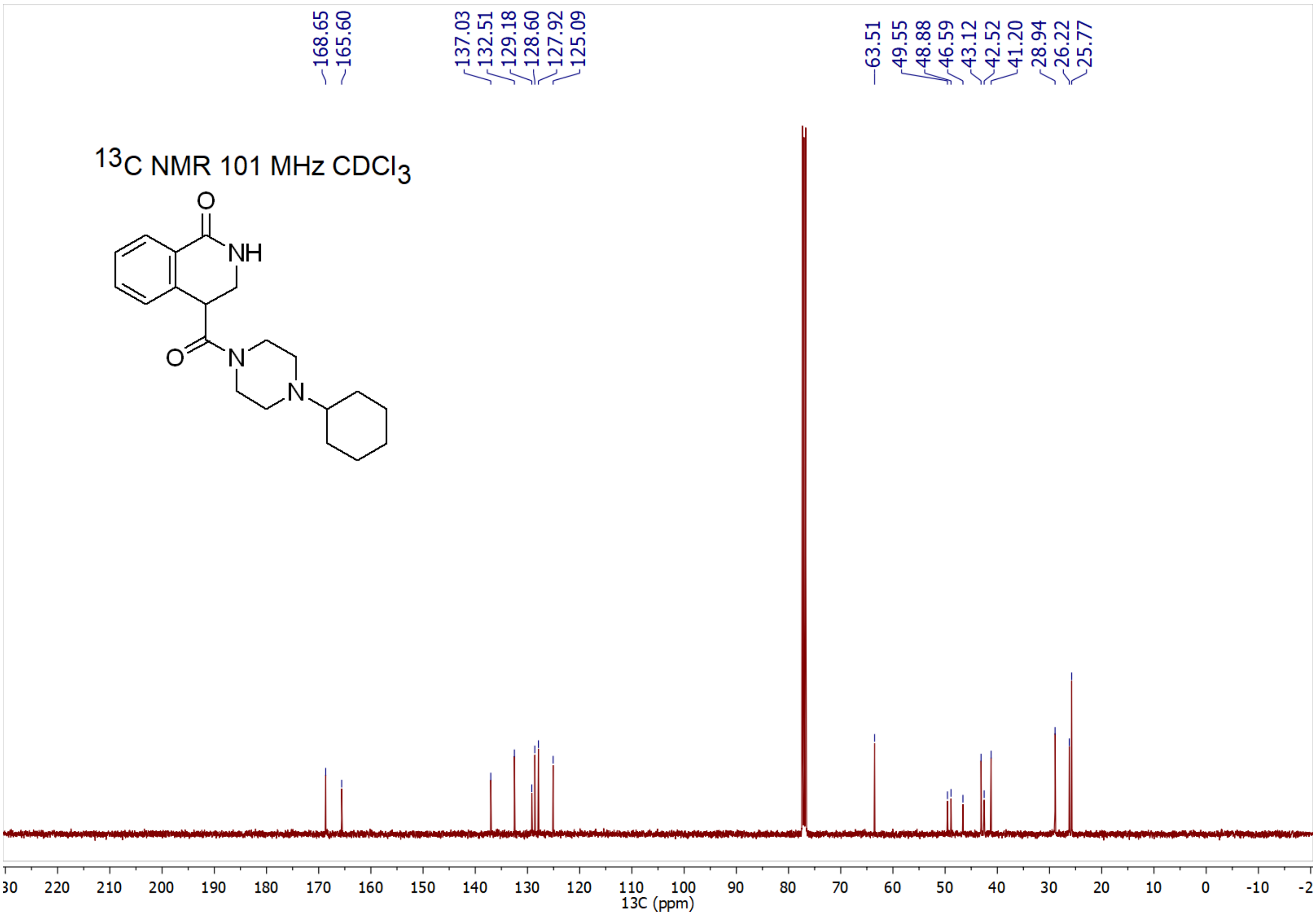

26. ^{1}H and ^{13}C NMR Spectra of **3z**

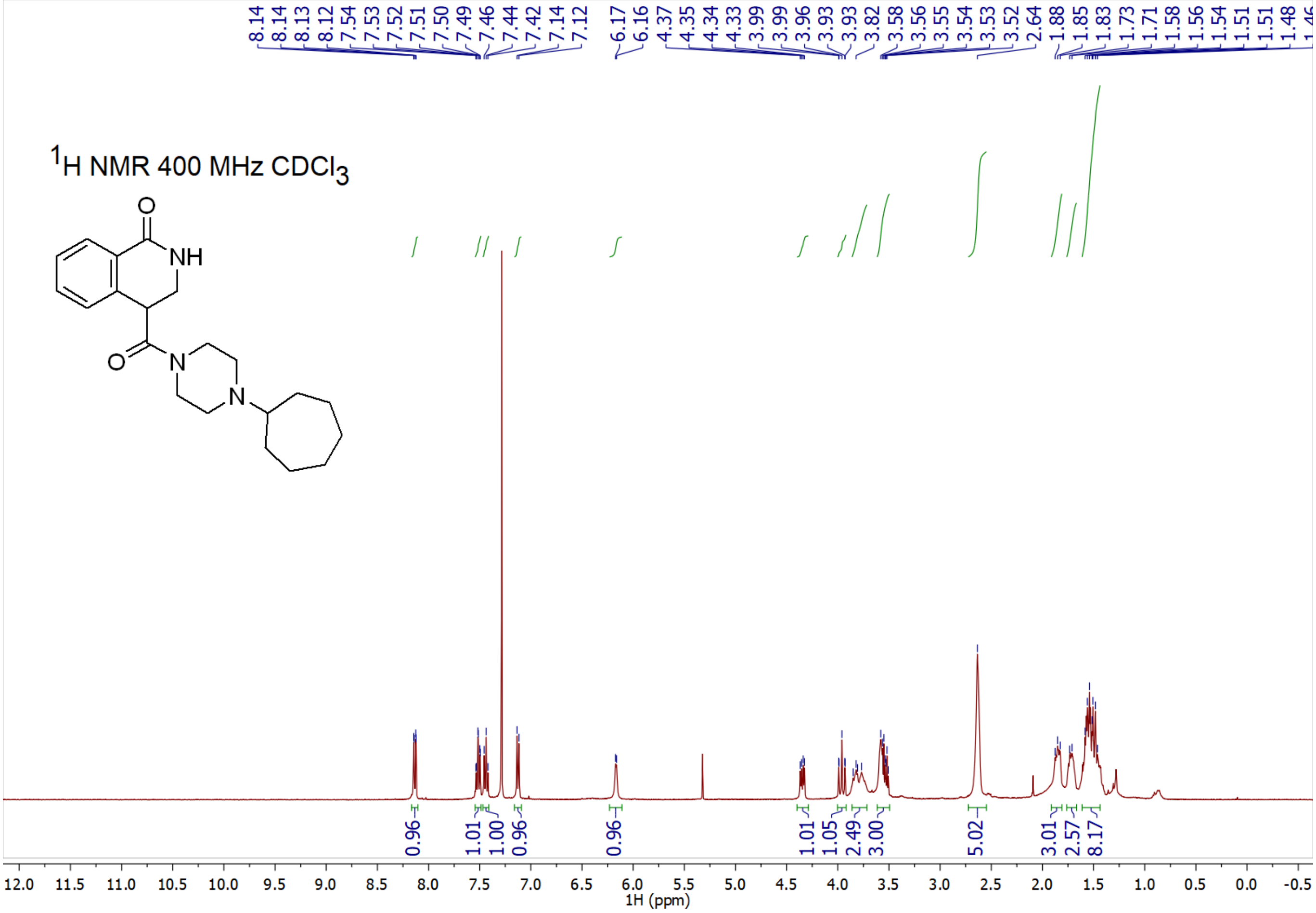

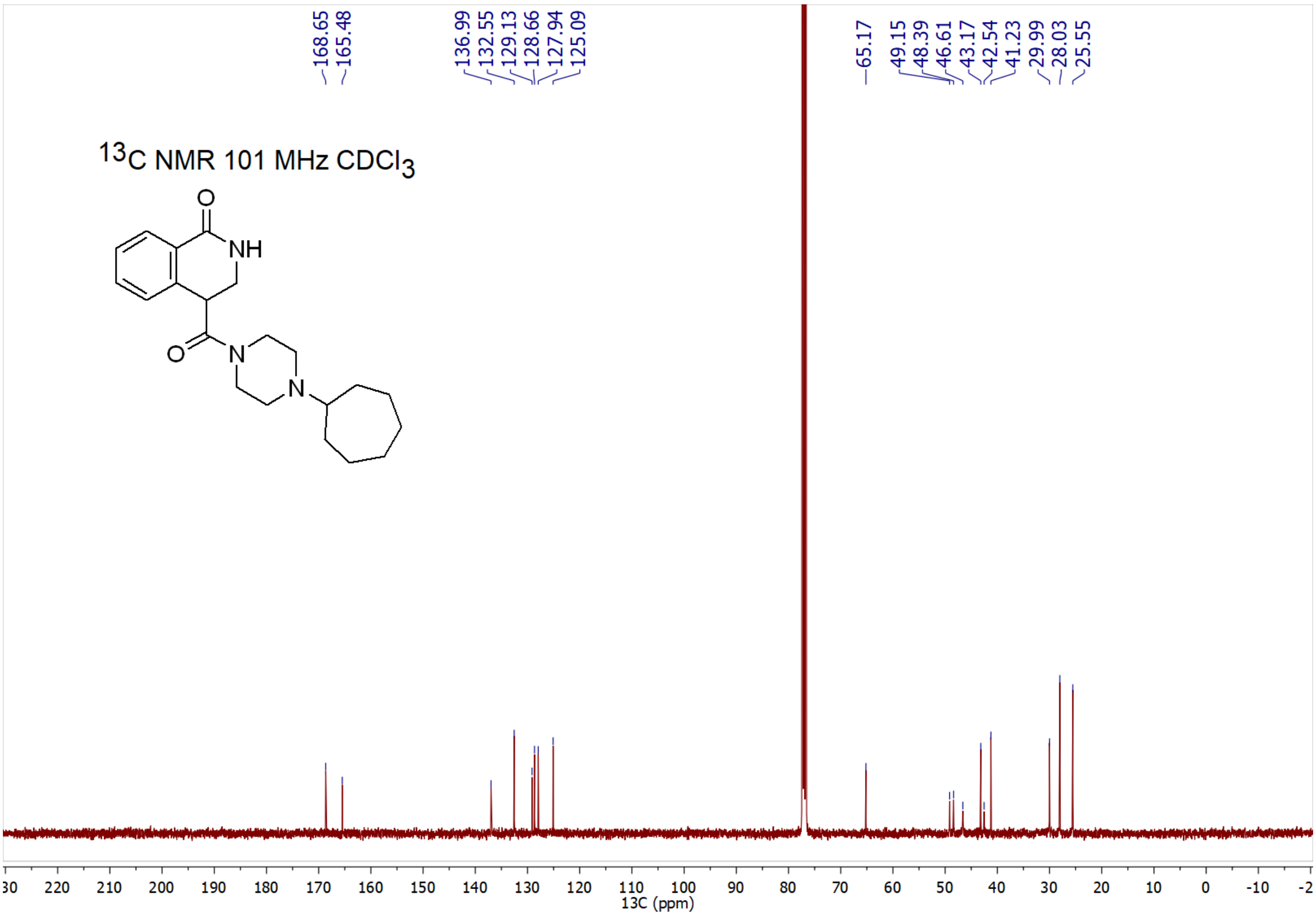

27. ^{1}H and ^{13}C NMR Spectra of **3aa**

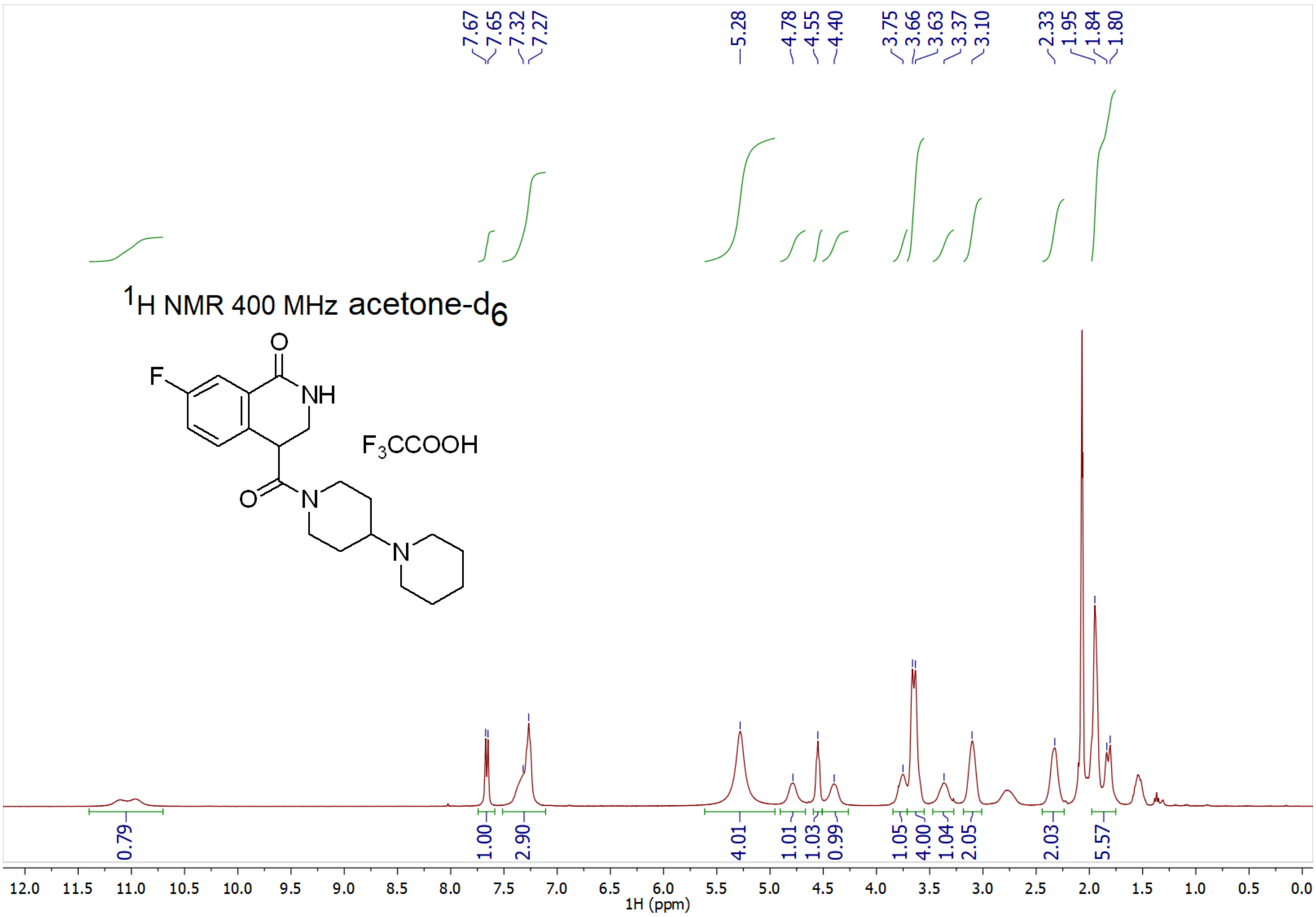

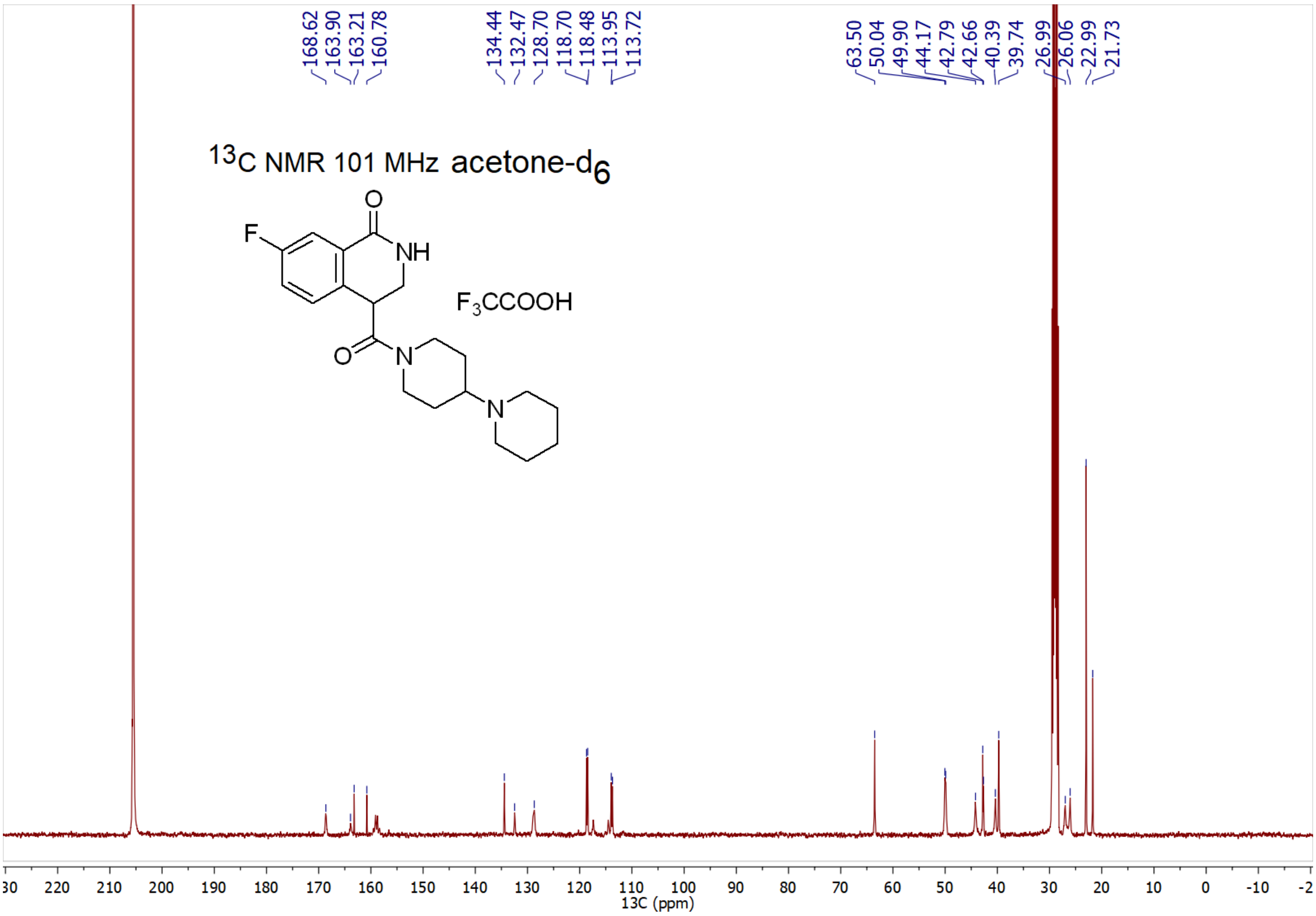

28. ^{1}H and ^{13}C NMR Spectra of **3ab**

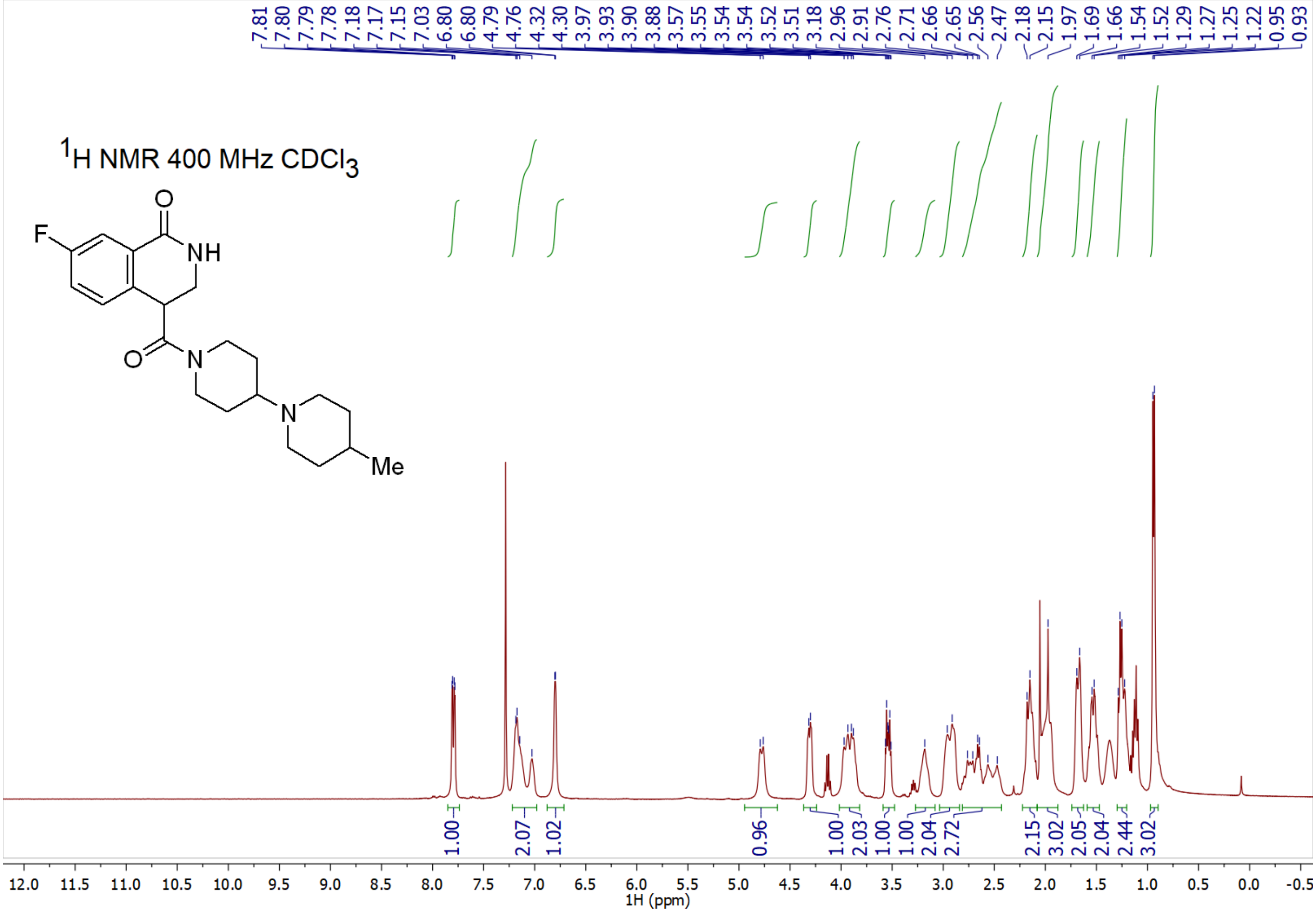

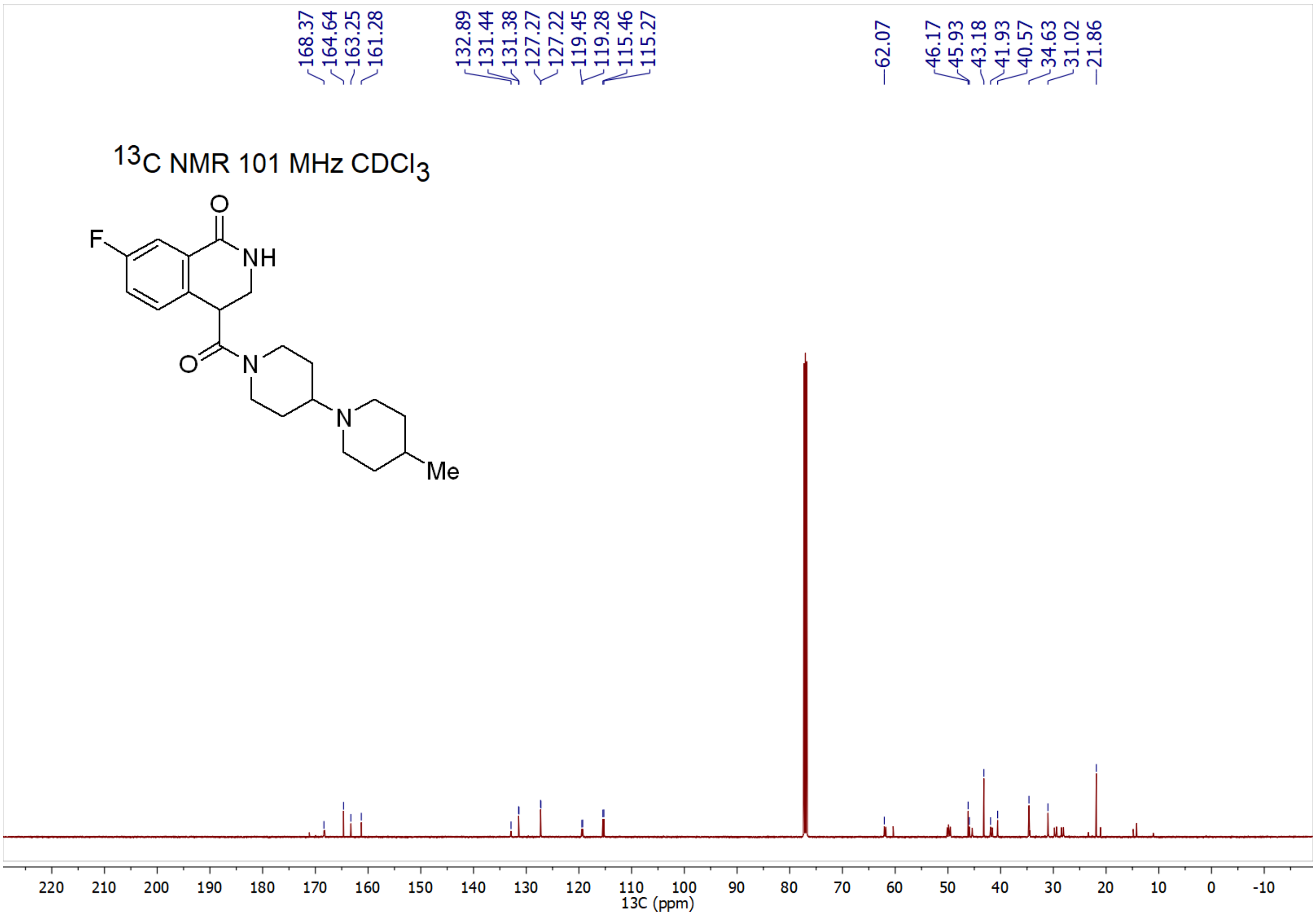

29. ^{1}H and ^{13}C NMR Spectra of **3ac**

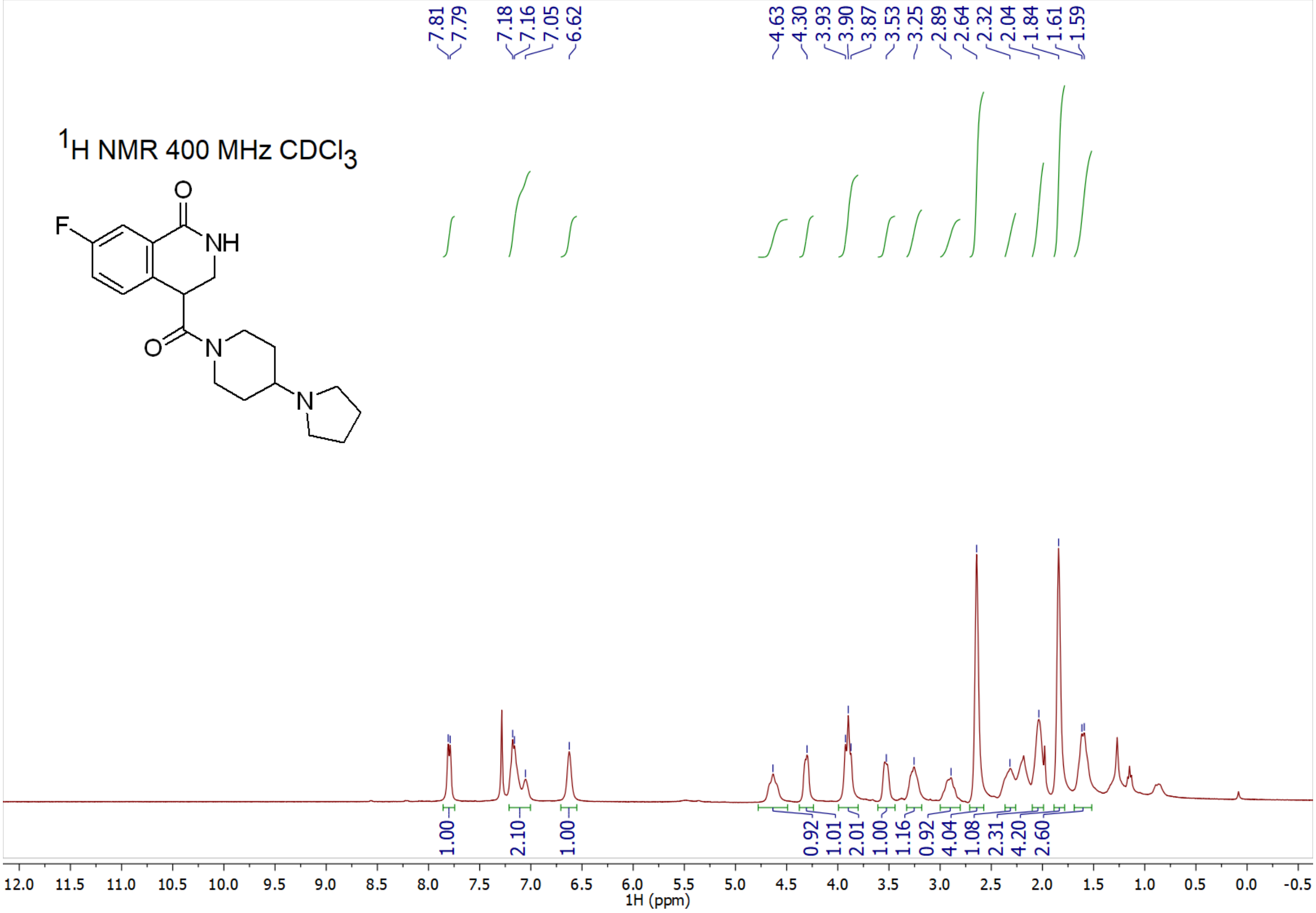

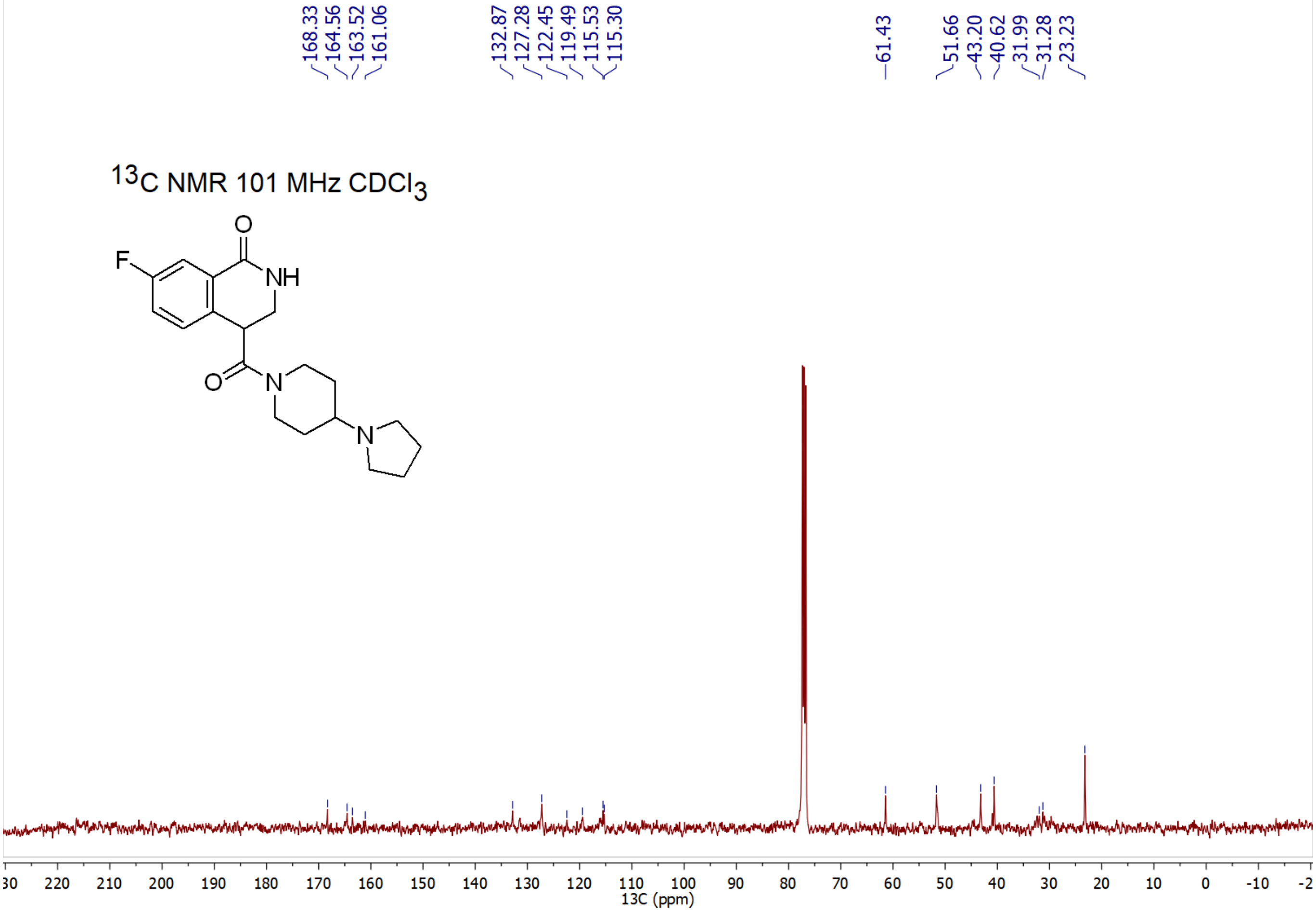

30. ^{1}H and ^{13}C NMR Spectra of **3ad**

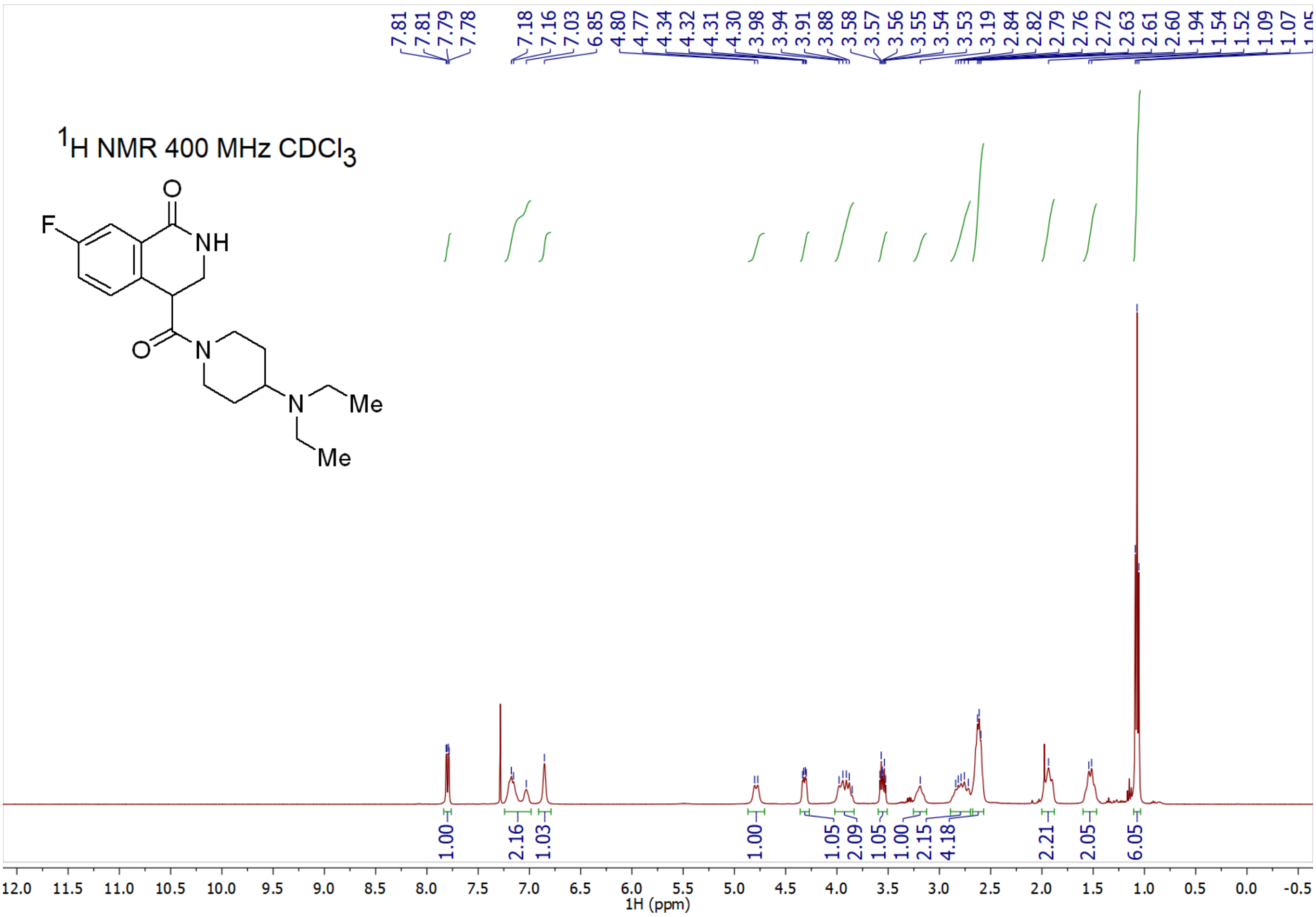

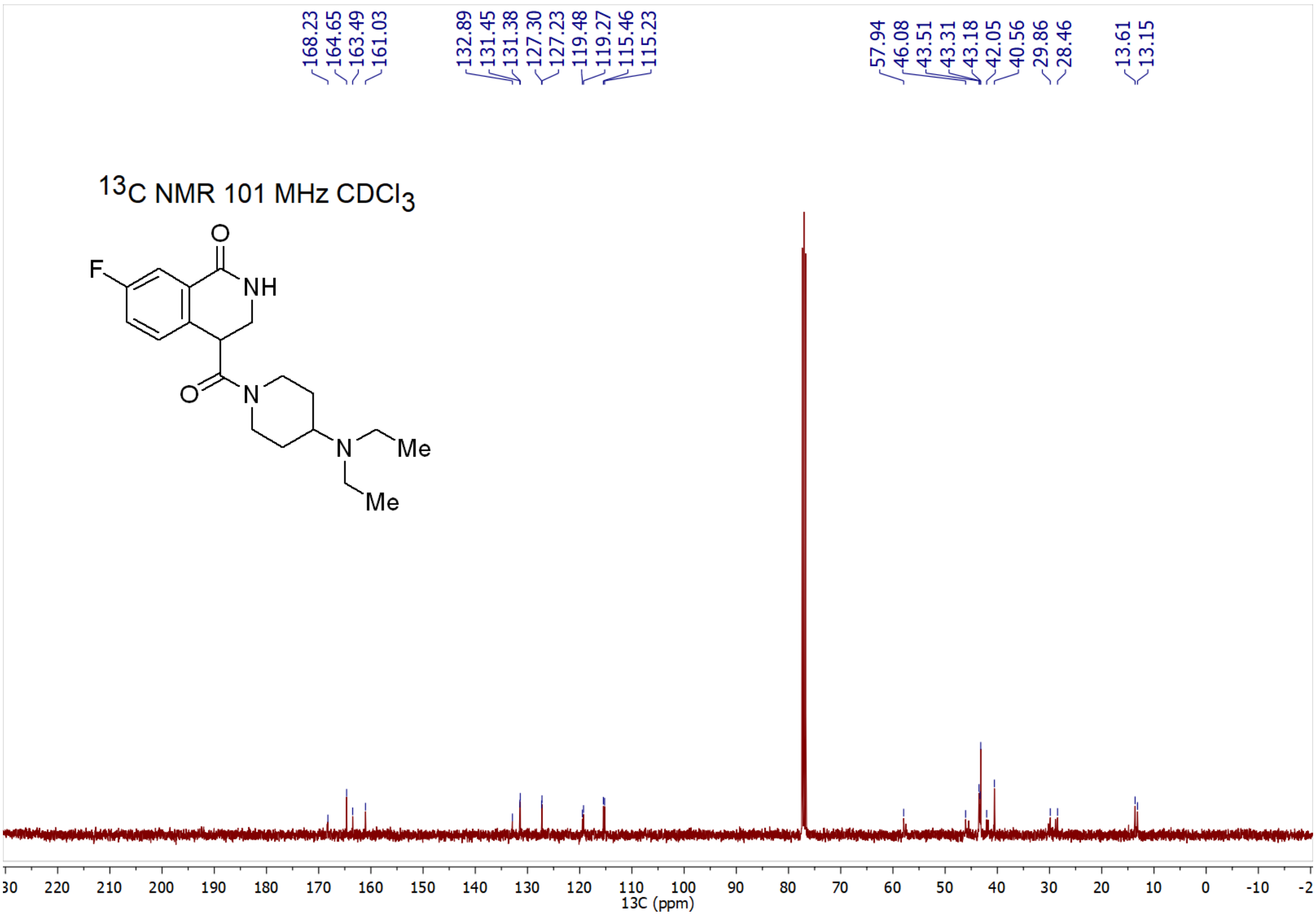

31. ^{1}H and ^{13}C NMR Spectra of **3ae**

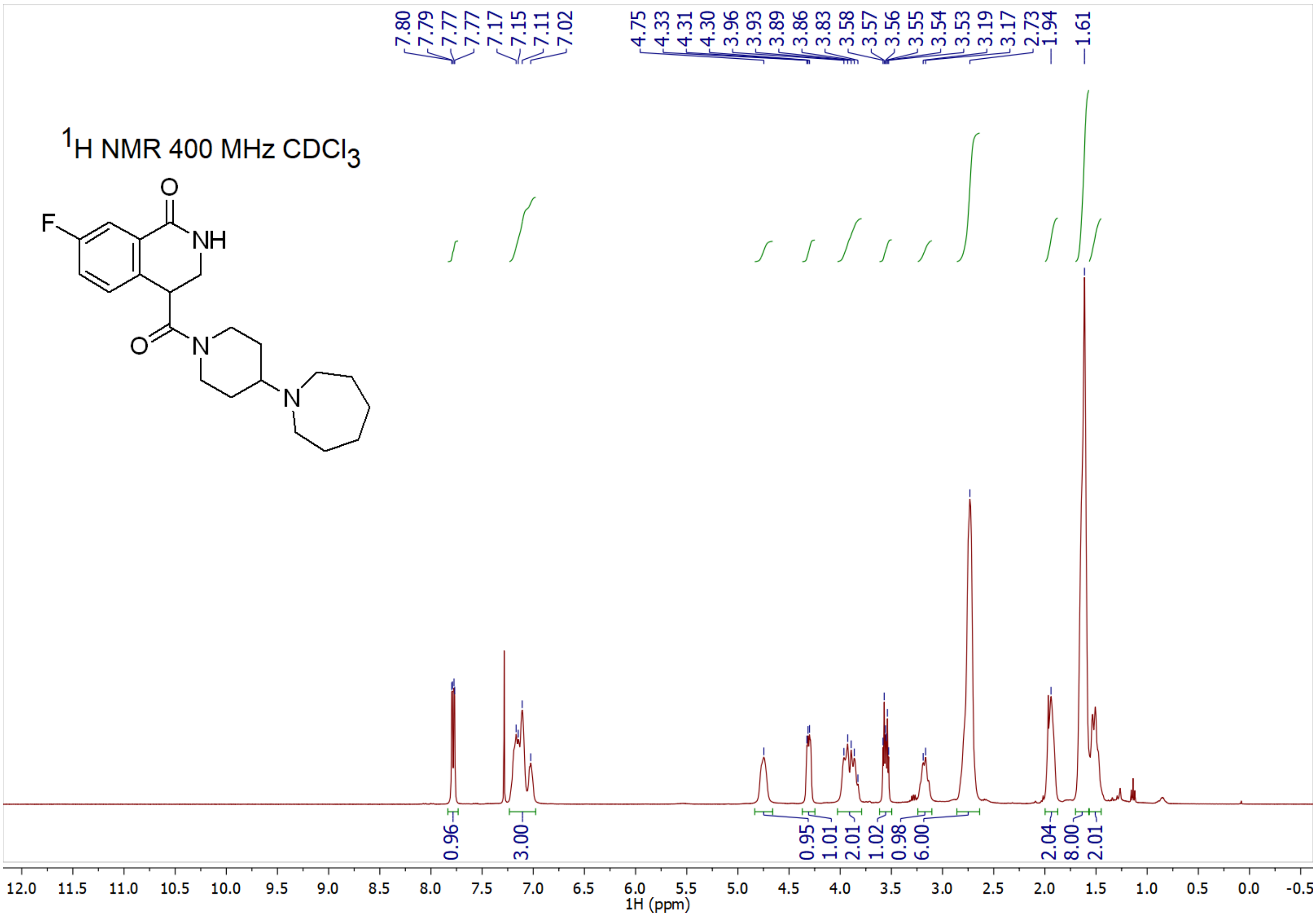

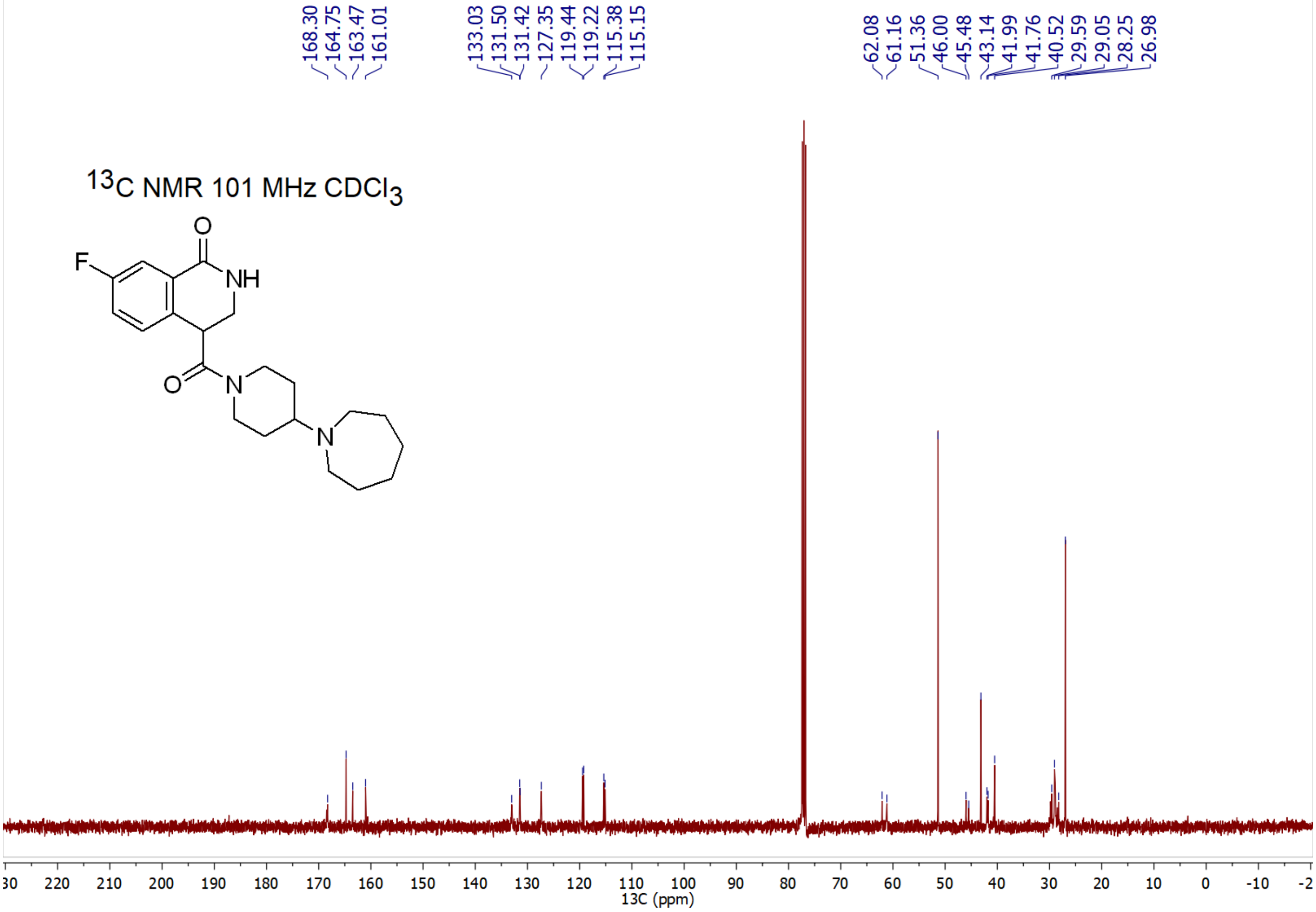

32. ^{1}H and ^{13}C NMR Spectra of **3af**

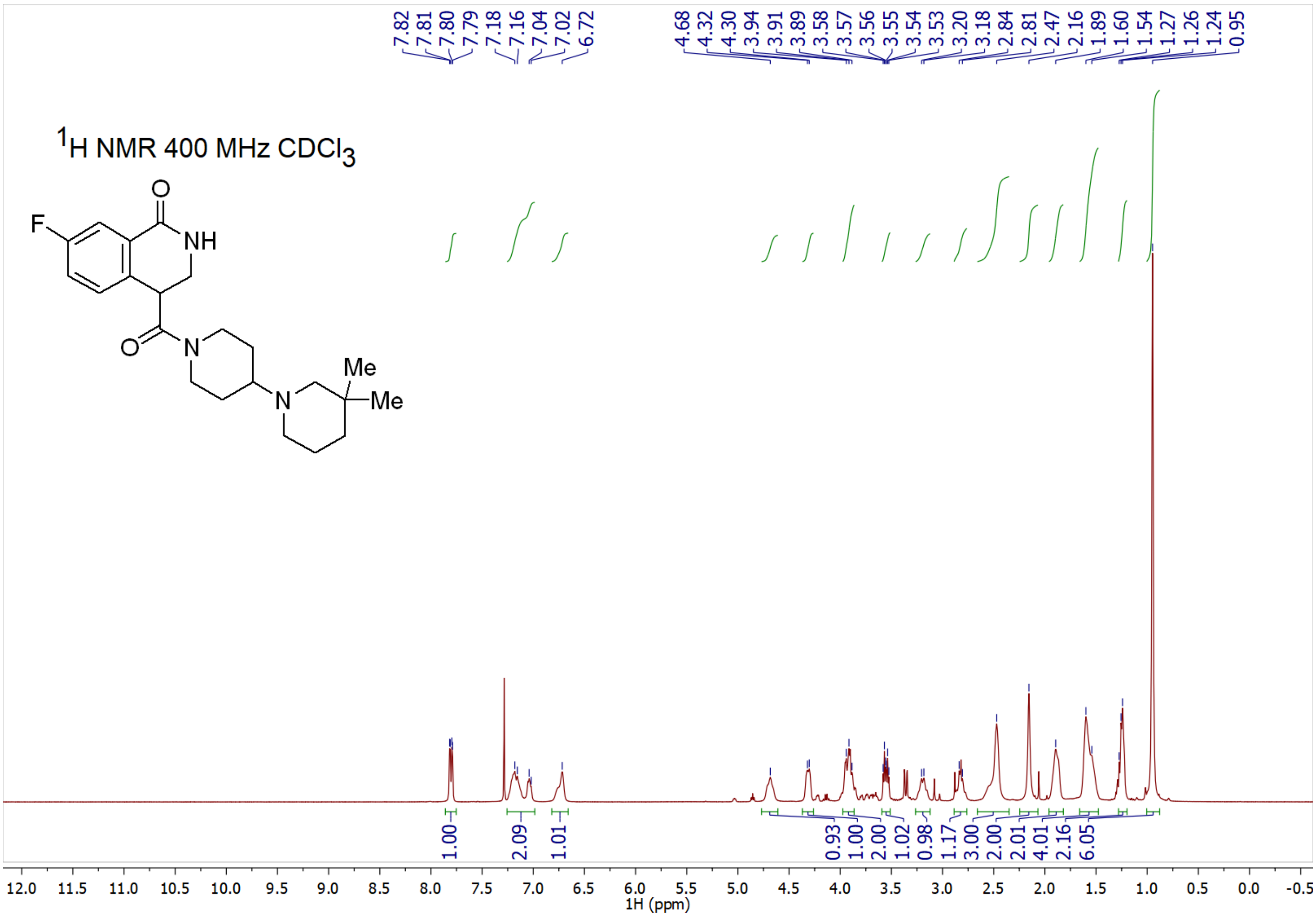

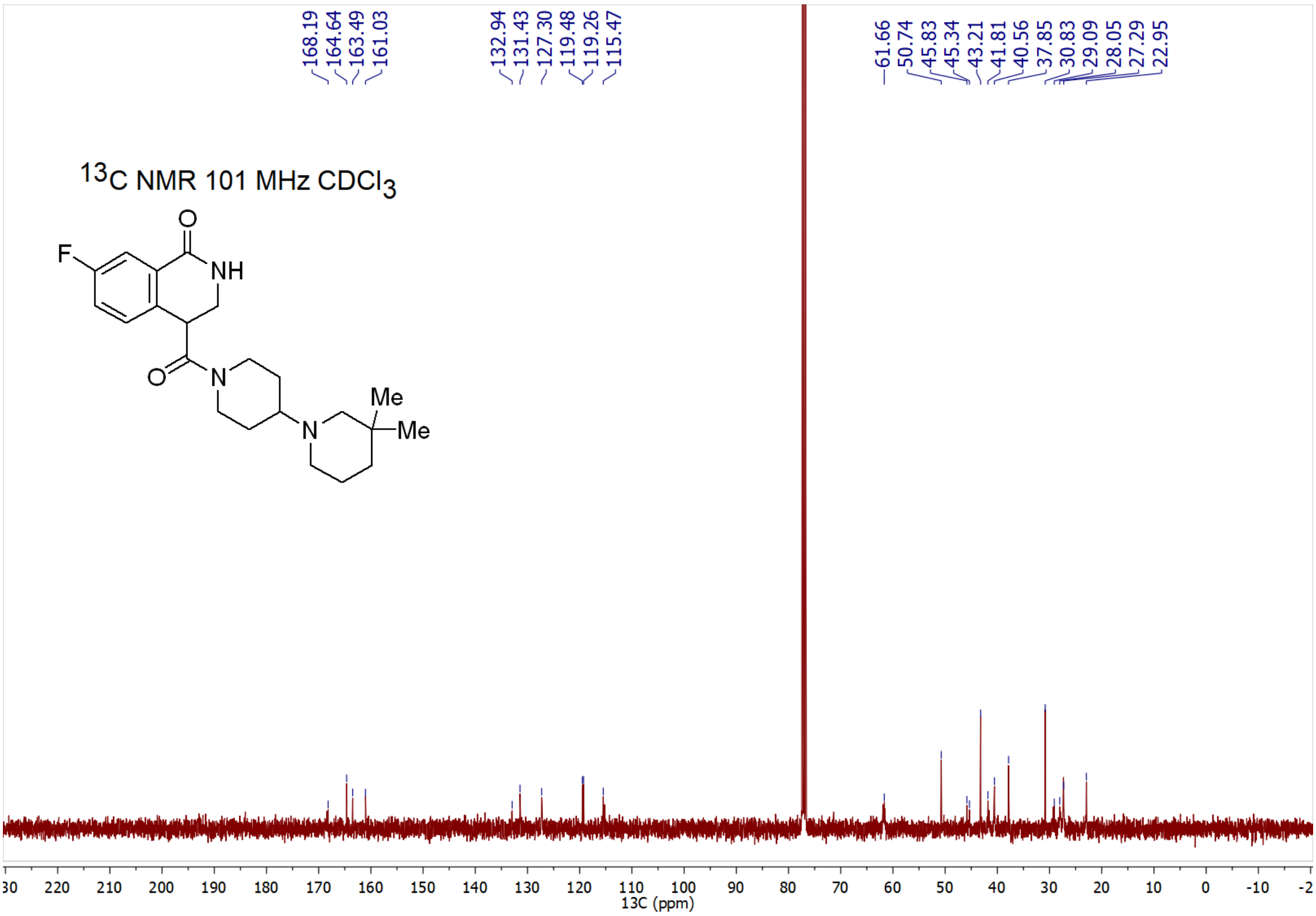

33. ^{1}H and ^{13}C NMR Spectra of **3ag**

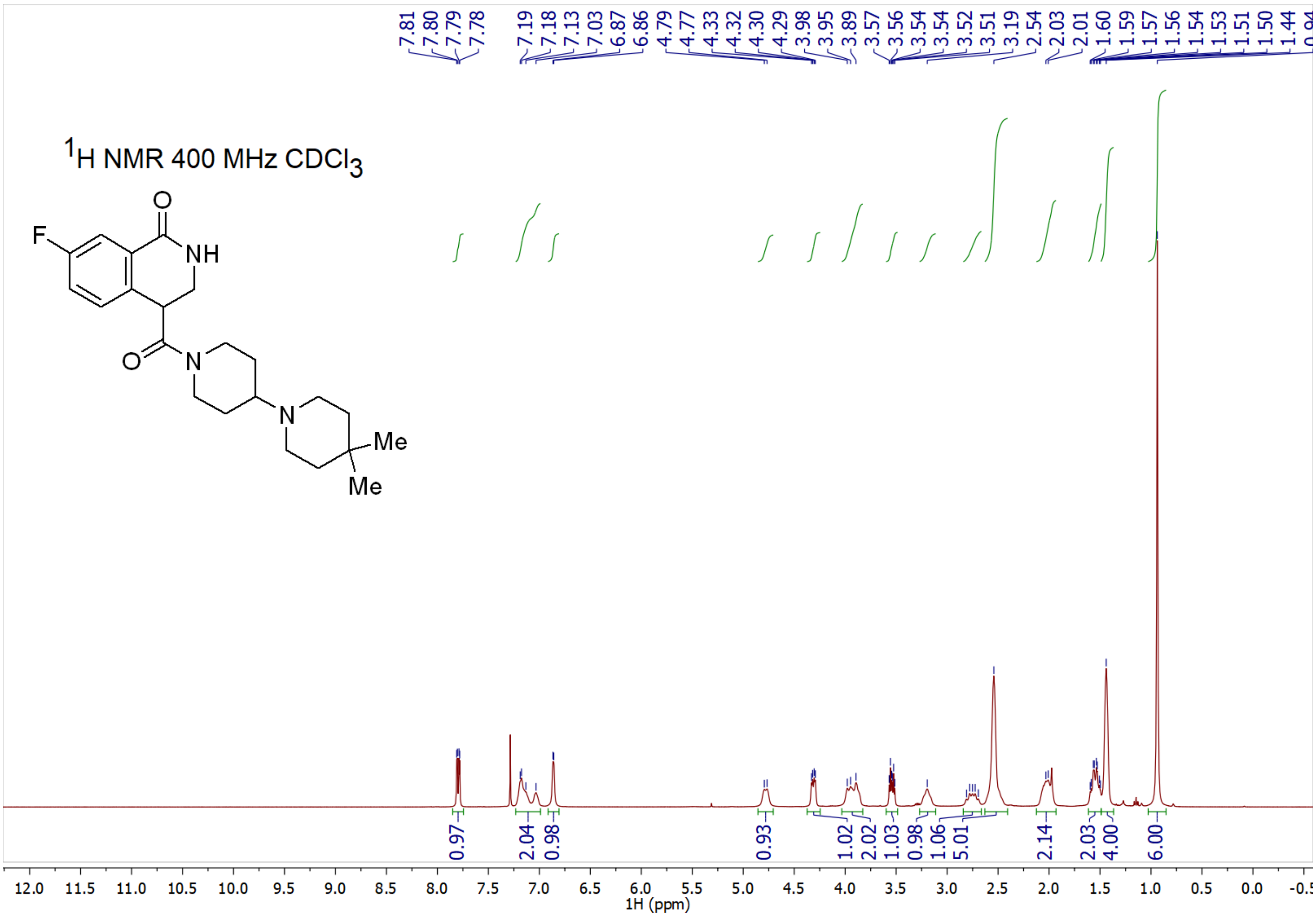

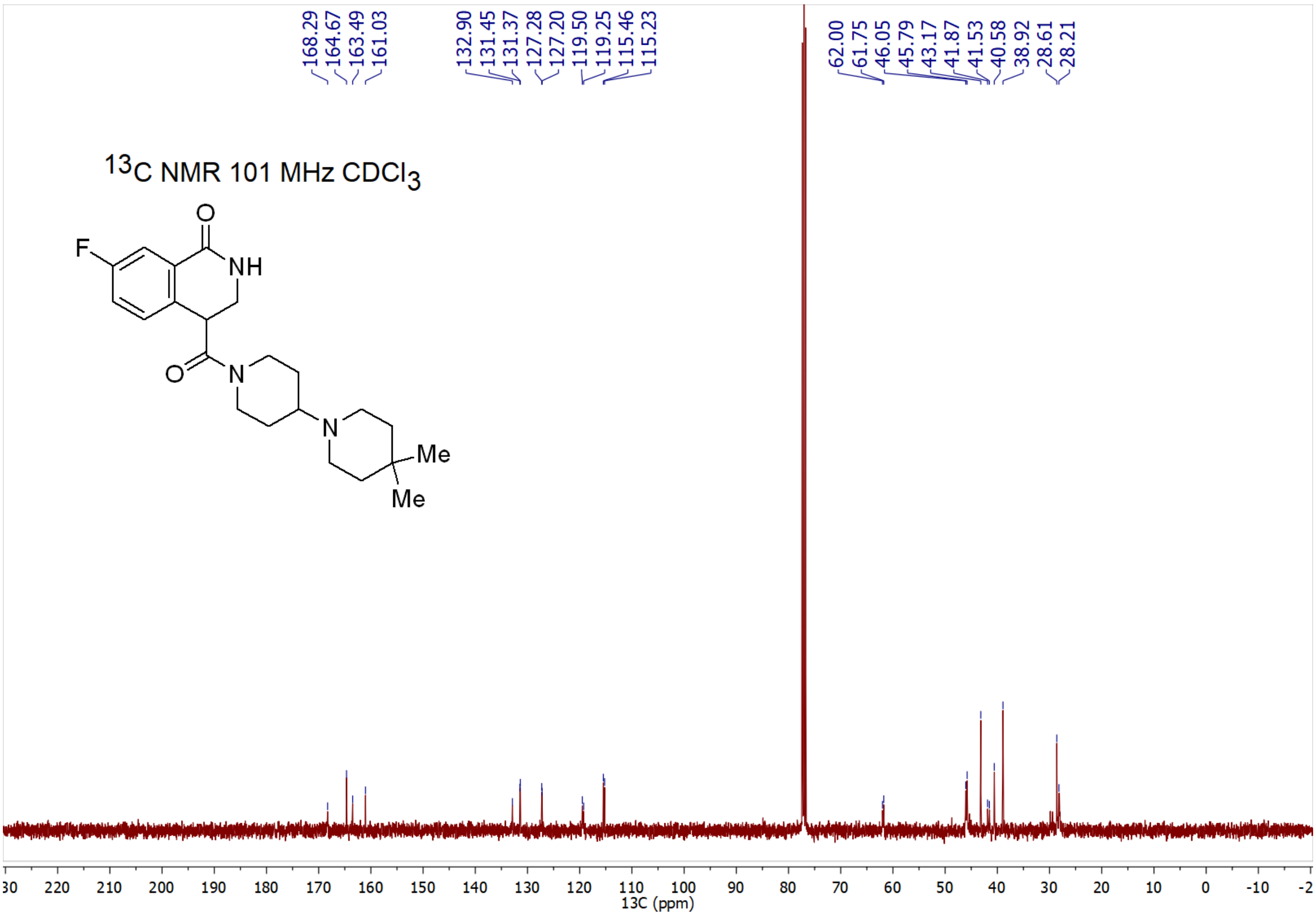

34. ^{1}H and ^{13}C NMR Spectra of **3ah**

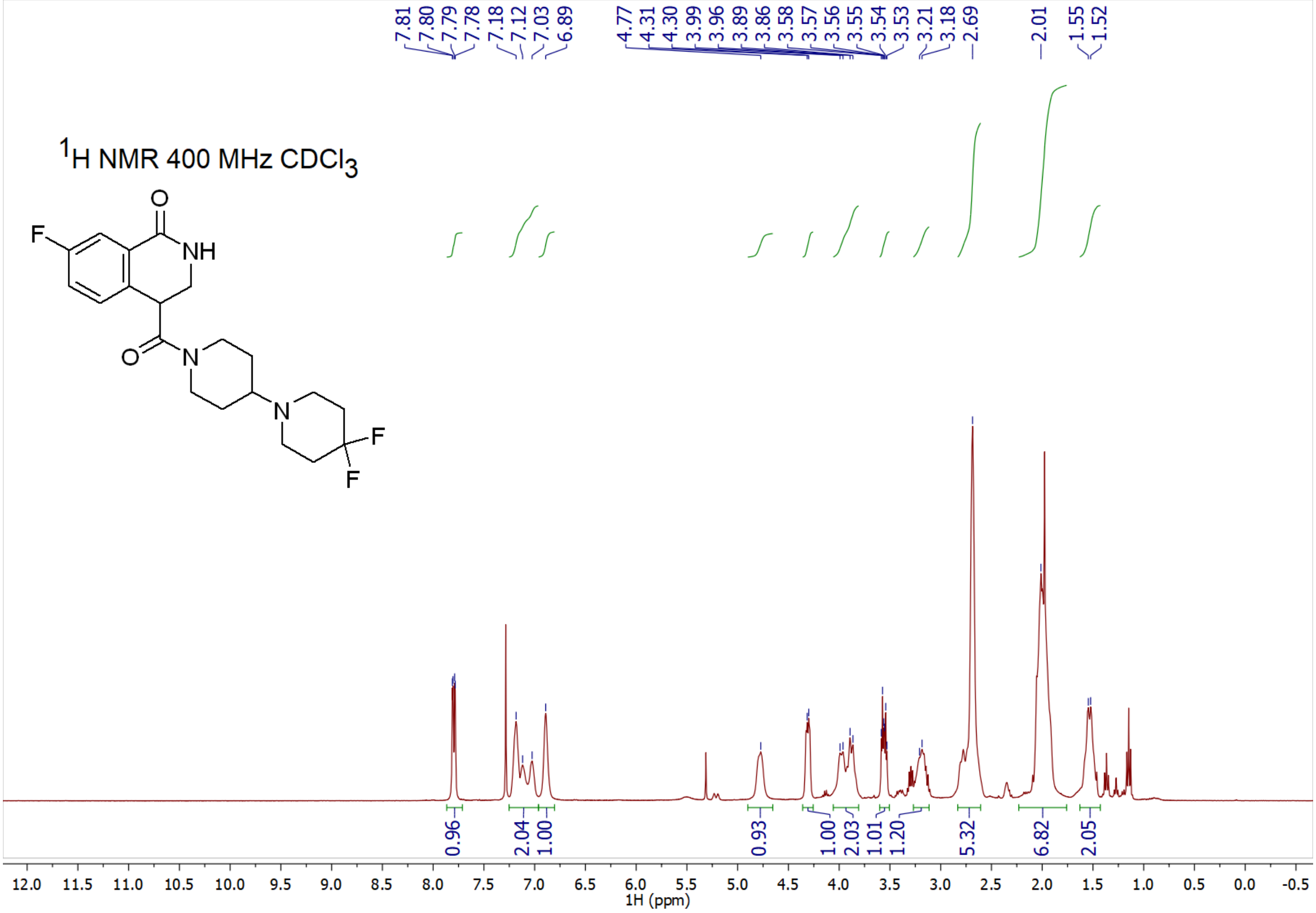

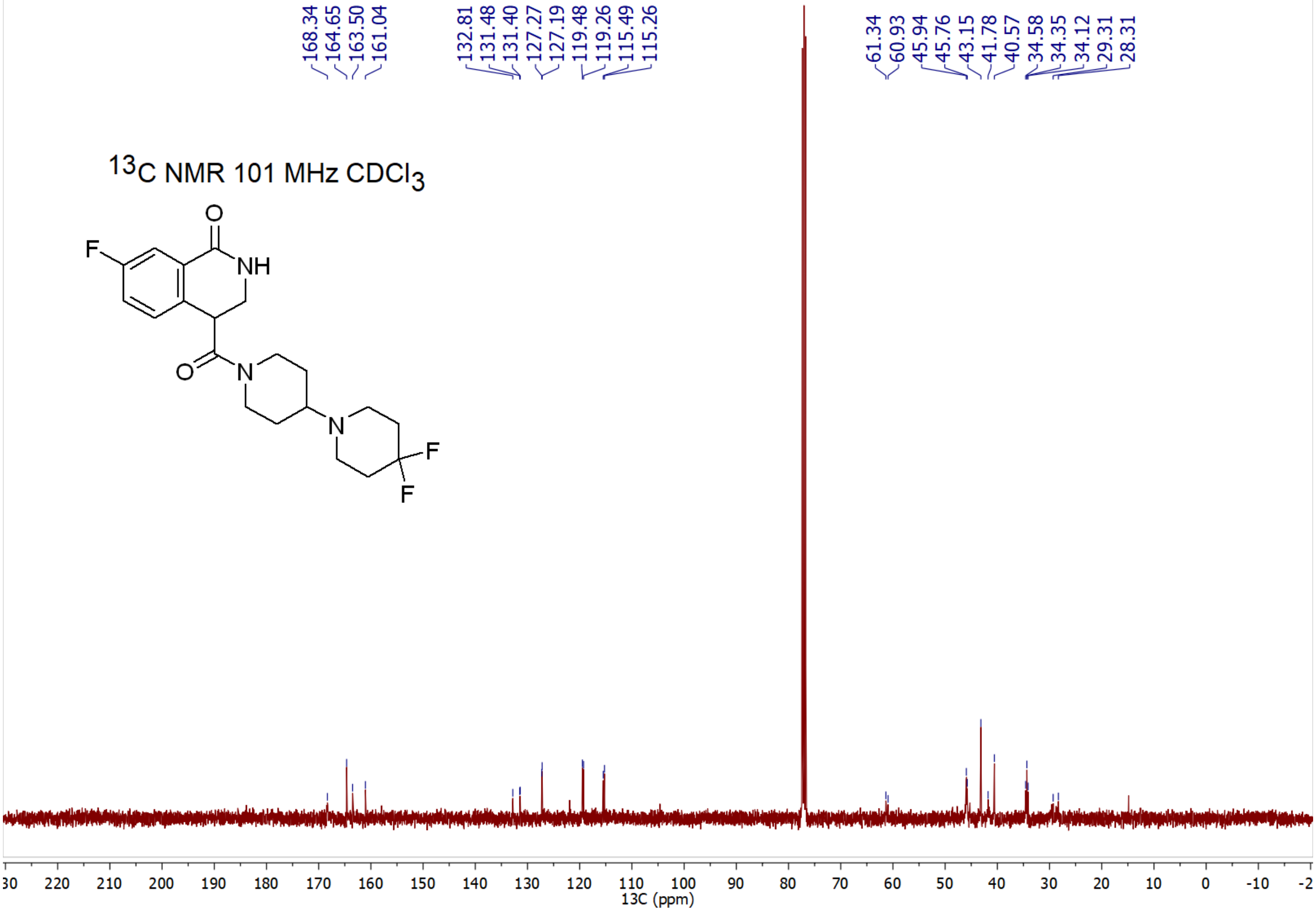

35. ^{1}H and ^{13}C NMR Spectra of **3ai**

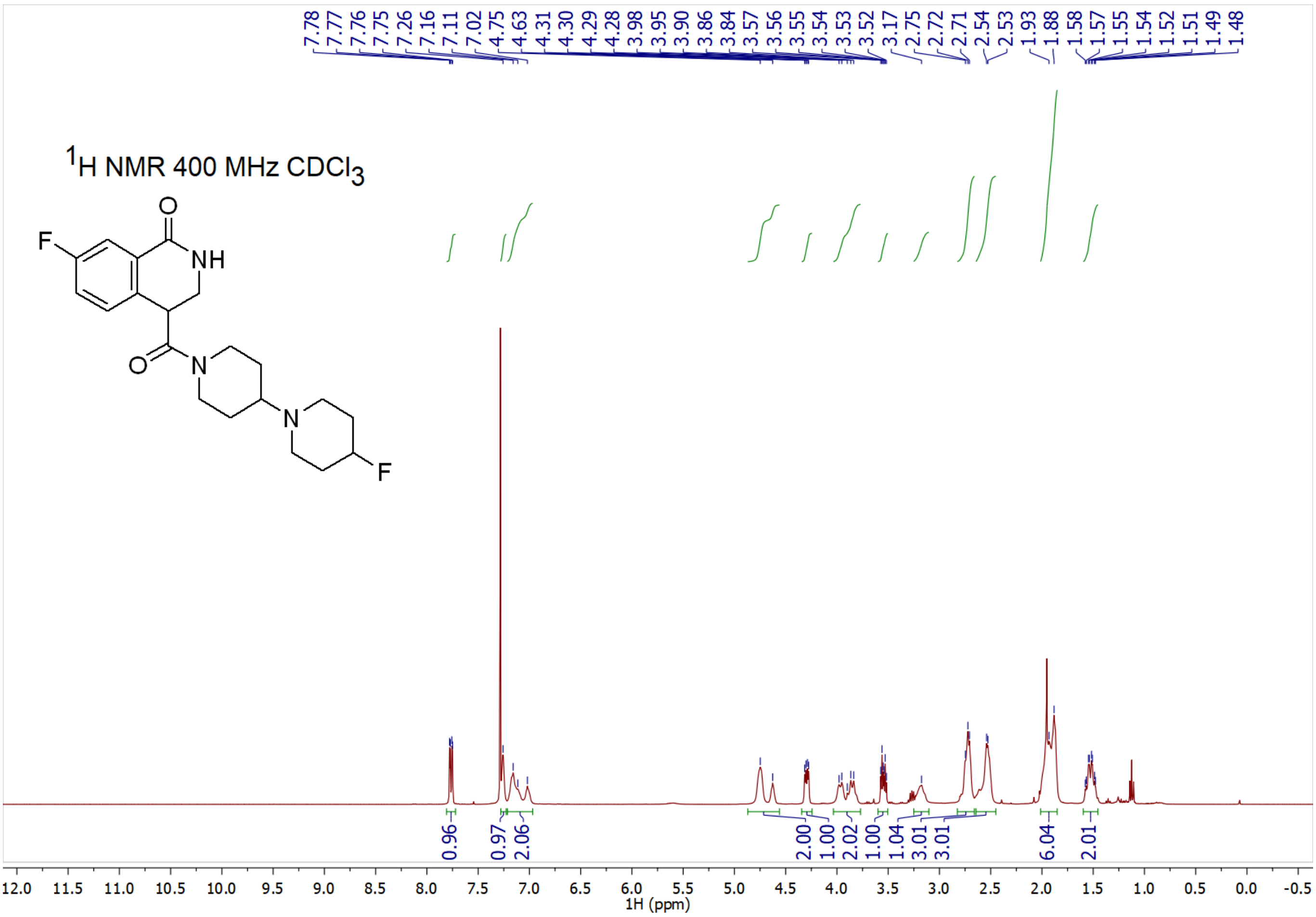

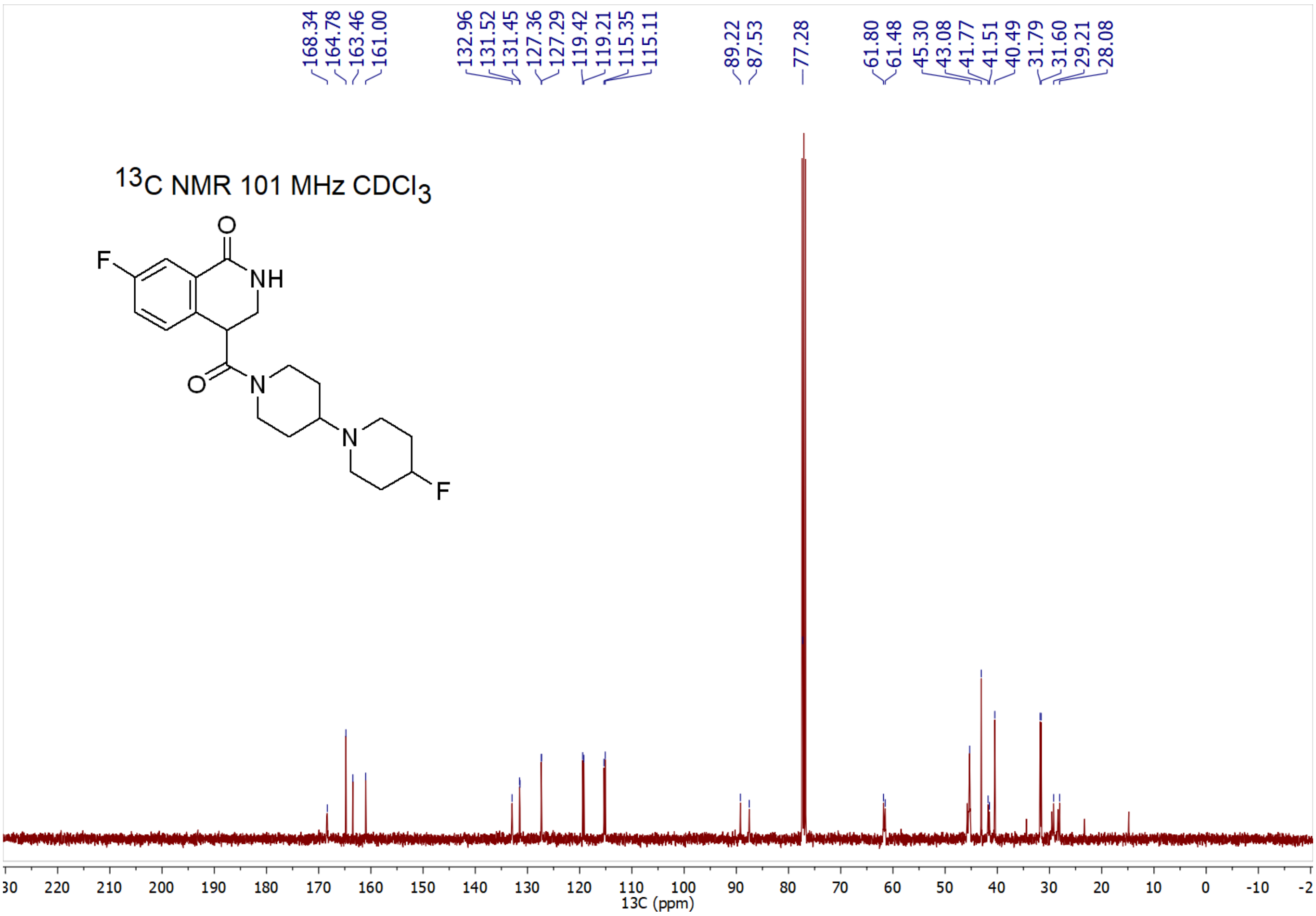

36. ^{1}H and ^{13}C NMR Spectra of **3aj**

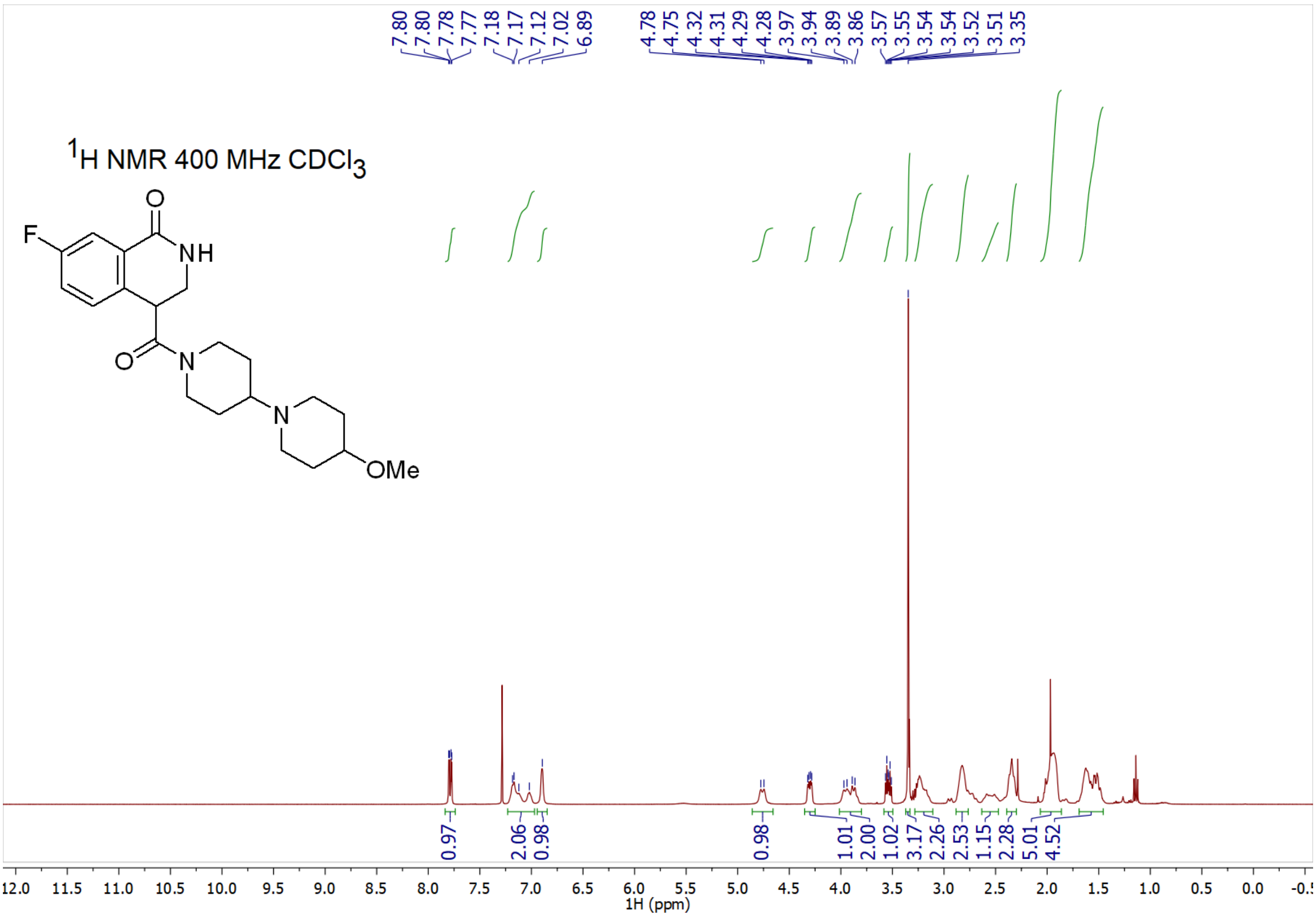

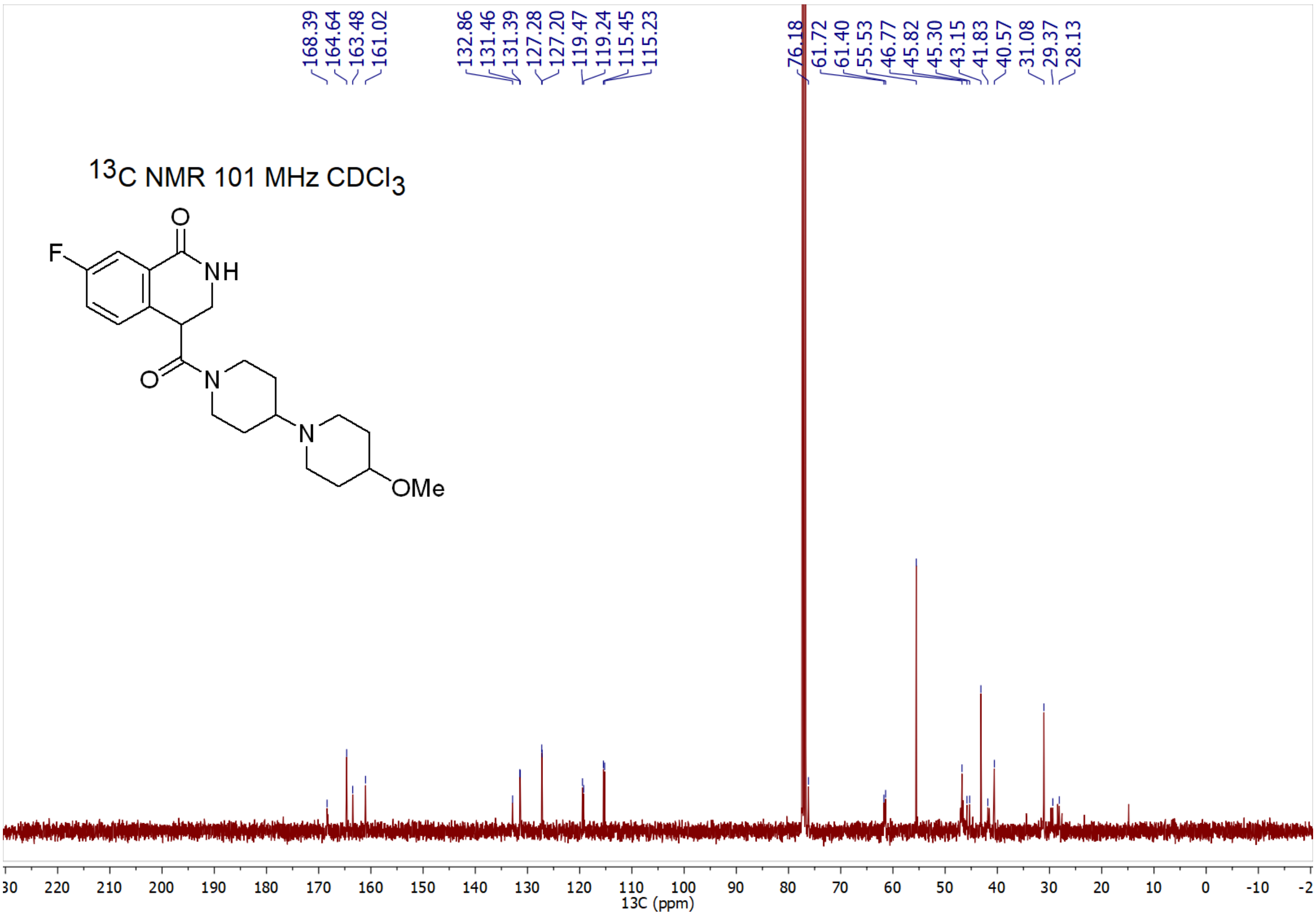

37. ^{1}H and ^{13}C NMR Spectra of **11**

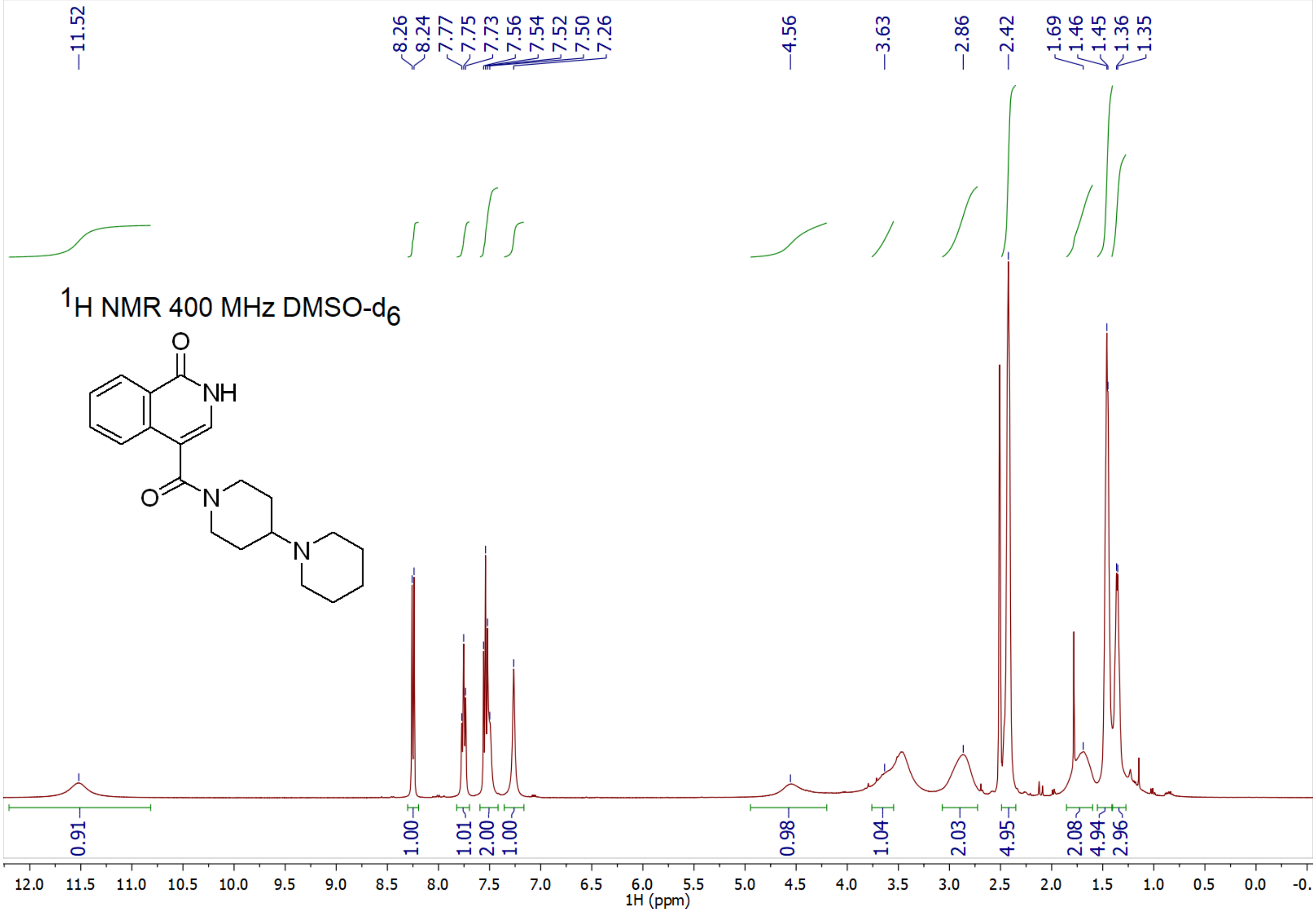

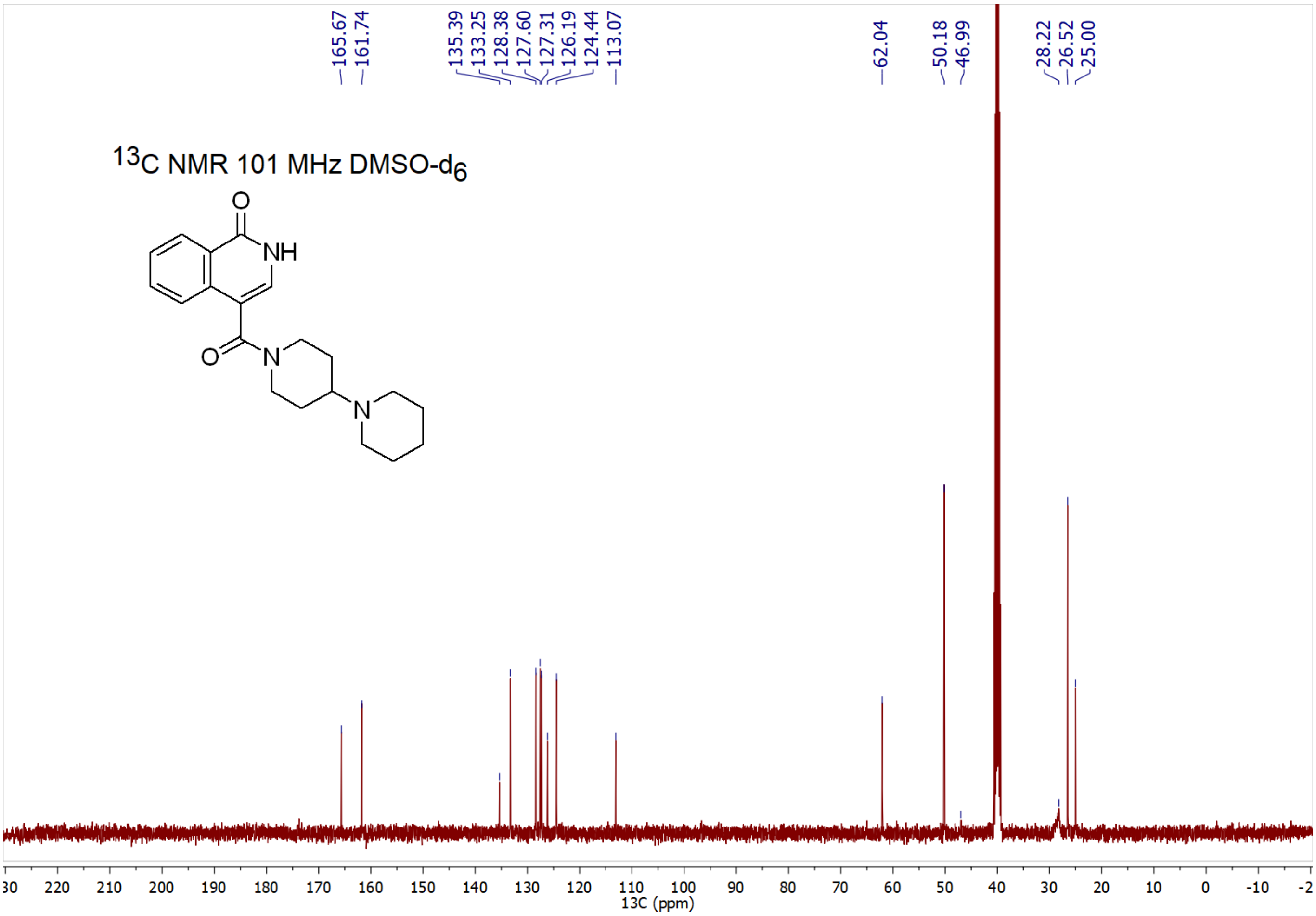

Supplement: Supplemental Material [file IENZ_A_1972993_SM5499.pdf]
